# Determination of the Experimental Minimal Formula of Metal‐Organic Frameworks

**DOI:** 10.1002/advs.202504713

**Published:** 2025-06-19

**Authors:** Jikson Pulparayil Mathew, Charlotte Simms, David E. Salazar Marcano, Evert Dhaene, Tatjana N. Parac‐Vogt, Jonathan De Roo

**Affiliations:** ^1^ Department of Chemistry University of Basel Mattenstrasse 22 4058 Basel Switzerland; ^2^ Department of Chemistry KU Leuven Celestijnenlaan 200F 3001 Leuven Belgium; ^3^ Department of Chemistry University of Antwerp Universiteitsplein 1 2610 Wilrijk Belgium

**Keywords:** MOFs, minimal formula, nuclear magnetic resonance (NMR), thermogravimetric analysis (TGA), chloride test, nitrate test

## Abstract

The determination of the precise chemical composition of metal organic frameworks (MOFs) is often overlooked, although it is crucial for many advanced applications such as catalysis or drug delivery. Here, a rigorous yet simple method is proposed for the accurate determination of a MOF's minimal formula. By combining quantitative nuclear magnetic resonance (NMR) and ultraviolet‐visible (UV–vis) spectroscopy data with thermogravimetric analysis (TGA), the minimal formula of several MOFs – MOF‐808(Zr), UiO‐66(Zr), UiO‐66(Ce), MOF‐5(Zn), MIL‐125(Ti), and MIL‐100(Fe) – are constructed. The influence of the MOF digestion method and the NMR measurement parameters on the accuracy of the minimal formula is shown. A quantitative method is provided for determining the amount of residual chloride or nitrate from metal precursors used in MOF synthesis, which has been ignored so far in minimal formulae determination. To improve the reproducibility and accuracy of MOF applications, the concept of the experimental molar mass is introduced, which can deviate significantly from the idealized molar mass. Although the determination of the MOF experimental minimal formula is often perceived as a complex and tedious task, the general methodology presented here is straightforward and involves very simple equations and procedures. It is easily generalized to new MOFs and even coordination networks of unknown structure, as demonstrated here for Al‐BDC.

## Graphical abstract

We present a simple yet rigorous method to determine the precise chemical composition of metal‐organic frameworks using Nuclear Magnetic Resonance (NMR), Thermogravimetric Analaysis (TGA), and ultraviolet‐visible (UV‐vis) spectroscopy. This approach reveals overlooked inorganic ligands and provides accurate experimental molar masses, enhancing reproducibility and applicability in fields such as catalysis. The method is broadly applicable, including amorphous materials.

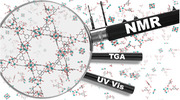

## 1 Introduction

**Figure 1:**
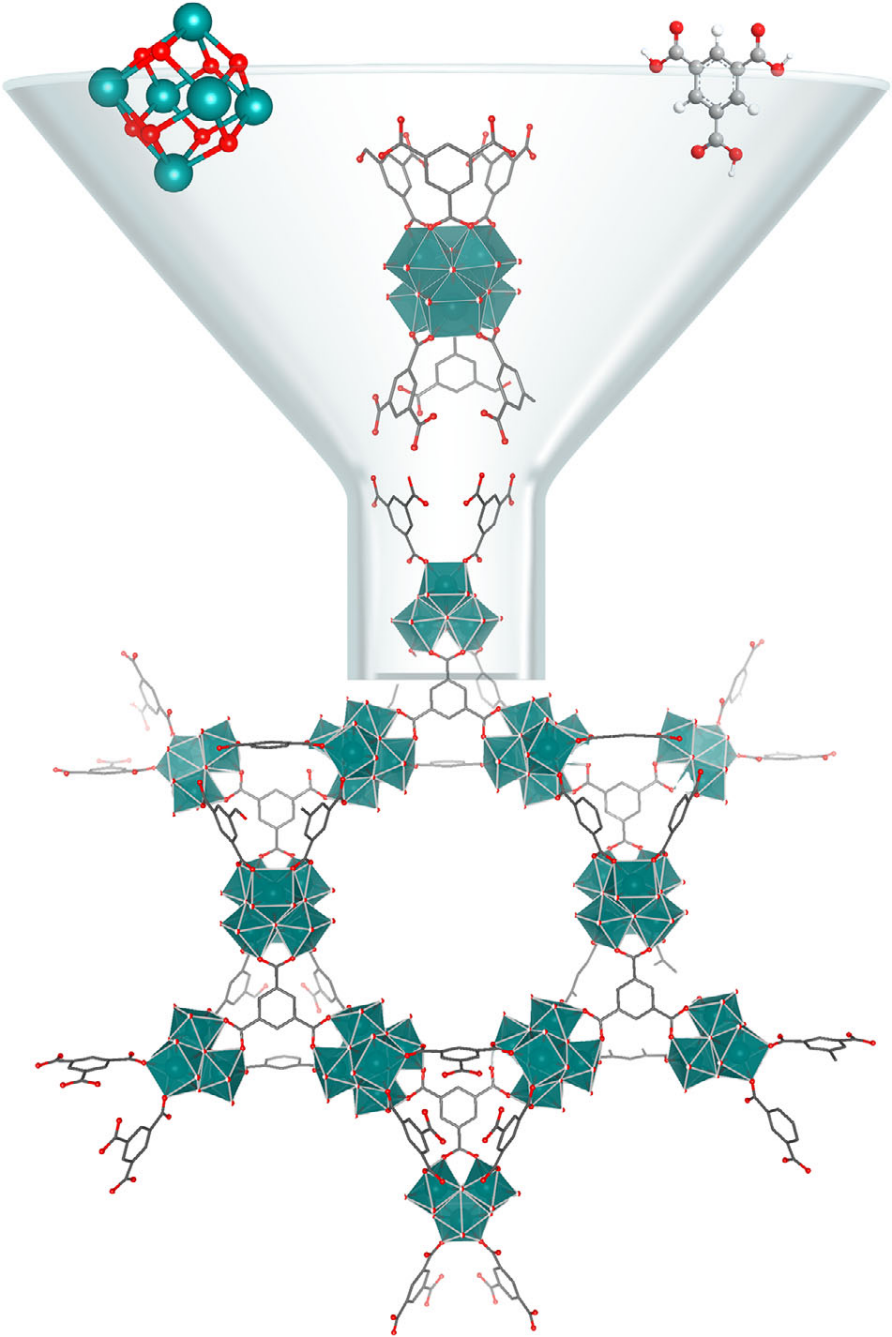
From the Zr_6_O_8_ SBU and benzene‐tricarboxylic acid linker, the MOF‐808 structure emerges. The SBU is also capped with four protons and six acetates, which are not shown here.

Metal‐organic frameworks (MOFs) are hybrid organic–inorganic materials with high porosity and structural tunability.^[^ ^1^ ^]^ Their versatility has made them useful in the fields of gas storage,^[^ ^2^, ^3^ ^]^ separation,^[^ ^4^, ^5^ ^]^ catalysis,^[^ ^6^, ^7^, ^8^ ^]^ sensing,^[^ ^9^, ^10^ ^]^ and drug delivery.^[^ ^11^, ^12^ ^]^ A MOF consists of two components: i) a metal‐containing node (the secondary building unit, SBU), which is formed of either a single metal ion or a larger metal‐oxo cluster (e.g., M_6_O_8_, M_3_O or M_8_O_8_) and ii) an organic linker molecule that bridges neighboring SBUs. The organic and inorganic components assemble into large crystalline 2D or 3D porous frameworks. This is represented in **Figure** **1** using MOF‐808 as an example. Through the careful choice of metal ion, and organic linker, many different MOF structures are possible,^[^ ^13^, ^14^ ^]^ with varying surface area,^[^ ^15^ ^]^ pore size,^[^ ^16^ ^]^ and connectivity (i.e., the number of linkers connected to each SBU). This huge flexibility in component choice has resulted in over 120'000 MOF structures being deposited into the Cambridge Crystallographic Data Centre (CCDC) to date.^[^ ^17^ ^]^

For molecular compounds, the chemical composition is given by the molecular formula. For extended solids, like MOFs, a minimal formula is used. However, real MOF structures deviate considerably from the idealized minimal formula. For instance, several moieties not typically included in the idealized formula of MOFs, such as modulating acids, co‐products, and side‐products of the synthesis,  may become part of the structure during the synthesis through coordination or H‐bonding interactions. For example, zirconium chlorides are often used as precursors for Zr‐MOFs (e.g., ZrCl_4_ or ZrOCl_2_) and HCl is commonly used as a crystallising agent. However, including chloride in the minimal formulae of Zr‐MOFs has been mostly overlooked until now. Yet, Taddei et al.^[^ ^18^ ^]^ identified the presence of chloride ions bound to the Zr centers of UiO‐66. Furthermore, MOFs often have missing‐linker or missing‐cluster defects that have a large impact on the properties of the MOF, particularly on their catalytic activity.^[^ ^7^, ^19^, ^20^, ^21^, ^22^, ^23^, ^24^, ^25^ ^]^ Therefore, establishing the experimental minimal formula is a critical step toward understanding the activity and behaviour of a MOF. Accurate minimal formulae are also important for a fair comparison between different MOFs or even between different batches of the same MOF. Unfortunately, the experimental minimal formulae of MOFs, including all moieties involved in the structure beyond the linkers, are typically not reported.

There are currently only a few reports on the minimal formula determination of MOFs,^[^ ^26^, ^27^, ^28^, ^29^ ^]^ with thermogravimetric analysis (TGA) as the primary characterisation technique and mostly focused on Zr‐MOFs. Indeed, this approach is limited to MOFs with clearly identifiable decomposition steps and intermediate plateaus, and involves relatively complex mathematical equations. Nuclear magnetic resonance (NMR) spectroscopy has been used as a complement to TGA for determining the modulator‐to‐linker ratio and for identifying residual solvents within the structure.^[^ ^29^ ^]^ However, the accuracy of the reported values is unclear since it critically depends on the measurement settings and the T_1_ relaxation behavior of the different resonances. Yet, the relevant delay times are only rarely reported.^[^ ^30^, ^31^ ^]^ In general, the error on the linker and modulator composition in the minimal formula are not discussed, even though the reported numbers suggest a high precision, e.g, Zr_6_O_4_(OH)_4_(BDC)_5.76_(FE)_0.48_ (where BDC is benzene dicarboxylate and FE is formate).^[^ ^29^ ^]^

Here, we show a convenient approach for obtaining the minimal formulae of MOFs, using NMR as the central technique and with MOF‐808 as a model system. We first show the main limitations of TGA, and we present our method of determining the experimental molar mass of a MOF. Second, we analyze the quantitative digestion of MOFs and find that formate detection can be an artifact of the digestion method. Third, we emphasize the importance of T_1_ relaxation times used in the NMR‐based quantification of linkers, or linker‐to‐modulator ratios. To encourage the appropriate use of delay times based on the T_1_ relaxation times, we provide measurement guidelines for a large variety of linkers and modulators. Lastly, we present UV–vis spectroscopy as a complementary technique that can be applied for chloride or nitrate quantification.^[^ ^32^ ^]^ We demonstrate that, using TGA, NMR and UV–vis spectroscopy it is possible to accurately determine the number of linkers, modulators, inorganic ligands, and solvents while taking into account all chemical boundary conditions concerning charge balance and coordination numbers to obtain the most precise minimal formula. Furthermore, we generalize our method to a large variety of MOFs: UiO‐66(Zr), UiO‐66(Ce), MOF‐5(Zn), MIL‐125(Ti), and MIL‐100(Fe).

## 2 Results

### 2.1 The Limitations of TGA

**Scheme 1:**
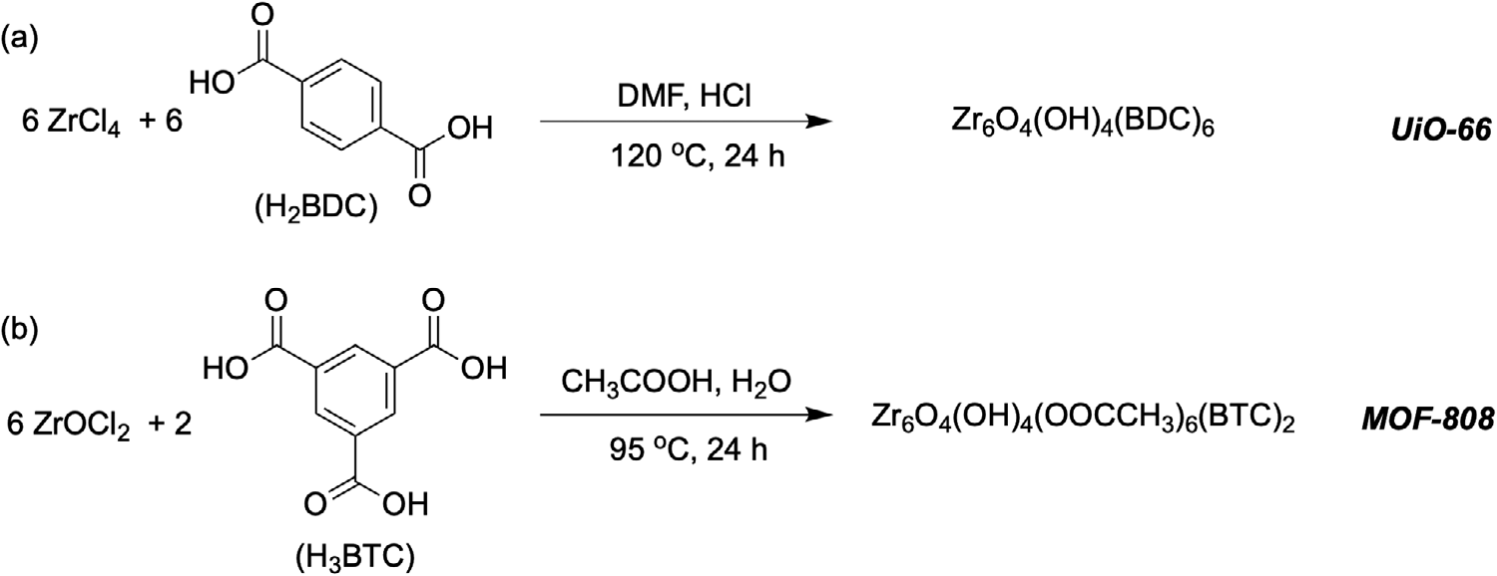
The synthesis of (a) UiO‐66(Zr) and (b) MOF‐808(Zr). Both structures are represented by their idealized minimal formula.

We synthesized UiO‐66(Zr) and MOF‐808(Zr) using 1,4‐benzene dicarboxylic acid (H_2_BDC) and 1,3,5‐benzene tricarboxylic acid (H_3_BTC) respectively, see **Scheme** **1**.^[^ ^33^, ^34^, ^35^ ^]^ The acidic modulators were respectively HCl or acetic acid. Once formed, the MOFs were washed thoroughly in a volatile solvent (acetone or methanol) and thermally activated at 110 °C for 20 h. The formation of the MOFs was confirmed from their characteristic Powder X‐ray Diffraction (pXRD) patterns and IR spectra (Figures S3– S6, Supporting Information).

**Figure 2:**
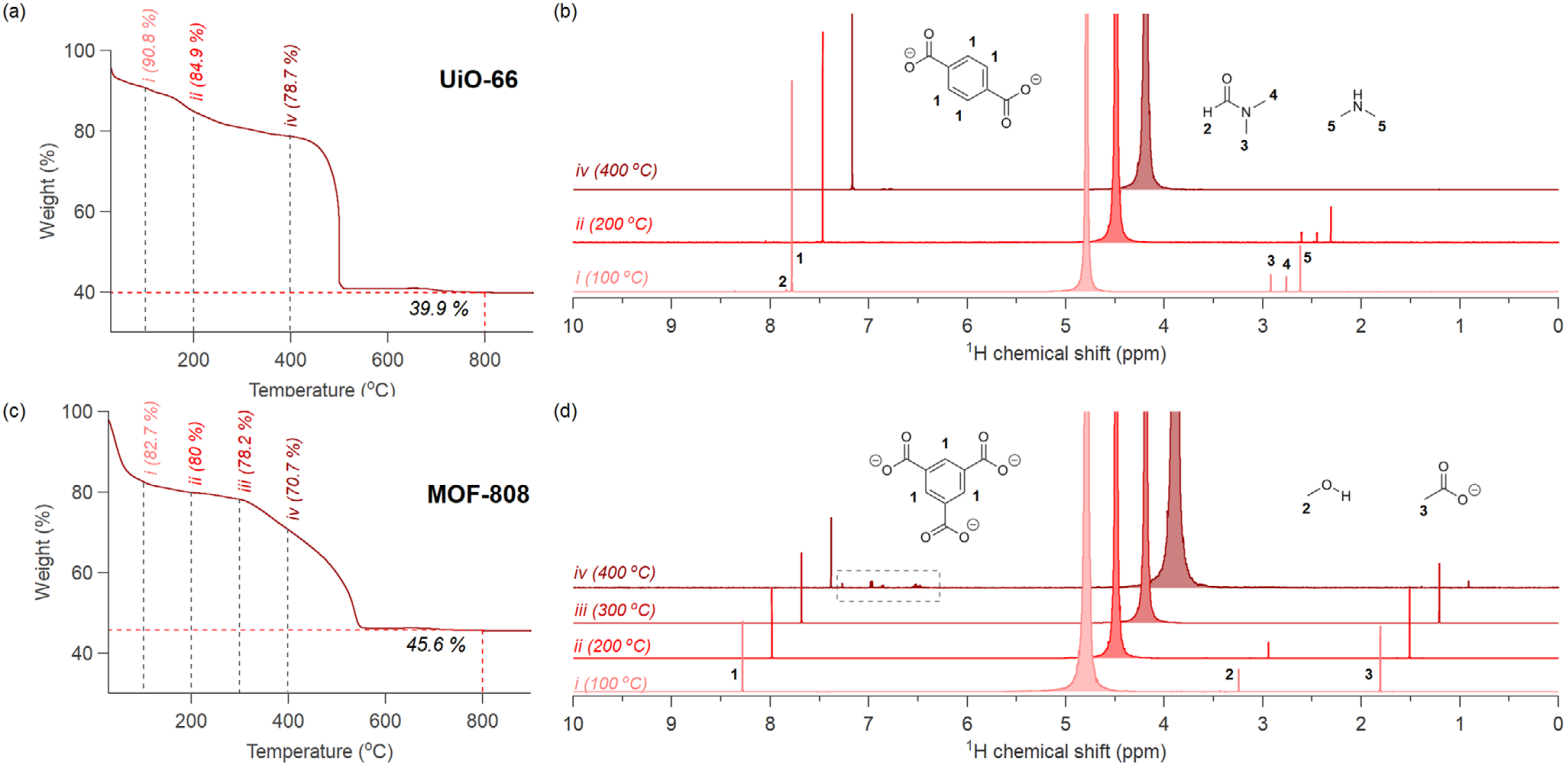
TGA and ^1^H NMR data of UiO‐66(Zr) (a, b) and MOF‐808(Zr) (c, d). The vertical lines (i, ii, iii, and iv) in the TGA correspond to 100, 200, 300, and 400 °C (at these temperatures MOF samples were digested and analyzed by NMR). The ^1^H NMR spectra correspond to solutions of the MOF digested in 1M NH_4_HCO_3_ in D_2_O after heating the activated MOF at 100, 200, 300, and 400 °C for 1 h. The NMR measurements were done quantitatively, with a sufficiently long relaxation delay (see below). The NMR spectra are displayed with a lateral offset of 0.3 ppm for clarity. The grey dotted box indicates unidentified aromatic decomposition products.

**Figure 3:**
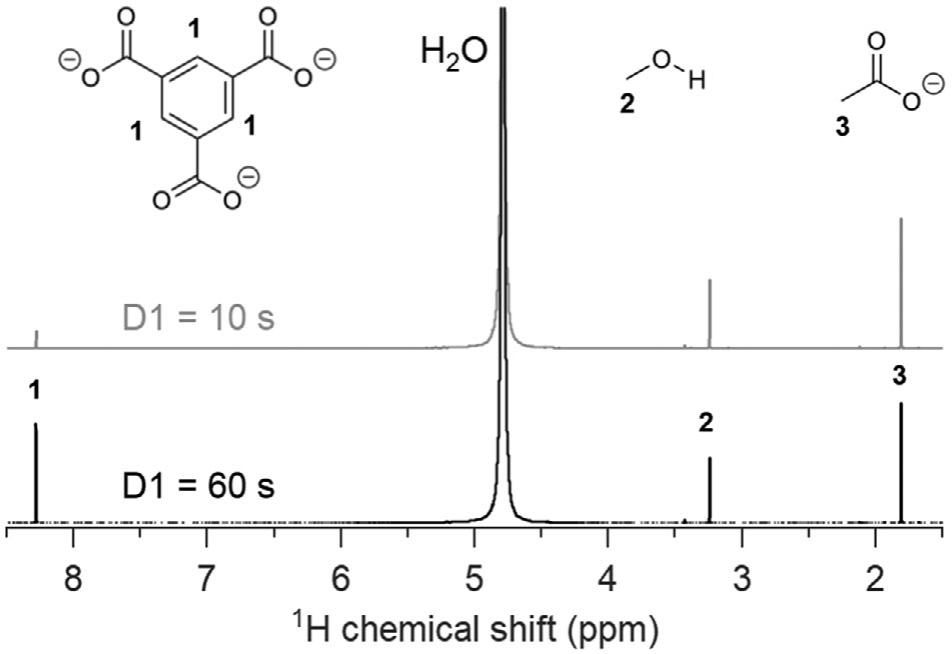
^1^H NMR spectra of MOF‐808(Zr) digested in 1M NH_4_HCO_3_, acquired with a relaxation delay of either 10 or 60 s.

**Figure 4:**
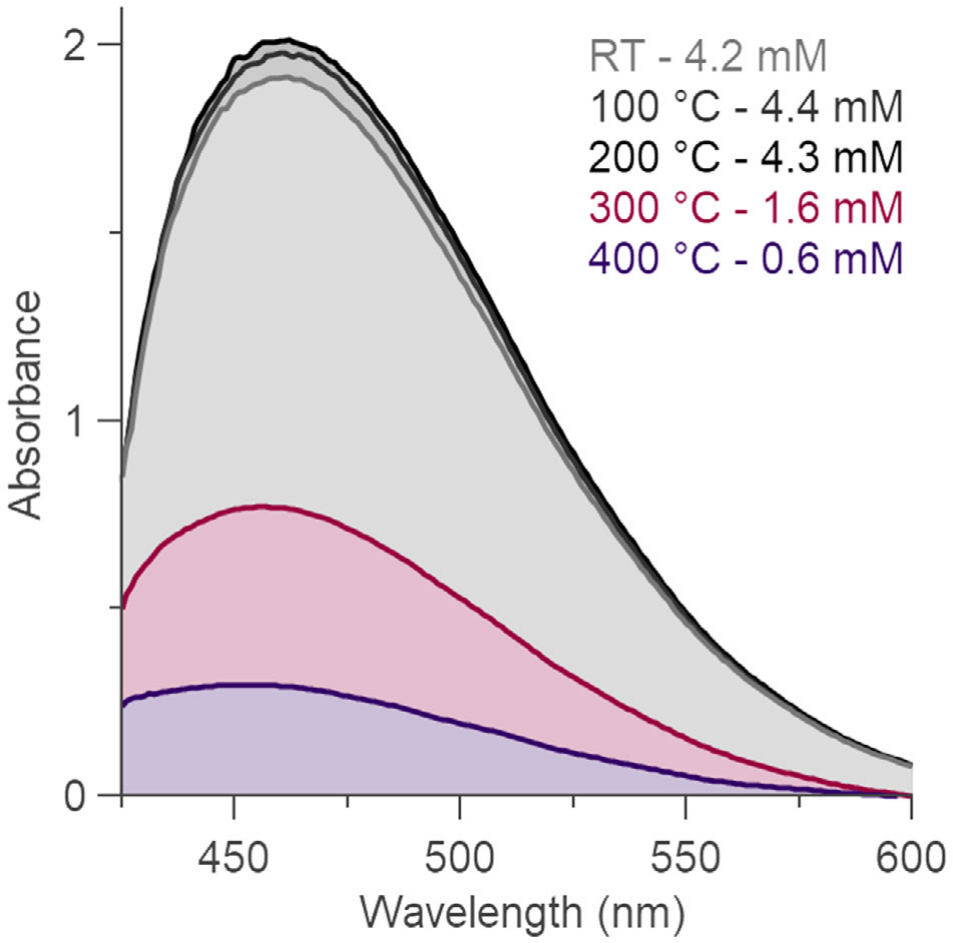
UV–vis absorption spectra of MOF‐808 sample after digestion in base and addition of the reagents for the chloride ion test. The spectra are taken before reactivation and also after reactivation of the sample at various temperatures for 1 h. The concentration of MOF‐808 is 1.5 M (calculated using the experimental molar mass determined by TGA).

**Figure 5:**
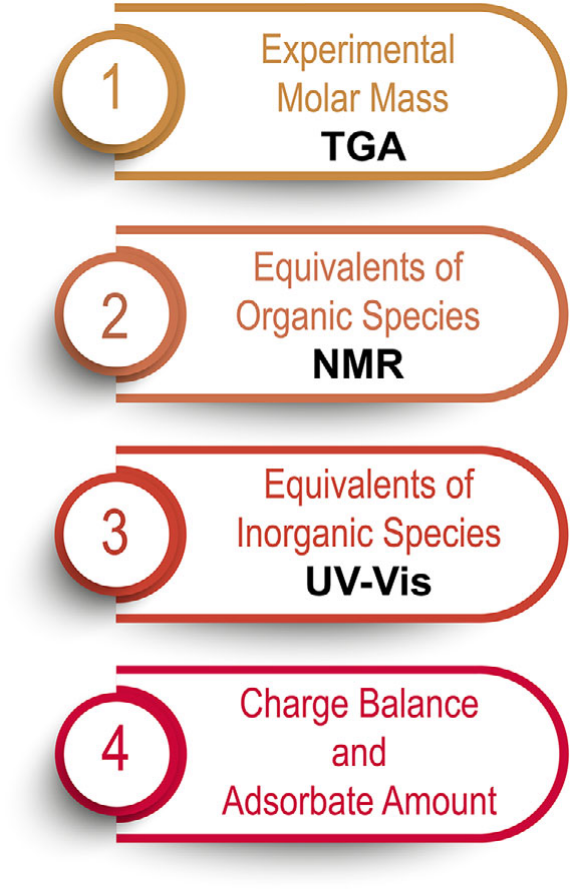
Schematic representation of the methodology used for determining the experimental minimal formula. Note: when determining the experimental molar mass, the nature of the oxide formed after combustion should be confirmed by pXRD if not known.

The MOFs were analysed using TGA, see **Figure** **2**. UiO‐66(Zr) presents an abrupt decomposition around 500 °C. In contrast to previous reports, our measurement does not feature a clear plateau between 300 and 400 °C, since we did not pre‐treat the sample at 270 °C (calcination procedure) before the measurement, as it is sometimes reported.^[^ ^26^ ^]^ The species at 400 °C was previously assigned to a dry MOF with a dehydrated cluster node, and was used to determine part of the minimal formula.^[^ ^26^ ^]^ In contrast, the decomposition of MOF‐808(Zr) is more gradual (between 350 and 550 °C) and a lot of adsorbed volatile species are removed below 200 °C.

To verify which organic species are lost at each temperature during TGA analysis, the MOFs were heated to different temperatures and the remaining solids underwent base digestion (see next section for details) and ^1^H NMR analysis (Figure 2). For UiO‐66(Zr), after heating to 100 °C, we identify the BDC linker, the synthesis solvent (DMF) and a decomposition product of DMF: dimethylamine (DMA).^[^ ^36^, ^37^, ^38^ ^]^ DMF and DMA are completely lost between 200 °C and 400 °C. The BDC linker remains in the structure until 400 °C. For MOF‐808, after heating to 100 °C, we identified the BTC linker, the washing solvent (methanol), and the coordinated acetate. We confirmed via solvent exchange with acetone that the detected methanol is simply trapped solvent in the pores (Figure S42, Supporting Information). While methanol is lost above 200 °C, acetate remains present until 400 °C. The linker‐to‐acetate ratio is 1:0.94 at 100 °C and remains constant until 200 °C (1:0.97). At 300 °C, the acetate content slightly decreases (1:0.78). At 400 °C, the acetate content is drastically reduced (1:0.12) but also the BTC linker starts to decompose, which is evidenced by additional aromatic signals between 7 and 8.5 ppm. To derive a minimal formula from TGA, one needs to identify a temperature where the MOF composition is known and only contains the linker. According to the NMR analysis, there is no such state for MOF‐808(Zr), and therefore one cannot derive the number of linkers from TGA alone. In addition, the presence of inorganic species such as chloride should also be considered (see further). For this reason, we developed a method that combines quantitative NMR (qNMR) analysis and TGA, further complemented by UV–vis spectroscopy.

### 2.2 Quantitative Linker Digestion

For qNMR analysis, we weigh out a precise amount of MOF and digest it in basic or acidic solutions. Subsequently, we determine the concentration of the species using 2 mM TMSP‐d_4_ (sodium 3‐(trimethylsilyl)propionate‐2,2,3,3‐d_4_) or 15.6 mM dimethylsulfone as an internal standard for base and acid digestion, respectively. We compared the efficiency and efficacy of three base digestion methods reported in the literature that used 1M NaOH,^[^ ^26^ ^]^ 1M NH_4_HCO_3_, or 1M NaHCO_3_ in D_2_O.^[^ ^39^ ^]^ All three yielded about the same concentration of linker, see **Table** **1**. The first method (using NaOH) resulted in precipitation of zirconium oxyhydroxides while the latter two (using bicarbonate salts) yielded clear solutions. Also, acid digestion (using conc. D_2_SO_4_ (96–98 wt. % in D_2_O))^[^ ^31^ ^]^ yielded a precipitate and a linker concentration comparable but slightly lower than base digestion. We prefer base digestion with carbonates given that complete digestion gives clear solutions that are most convenient for qNMR measurements.

**Table 1:** Concentration of BDC and BTC linkers in solution after digestion of the parent MOFs in different solutions, as determined by quantitative ^1^H NMR (D1 = 21 s for UiO‐66; D1 = 64 s for MOF‐808). The same amount of MOF was used in all the digestion protocols.

**MOF**	**Digestion solution**	**[Linker]**
**UiO‐66**	NaOH/D_2_O	7.3 mM
	NH_4_HCO_3_/D_2_O	7.3 mM
	NaHCO_3_/D_2_O	7.2 mM
	D_2_SO_4_/DMSO	7.0 mM
**MOF‐808**	NaOH/D_2_O	2.6 mM
	NH_4_HCO_3_/D_2_O	2.5 mM
	NaHCO_3_/D_2_O	2.5 mM
	D_2_SO_4_/DMSO	2.3 mM

While the amount of linker was the same regardless of the base used, we observed substantial differences in the other detected species after digestion. The UiO‐66 sample, digested in NaOH contains formate and DMA, but not DMF (Figure S43, Supporting Information).^[^ ^36^, ^40^ ^]^ When digesting UiO‐66 using bicarbonates, we do observe DMF and DMA but hardly any formate, as shown in Figure 2. We ascribed this to the different pH values of the digestion solutions (pH = 14 for NaOH and pH = 8.9 for NH_4_HCO_3_). DMF hydrolyzes to formate and DMA in 1M NaOH while it remains intact in 1M NH_4_HCO_3_, see Figure S44 (Supporting Information). Upon digestion in D_2_SO_4_/DMSO solution, the DMF also remains intact and we do not observe the formation of formic acid (Figure S45, Supporting Information). In literature, formate was detected in UiO‐66 (synthesized according to Scheme 1), and it was suggested that significant amounts of formate remain in the MOF structure by coordinating to zirconium.^[^ ^26^, ^41^ ^]^ Here we show that formate detection could be an artefact of the digestion method since its presence is dependent on the pH of the digestion solution. To further confirm this, we did an acetone wash of our UiO‐66(Zr) sample synthesized in DMF. The reactivated sample was then digested both in 1M NH_4_HCO_3_ and NaOH solutions. We observe that the DMF and formate peaks in the corresponding solutions become negligible after the wash, suggesting that the formate peak is indeed forming from the DMF that is present in the framework since it is no longer observed once DMF is removed from the pores (Figure S46, Supporting Information) while the structure of the MOF remains stable after the wash (Figure S47, Supporting Information). Note that the thermal decomposition of DMF (e.g., during MOF synthesis) is well described in organic chemistry; DMF decomposes at its boiling point into DMA and CO.^[^ ^42^, ^43^ ^]^ Hydrolysis of DMF into DMA and formate in the presence of sodium hydroxide has also been previously reported.^[^ ^44^ ^]^ In the presence of strong acids, DMF can hydrolyze (if water is present) to formic acid and DMA but the formic acid can be further catalytically decomposed to CO, CO_2_ and H_2_.^[^ ^43^ ^]^

### 2.3 The Influence of T_1_ Relaxation

To make NMR analysis quantitative, the delay time between scans needs to be sufficiently long to allow for full T_1_ relaxation of all resonances.^[^ ^45^ ^]^ This parameter is commonly overlooked in the MOF literature and delay times are either not mentioned or are too short to allow for full relaxation. This can have a dramatic influence on the apparent concentrations. **Figure** **3** shows NMR spectra of digested MOF‐808, measured with two different relaxation delay times (D1) of either 10 or 60 s. Strikingly the linker‐to‐acetate ratio is visibly different and changes from 0.125 (D1 = 10 s) to 0.83 (D1 = 60 s). A measurement with a short delay time thus significantly underestimates the linker concentration due to the relatively long T_1_ relaxation time of the linker. It is indeed expected for aromatic protons to have resonances with slow T_1_ relaxation, especially if there are no neighbouring hydrogens.

To provide guidance, we determined the T_1_ relaxation time for some of the most common carboxylate linkers present in MOFs, using an inversion recovery NMR experiment, see **Table** **2**. The T_1_ values were determined on a 600 MHz spectrometer for linkers in a 1M NH_4_HCO_3_ solution of D_2_O, which are representative of the conditions after digestion of a MOF. As can be seen from Table 2, aliphatic protons relax the fastest, aromatic protons are slower and isolated aromatic protons have the slowest T_1_ relaxation. The recommended delay time in a quantitative ^1^H NMR experiment (with a 90 degree pulse), is five times the T_1_ relaxation time. For an experiment with a 30 degree pulse (as often used in automated NMR experiments), the delay time can be shorter; three times the T_1_. In real samples, there is usually a mixture of organic compounds. For example, the relaxation time of the acetic acid modulator is 5.5 s (D1 = 27.5 s) and the formic acid modulator is 20 s (D1 = 100 s) while the relaxation time for BTC is 8.6 s (D1 = 43 s) and for BDC it is 3.7 s (D1 = 18.5 s). The delay time for the NMR measurement must be selected based on the T_1_ of the slowest relaxing species in the sample. To quantify the solvent molecules trapped in the pores, their T_1_ relaxation time must also be taken into consideration. An overview of the T_1_ relaxation times for the different modulators, solvents and the internal standard is provided in Table S1 (Supporting Information).

**Table 2:** T_1_ relaxation time and recommended relaxation delay (D1) of common carboxylate linker molecules determined by ^1^H NMR on a 600 MHz spectrometer in a 1M NH_4_HCO_3_ solution in D_2_O. The NMR spectra are calibrated based on the D_2_O peak (4.79 ppm).

**Entry**	**Molecule**	**Chemical Shift (ppm)**	**T_1_**	**D1**
	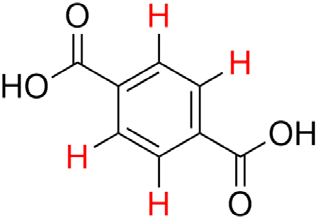			
1		7.7	3.7 s	18.5 s
	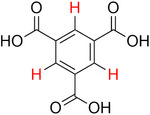			
2		8.3	8.6 s	43 s
	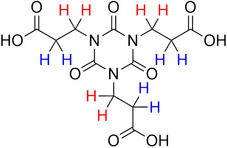			
3		3.9	0.5 s	2.5 s
		2.3	0.6 s	3 s
	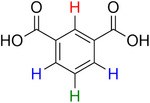			
4		8.1	11.6 s	58 s
		7.8	4.8 s	24 s
		7.4	2.5 s	12.5 s
	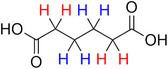			
5		2.1	1.3 s	6.5 s
		1.4	1.1 s	5.5 s
	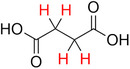			
6		2.2	2.3 s	11.5 s
	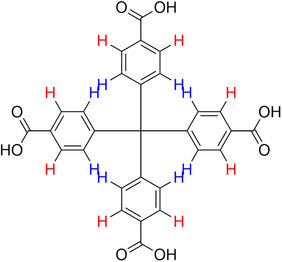			
7		7.5	2.7 s	13.5 s
		7.3	1.6 s	8 s
	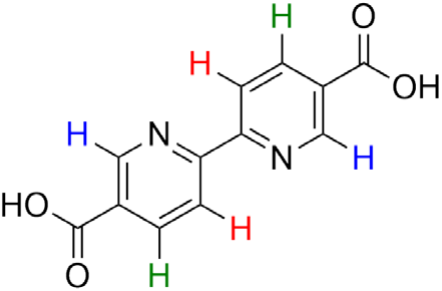			
8		8.8	0.8 s	4 s
		8.2	1.2 s	6 s
		7.9	0.8 s	4 s
	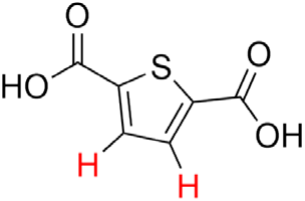			
9		7.4	5.2 s	26 s
	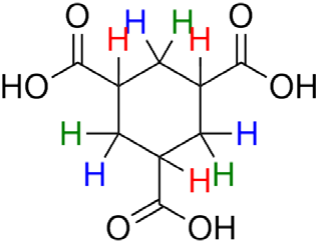			
10		2.1	1 s	5 s
		1.9	0.5 s	2.5 s
		1.2	0.5 s	2.5 s
	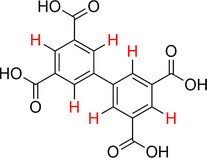			
11		8.2	2.7 s	13.5 s
	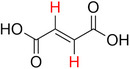			
12		6.4	12 s	60 s
	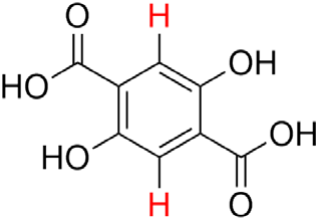			
13		7.2	11.5 s	57.5 s

^1^ Terephthalic acid; ^2^ Trimesic acid; ^3^  3,3',3”‐(2,4,6‐Trioxo‐1,3,5‐triazinane‐1,3,5‐triyl)tripropionic acid; ^4^  Isophthalic acid; ^5^  Adipic acid; ^6^  Succinic acid; ^7^  4,4',4'',4'''‐Methanetetrayltetrabenzoic acid; ^8^  2,2'‐Bipyridine‐5,5'‐dicarboxylic acid; ^9^  2,5‐Thiophenedicarboxylic acid; ^10^  Cyclohexane‐1,3,5‐tricarboxylic acid; ^11^  Biphenyl‐3,3',5,5'‐tetracarboxylic acid; ^12^  Fumaric acid; ^13^  2,5‐Dihydroxy‐1,4‐benzene dicarboxylic acid

### 2.4 Determination of Residual Inorganic Ligands

Apart from organic linkers and modulators, inorganic ligands (e.g., chloride, fluoride, nitrate, …) from the synthetic precursors can also bind to the metals in the nodes of the MOF. Although it was previously shown that chloride remains present in the MOF,^[^ ^18^ ^]^ it has so far not been included in the minimal formula determination. The presence of Cl in MOF‐808(Zr) and UiO‐66(Zr) can be clearly observed in STEM‐EDX mapping (Figures S1 and S2, Supporting Information). The characteristic peaks at 2.62 and 2.81 keV correspond to the Kα and Kβ emission lines of Cl. For quantification, we used the commercially available Spectroquant Chloride Test to photometrically determine the amount of chloride ions present in digested MOF solutions. This test involves the sequential addition of mercury (II) thiocyanate and iron (III) ions. If there are chloride ions present, they react with mercury thiocyanate to form mercury chloride, releasing thiocyanate. The latter then reacts with iron ions to form iron thiocyanate, which shows a strong UV–vis absorption band. This photometric determination of chloride is robust and unaffected by slight variations in pH or the presence of metal ions, such as Zr^4+^, or linkers (i.e., BDC or BTC) in the MOF digestion solution (Figure S15, Supporting Information). A detailed protocol is provided in the Supporting Information and is also available from the commercial supplier.

The chloride content of MOFs after heating at different temperatures was determined by UV–vis (**Figure** **4**) to find out when the chlorides in the MOF are lost during TGA measurements. The chloride content of MOF‐808 remains constant until 200 °C, and then starts decreasing. At 400 °C, a significant amount of chloride was removed, but there is still some remaining, suggesting that the chloride content still contributes towards the MOF weight up to 400 °C. Very similar results are obtained for the case of UiO‐66 (Figure S19, Supporting Information). The persistence of chloride in the MOF is also evident from the fact that the chloride content remains relatively unchanged after washing the MOFs for several hours in acetone, which removes most organic molecules trapped in the pores (Figure S53, Supporting Information). This suggests that chloride indeed acts as an inorganic ligand that binds to the metals in the MOF.

In a similar way, the presence of nitrates in MOFs can be determined photometrically using a similar commercial Spectroquant Nitrate Test. In this case, the nitration of 2,6‐dimethylphenol to form 4‐nitro‐2,6‐dimethylphenol under acidic conditions can be followed photometrically. The amount of 4‐nitro‐2,6‐dimethylphenol formed, which gives rise to a distinct absorption band at around 340 nm, corresponds to the amount of nitrate ions present in solution. This nitrate quantification method can also be used in the presence of metal ions and MOF linkers without significantly affecting its accuracy (Figure S16, Supporting Information). A detailed protocol is provided in the Supporting Information and is also available from the commercial supplier.

### 2.5 The Minimal Formula

We will now demonstrate the derivation of the minimal formula from TGA, NMR and UV–vis data using the example of MOF‐808(Zr). MOF‐808(Zr) has been scarcely investigated in the context of experimentally determining its minimal formula, since previous methods have mainly focused on UiO‐66(Zr).^[^ ^26^, ^27^, ^29^ ^]^ Nevertheless, to demonstrate the generality of this method for different MOF structures, we also determined the experimental minimal formula of UiO‐66(Zr), UiO‐66(Ce), MOF‐5(Zn), MIL‐125(Ti), and MIL‐100(Fe) (see Generalization and Outlook section below and Supporting Information).

Given the qualitative analysis above, we write the minimal formula of MOF‐808(Zr) as ${\mathrm{Zr}}_{6}O_{4}(\mu_{3}$‐OH)_4_(OH)_*w*_(BTC)_*x*_(OAc)_*y*_(Cl)_*i*_(MeOH)_*z*_(H_2_O)_*n*_. In the first step towards determining all the variables, we calculate the molar mass of the MOF using TGA. Upon full combustion, the MOF turns into zirconium dioxide, carbon dioxide, and water.^[^ ^46^ ^]^ This was confirmed from the pXRD pattern of the residual solid after heating up to 900 °C, which corresponds to the monoclinic structure of ZrO_2_ (Figure S41, Supporting Information), and from the EDX spectrum, which has mainly peaks corresponding to Zr and O, but no Cl peaks (Figure S1, Supporting Information). Therefore, the combustion equation for the idealized MOF is:

(1) $$\begin{array}{ccc} & & {\mathrm{Zr}}_{6}O_{4}{\left(\mathrm{OH}\right)}_{4}{\left(\mathrm{OOC}{\mathrm{CH}}_{3}\right)}_{6}{\left({\left(\mathrm{OOC}\right)}_{3}C_{6}H_{3}\right)}_{2}+27O_{2}\\ & & \;\overset{\Delta }{\to }6{\mathrm{ZrO}}_{2}+30{\mathrm{CO}}_{2}+14H_{2}O \end{array}$$

One mole of Zr_6_ cluster turns into 6 moles of ZrO_2_, therefore:

(2) $$6n_{{\mathrm{Zr}}_{6}}=n_{{\mathrm{ZrO}}_{2}}$$

The number of moles is *n* = *W*/*M*, with *M* as the molar mass and W as the weight or mass of the compound. Hence, from the initial weight (*W*_*i*_) and the final weight after combustion ($W_{{\mathrm{ZrO}}_{2}}$) determined by TGA, we can extract the experimental molar mass of the MOF (*M*_*exp*_) by rewriting Equation (2).

(3) $$6\frac{W_{i}}{M_{exp}}=\frac{W_{{\mathrm{ZrO}}_{2}}}{M_{{\mathrm{ZrO}}_{2}}}$$

After rearranging, this becomes:

(4) $$M_{exp}=\frac{W_{i}}{W_{{\mathrm{ZrO}}_{2}}}\times 6\;M_{{\mathrm{ZrO}}_{2}}=\frac{100\%}{W_{{\mathrm{ZrO}}_{2}}}\times 739.3$$

The molar mass of 6 ZrO_2_ units ($6M_{{\mathrm{ZrO}}_{2}}$) is 739.3 g mol^−1^ and *W*_*i*_ is set at 100%. According to Equation (4), we thus easily determine the molar mass of the MOF under the experimental lab conditions (e.g., humidity, temperature). From the ideal formula, Zr_6_O_4_(OH)_4_(BTC)_2_(OAc)_6_, the expected molar mass is 1447.9 g mol^−1^. However, we determined an experimental molar mass of 1619.5 g mol^−1^ based on a residual weight ($W_{{\mathrm{ZrO}}_{2}}$) of 45.6%. The higher experimental value can be attributed to the absorption of water from the atmosphere into the pores of the MOF. This is further demonstrated by the fast weight loss in TGA below 200 °C. We find the experimental molar mass to be an extremely useful number as this is the experimentally relevant number when weighing out MOFs in regular lab conditions. Using any other number would result in incorrect equivalents of MOF and e.g., in wrong turnover numbers in catalysis.

In a second step, we digested 0.75 μmol MOF (calculated using the experimental molar mass) in 1 M NH_4_HCO_3_ in D_2_O and added the TMSP‐d_4_ internal standard. The final concentration of MOF‐808 in the NMR sample was 1.5 mM and the concentration of TMSP‐d_4_ was 2 mM. A ^1^H NMR spectrum of this digested solution was then recorded with a sufficiently long delay time and the integral of  TMSP‐d_4_ was set at nine, reflecting the presence of nine protons in this standard (Figure S49, Supporting Information). The concentration of BTC, acetate and methanol was calculated based on the relative integration of their respective peaks, taking into account the number of protons that give rise to each peak: 3H for BTC, 3H for acetate, 3H for methanol and 9H for TMSP‐d_4_.

(5) $$\begin{array}{cc} \left[BTC\right] & =\frac{I_{BTC}}{3}\times \left[{\mathrm{TMSP}-d}_{4}\right]\\ \left[OAc\right] & =\frac{I_{OAc}}{3}\times \left[{\mathrm{TMSP}-d}_{4}\right]\\ \left[MeOH\right] & =\frac{I_{MeOH}}{3}\times \left[{\mathrm{TMSP}-d}_{4}\right] \end{array}$$

We find a BTC concentration of 3 mM, an acetate concentration of 3 mM, and a methanol concentration of 0.4 mM. Given that the MOF concentration was 1.5 mM based on the experimental molar mass determined by TGA, the amount of BTC (x), acetate (y) and methanol (z) in the experimental minimal formula, (${\mathrm{Zr}}_{6}O_{4}(\mu_{3}$‐OH)_4_(OH)_w_(BTC)_x_(OAc)_y_(Cl)_i_(MeOH)_z_(H_2_O)_n_), can be easily calculated:

(6) $$\begin{array}{cc} x & =\frac{\left[BTC\right]}{\left[MOF-808\right]}=\frac{3.0}{1.5}=2\\ y & =\frac{\left[AcOH\right]}{\left[MOF-808\right]}=\frac{3.0}{1.5}=2\\ z & =\frac{\left[MeOH\right]}{\left[MOF-808\right]}=\frac{0.4}{1.5}=0.3 \end{array}$$

In the third step, we determine the amount of chloride in the sample. For this, we turn to UV–vis analysis. We made a MOF‐808 solution of 1.5 mM (calculated using the experimental molar mass determined by TGA). Based on the UV–vis absorption at 500 nm after adding the reagents from the Spectroquant Chloride Test, the chloride concentration was determined to be 4.2 mM for the MOF‐808 sample (without any reactivation) (Figure 4). From this concentration, we can then calculate the *i* variable in the minimal formula as

(7) $$i=\frac{\left[Cl\right]}{\left[MOF-808\right]}=\frac{4.2}{1.5}=2.8$$

Finally, the charge in the minimal formula needs to be balanced with bound hydroxide ligands. Some researchers have proposed to replace hydroxide with oxide to increase the negative charge,^[^ ^13^ ^]^ but that would lead to coordinatively unsaturated zirconium ions, reducing the stability of the cluster and, therefore, of the overall MOF structure. Therefore, we consider it more plausible to compensate with extra hydroxide species as is commonly proposed in the qualitative MOF literature.^[^ ^26^, ^47^, ^48^, ^49^ ^]^ In total, the six zirconium cations are charge balanced by the sum of all the anionic species:

(8) $$\begin{array}{cc} C^{Zr^{4+}} & =C^{O^{2-}}+C^{OH^{-}}+C^{BTC^{3-}}+C^{OAc^{-}}+C^{Cl^{-}}\\ 6\times 4 & =4\times 2+4+w+3x+y+i\\ 24 & =12+w+(3\times 2)+2+2.8\\ w & =1.2 \end{array}$$

Overall, we arrive at the minimal formula: ${\mathrm{Zr}}_{6}O_{4}(\mu_{3}$‐OH)_4_(OH)_1.2_(BTC)_2_(OAc)_2_(Cl)_2.8_(MeOH)_0.3_.

For this MOF, no detectable linker defects were observed within the precision of the current experimental methods. In addition, acetate has a lower stoichiometry compared to the ideal formula and is replaced with either chloride or hydroxide. The molar mass of this minimal formula is 1341 g mol^−1^, which is reasonably close to the molar mass determined by TGA after a dehydration step at 100 °C: 1320 g mol^−1^ (Figure S50, Supporting Information), especially considering the discrepancy with the experimental molar mass (1620 g mol^−1^). This is validation that the above minimal formula is representative of the structure of the dry MOF.

Finally, we consider whether the ligands in the minimal formula result in coordinatively saturated Zr centres. Hydroxide and chloride are monodentate ligands, in contrast to the bridging carboxylates. Therefore, we hypothesize that the MOF contains one coordinated water molecule for every monodentate ligand (1.2 + 2.8 = 4): ${\mathrm{Zr}}_{6}O_{4}(\mu_{3}$‐OH)_4_(OH)_1.2_(BTC)_2_(OAc)_2_(Cl)_2.8_(MeOH)_0.3_(H_2_O)_4_

In addition, MOFs are known to physisorb water from the air into their pores. The presence of adsorbed water was confirmed by TGA‐MS analysis of MOF‐808, which showed that only water is present in the pores and CO_2_ is only observed once combustion of the MOF begins (Figure S51, Supporting Information). Hence, the total amount of adsorbed water can be determined from the difference between the experimental molar mass (*M*_*exp*_ = 1619.5 g mol^−1^) and the molar mass of the anhydrous minimal formula obtained so far (*M*_*anhy*_ = 1341 g mol^−1^), taking into account the molar mass of water (18 g mol^−1^).

(9) $$n=\frac{M_{\exp}-M_{\mathrm{anhy}}}{18\;{g\;\mathrm{mol}}^{-1}}=15.5$$

There are thus 15.5 water molecules per formula unit, of which four are coordinated and 11.5 are physisorbed. The experimental minimal formula of the MOF under ambient conditions can be written as: ${\mathrm{Zr}}_{6}O_{4}(\mu_{3}$‐OH)_4_(OH)_1.2_(BTC)_2_(OAc)_2_(Cl)_2.8_(MeOH)_0.3_(H_2_O)_15.5_.

The MOF can be described by three different minimal formulae: i) the ideal formula, ii) the experimental minimal formula of the MOF without adsorbates, and iii) the experimental minimal formula including water and other molecules (e.g., methanol) adsorbed in the pores. The latter is the most useful to determine the molar amount of a MOF by simply weighing the MOF under experimental lab conditions. The molar mass and percentage of Zr (by mass) of MOF‐808 varies from 37.8 wt.% Zr in the ideal formula vs 33.8 wt.% in the experimental formula from the MOF under actual laboratory working conditions (**Table** **3**). This highlights the importance of determining the experimental minimal formula. For instance, the amount of metal could be under‐ or over‐estimated by relying on the ideal minimal formula, which is particularly important when investigating the catalytic properties of MOFs.

**Table 3:** Molar mass (M_*w*_) and percentage of metal by mass (% M) for the ideal minimal formula (top) of all MOFs used in this work compared to the experimental minimal formulae of the hydrated MOFs (bottom).

**MOF**	**Formula**	**M_*w*_ (g mol^−1^)**	**% M**
**MOF‐808**	${\mathrm{Zr}}_{6}O_{4}(\mu_{3}$‐OH)_4_(BTC)_2_(OAc)_6_	1447.9	37.8
	${\mathrm{Zr}}_{6}O_{4}(\mu_{3}$‐OH)_4_(OH)_1.2_(BTC)_2_(OAc)_2_(Cl)_2.8_(MeOH)_0.3_(H_2_O)_15.5_	1620.2	33.8
**UiO‐66(Zr)**	${\mathrm{Zr}}_{6}O_{4}(\mu_{3}$‐OH)_4_(BDC)_6_	1664.1	32.9
	${\mathrm{Zr}}_{6}O_{4}(\mu_{3}$‐OH)_4_(BDC)_5.1_(Cl)_1.8_(DMACl)_0.9_(DMF)_0.8_(H_2_O)_7.8_	1852.6	29.6
**UiO‐66(Ce)**	${\mathrm{Ce}}_{6}O_{4}(\mu_{3}$‐OH)_4_(BDC)_6_	1957.4	42.9
	${\mathrm{Ce}}_{6}O_{4}(\mu_{3}$‐OH)_4_(OH)_0.9_(BDC)_4.5_(NO_3_)_2.1_(DMF)_1.9_(EtOH)_1.1_(H_2_O)_13.2_	2284.1	36.8
**MOF‐5**	Zn_4_O(BDC)_3_	769.9	34.0
	Zn_4_O(BDC)_3_(H_2_O)_1.5_	796.9	32.8
**MIL‐125**	Ti_8_O_8_(OH)_4_(BDC)_6_	1563.7	24.5
	Ti_8_O_8_(OH)_4_(BDC)_6_(EtOH)_0.5_(DMA)_2_(TMA)_0.6_(H_2_O)_22.4_	2115.9	18.1
**MIL‐100**	Fe_3_O(OH)(H_2_O)_2_(BTC)_2_	650.8	25.7
	Fe_3_O(OH)_0.2_(BTC)_2_(Cl)_0.8_(MeOH)_1.8_(EtOH)_0.3_(H_2_O)_6.8_	823.5	20.3
MIL‐53	Al(OH)(BDC)	208.1	13.0
**Al‐BDC**	Al(OH)(BDC)(DMF)_0.1_(H_2_O)_0.6_	226.2	12.0

The reproducibility and accuracy of the values determined by qNMR for the experimental minimal formula were confirmed through independent analysis of the same MOF sample by another researcher in the same lab. The standard deviation over three measurements of the repeat experiments was ± 0.2 for the amount of BTC linker (x), ± 0.1 for the amount of acetate modulator (y), and ± 0.05 for the amount of MeOH (z). The same value was obtained for the amount of chloride (i) over three measurements. On the other hand, based on the residual weight determined by TGA ($W_{{\mathrm{ZrO}}_{2}}$), the amount of adsorbed water was observed to increase over time, resulting in a significantly higher experimental molar mass for the hydrated MOF. However, this did not affect the values determined by qNMR or UV–vis if the experimental molar mass was determined by TGA within a few days of the measurements. Therefore, for the most accurate results in practical applications, it is important to determine the experimental minimal formula of the MOF prior to using it and to store the MOF under dry conditions to minimize water adsorption over time. The methodology for determining the experimental minimal formula of MOFs is summarized in a flowchart, see **Figure** **5**.

## 3 Generalization and Outlook

Following the flow chart in Figure 5, we have also determined the experimental minimal formulae for UiO‐66(Zr), UiO‐66(Ce), MOF‐5(Zn), MIL‐125(Ti), and MIL‐100(Fe); see Supporting Information. The experimental minimal formulae of these MOFs in comparison with their ideal minimal formulae are shown in Table 3, along with their corresponding molar masses and percentage metal by weight. Based on our analysis, UiO‐66(Zr) has missing linker defects and residual chloride from the metal precursor. In this sample, the amount of formate detected by NMR was extremely low (0.06 equivalents) and, therefore, was not included in the minimal formula. UiO‐66(Ce) was also found to have a similar number of missing linker defects. Since UiO‐66(Ce) was synthesized from (NH_4_)_2_[Ce(NO_3_)_6_], residual nitrate was found in the MOF (Figure S24, Supporting Information) instead of chloride. This again highlights the importance of taking into account all species that may become part of the MOF structure. On the other hand, MOF‐5(Zn) was determined to have the ideal composition (within the precision of the measurement), with some adsorbed water. MIL‐125(Ti) is also defect‐free (within the precision of the measurement), but was found to contain a significant amount of water and some interesting organic products such as trimethylamine (TMA), which could be formed by disproportionation of DMA (from the decomposition of DMF) to methylamine and TMA.^[^ ^50^ ^]^ As a result, MIL‐125(Ti) has a large difference in percentage metal by weight between the ideal and experimental minimal formulae (Table 3). MIL‐100(Fe) also has a structure that is free of missing linker defects (within the precision of the measurement), but a significant amount of solvent is also trapped in the pores and some of the hydroxide ligands are replaced by chloride due to the use of FeCl_3_·6H_2_O as a precursor in the synthesis of the MOF. This was confirmed by washing the MOF in acetone for several hours which caused the MeOH peak to decrease in the ^1^H NMR spectrum (Figure S52, Supporting Information) but did not significantly affect the amount of chloride determined by UV–vis (Figure S53, Supporting Information). It should be noted that the composition of MIL‐100(Fe) could be determined by qNMR despite the presence of paramagnetic Fe^3+^ in the MOF because Fe^3+^ precipitates out of solution during base digestion. Moreover, the concentration of BTC determined from base digestion was in agreement with that determined from acid digestion in D_2_SO_4_/DMSO, which formed a clear solution without precipitates but gave rise to much broader peaks due to the presence of paramagnetic Fe^3+^. Further generalization to other MOFs should be feasible, but one should consider the metal precursor used in the MOF synthesis and any other species used during the synthesis or washing steps that may become incorporated into the MOF. Depending on the metal precursors used (e.g., chlorides or nitrates), different commercial photometric tests can be used to determine the residual amount of inorganic anions.

We thus now have an easy and accessible method to assess the defects in MOFs and even test the reproducibility of MOF synthesis. Note that the experimental molar mass will depend on the temperature and the relative humidity of the environment. In addition, the experimental molar mass is relatively simple to determine for MOFs with a single metal node, but multimetallic MOFs are more complicated. Also, chemisorbed metals could confound the TGA analysis. In both cases, the strategy should involve determining the ratio of metals (and thus their oxide ratio in TGA) via other methods such as inductively coupled plasma mass spectrometry (ICP‐MS).

Interestingly, our approach does not require a crystalline material. It provides compositional information independent of the structure. The only assumption made here is the chemical composition of the node. If that requirement is satisfied, the flowchart in Figure 5 can also be applied to amorphous porous materials with the same building blocks. The experimental minimal formula provides the average composition and thus only quantifies the number of missing linkers with respect to the amount of nodes. In case of missing nodes, the experimental linker equivalence could even be higher than in the ideal formula.^[^ ^26^, ^51^ ^]^ To demonstrate the generality of this method towards coordination networks of unknown structure, we have synthesized an aluminium coordination network with benzene dicarboxylic acid. The structure is poorly crystalline and somewhat porous (see Figures S36 and S37, Supporting Information). We follow the methodology and arrive at a the minimal formula: Al(OH)(BDC)(DMF)_0.1_(H_2_O)_0.6_, see Supporting Information. The formula is reminiscent of the MIL‐53(Al) MOF.

## 4 Conclusion

We have presented a simple approach for determining the minimal formula of a MOF, based on the experimental data from TGA, qNMR, and UV–vis spectroscopy. We discussed the limitations of using TGA as the sole technique and laid out the requirements for qNMR: complete, but gentle digestion of the MOF and appropriate T_1_ relaxation delay times. A photometric test provides the chloride or nitrate content, an aspect often overlooked when establishing a MOF's minimal formula. While MOF‐808 was used here as the model example, the reasoning can be easily generalized to other MOFs, as demonstrated for UiO‐66(Zr), UiO‐66(Ce), MOF‐5(Zn), MIL‐125(Ti), MIL‐100(Fe), and an unknown Al‐BDC framework. Despite the reliance of this approach on qNMR, this method can even be used for MOFs with paramagnetic metal ions as long as the metal ions can be precipitated out of solution or the paramagnetic broadening is not too severe, as illustrated for MIL‐100(Fe). Even MOFs with linkers or modulators that contain other NMR active nuclei such as ^19^F or ^31^P could be analyzed, as long as the appropriate internal references and delay times are used. Finally, we introduced the concept of the experimental molar mass, which can deviate significantly from the idealized molar mass and it can, therefore, serve as an important tool to improve the reproducibility and accuracy of MOF applications.

## Conflict of Interest

The authors declare no conflict of interest.

## Supporting information

Supporting Information

## Data Availability

The data that support the findings of this study are openly available in Zenodo at https://doi.org/10.5281/zenodo.15013202 , reference number 15013202.
