# Supplementary material for: Determination of the Experimental Minimal Formula of Metal‐Organic Frameworks
Source: Adv Sci (Weinh). 2025 Jun 19;12(30):e04713. doi: 10.1002/advs.202504713 (PMC12376691; doi:10.1002/advs.202504713)
Supplement: Supplementary file 1 — Supporting Information [file ADVS-12-e04713-s001.pdf]

ADVANCED SCIENCE
Open Access

Supporting Information

for *Adv. Sci.*, DOI 10.1002/advs.202504713

Determination of the Experimental Minimal Formula of Metal-Organic Frameworks

*Jikson Pulparayil Mathew, Charlotte Simms, David E. Salazar Marcano, Evert Dhaene, Tatjana N. Parac-Vogt and Jonathan De Roo**

Supporting Information: Determination of the experimental minimal formula of metal-organic frameworks

Jikson Pulparayil Mathew,[†] Charlotte Simms,[‡,¶] David E. Salazar Marcano,[†] Evert Dhaene,[†] Tatjana N. Parac-Vogt,[‡] and Jonathan De Roo[*,†]

†*Department of Chemistry, University of Basel, Mattenstrasse 22, 4058 Basel, Switzerland*

‡*Department of Chemistry, KU Leuven, Celestijnenlaan 200F, 3001 Leuven, Belgium*

¶*Department of Chemistry, University of Antwerp, Universiteitsplein 1, 2610 Wilrijk, Belgium*

E-mail: Jonathan.DeRoo@unibas.ch

Contents

1 Synthetic methods **S-3**

1.1 Materials S-3

1.2 Synthesis of MOF-808(Zr) S-3

1.3 Synthesis of UiO-66(Zr) S-4

1.4 Synthesis of UiO-66(Ce) S-4

1.5 Synthesis of MOF-5(Zn) S-5

1.6 Synthesis of MIL-125(Ti) S-5

1.7 Synthesis of MIL-100(Fe) S-5

1.8 Synthesis of an Al-BDC porous coordination network S-6

2 MOF Characterization S-6

2.1 Characterization of Zr-MOFs S-7

2.2 Characterization of other MOFs S-11

3 TGA Acquisition S-14

4 Quantitative NMR analysis S-15

5 Inversion recovery experiments S-16

6 Quantification of chloride and nitrate via UV-vis S-16

6.1 Chloride quantification S-16

6.2 Nitrate quantification S-18

7 UiO-66(Zr): minimal formula S-20

7.1 TGA analysis to determine the molar mass of MOF S-20

7.2 NMR analysis to determine the concentration of organics S-22

7.3 UV-vis analysis to determine the amount of inorganic ligands S-23

7.4 Determination of hydroxide amount for charge balancing S-24

7.5 Water content in the MOF framework S-25

8 Generalization to other MOFs S-26

8.1 UiO-66(Ce): minimal formula S-27

8.1.1 TGA analysis to determine the molar mass of MOF S-27

8.1.2 NMR analysis to determine the concentration of organics S-28

8.1.3 UV-vis analysis to determine the amount of inorganic ligands S-29

8.1.4 Determination of hydroxide amount and water content S-30

8.2 MOF-5(Zn): minimal formula S-31

8.2.1 TGA analysis to determine the molar mass of MOF S-31

8.2.2 NMR analysis to determine the concentration of organics S-33

8.3 MIL-125(Ti): minimal formula S-34
8.3.1 TGA analysis to determine the molar mass of MOF S-34
8.3.2 NMR analysis to determine the concentration of organics S-36
8.4 MIL-100(Fe): minimal formula S-37
8.4.1 TGA analysis to determine the molar mass of MOF S-38
8.4.2 NMR analysis to determine the concentration of organics S-39
8.4.3 UV-vis analysis to determine the amount of inorganic ligands S-40
8.4.4 Determination of hydroxide amount for charge balancing S-41

9 Minimal formula for unknown frameworks S-42
9.1 Al-BDC MOF: minimal formula S-44
9.1.1 TGA analysis to determine the molar mass of MOF S-44
9.1.2 NMR analysis to determine the concentration of organics S-46

10 Supporting Information for Main Text S-48

References S-55

1 Synthetic methods

1.1 Materials

All chemicals used for synthesis were purchased as pure reagent grade and used without further purification.

1.2 Synthesis of MOF-808(Zr)

MOF-808 was prepared using a green route as described by Reinsch *et.al.* with slight modifications.[S1] $ZrOCl_2 \cdot 8H_2O$ (8.1 mmol, 2.6 g, 6 equivalents) and benzene-1,3,5-tricarboxylic acid (H_3BTC; a.k.a. trimesic acid) (2.7 mmol, 0.56 g, 2 equivalents) were mixed in a 100 mL

pressure-resistant glass vessel along with 20 mL of water and 20 mL of glacial acetic acid (99.7%). This was then heated at 95 °C for 24 h under static conditions in an oven. After 24 h, the white precipitate formed was collected using centrifugation and washed by adding methanol (20 mL, 4 times) over a time period of 48 h with shaking. The MOF was dried in air at 70 °C for 4 h followed by activation at 110 °C for 20 h and was stored in a desiccator.

1.3 Synthesis of UiO-66(Zr)

UiO-66 was synthesized using a solvothermal approach as described by Vermoortele *et. al.* with slight modifications.[S2] $ZrCl_4$ (15 mmol, 3.5 g, 6 equivalents) and benzene-1,4-dicarboxylic (H_2BDC; a.k.a. terephthalic acid) (15 mmol, 2.5 g, 6 equivalents) acid were mixed with 155 mL of dimethyl formamide (DMF) and 1.5 mL of hydrochloric acid (HCl) (37%) in a 1 L pressure bottle and heated to 120 °C for 24 h under static conditions in an oven. After 24 h, a white precipitate was formed and collected by centrifugation. The MOF was washed by adding acetone (20 mL, 4 times) over a time period of 48 h with shaking. The white powder was finally collected via centrifugation and dried in air at 70 °C for 4 h followed by activation at 110 °C for 20 h and stored in a desiccator.

1.4 Synthesis of UiO-66(Ce)

UiO-66(Ce) was synthesized according to the procedure by Lammert *et. al.*[S3] An aqueous solution of $(NH_4)_2[Ce(NO_3)_6]$ (0.53 M, 5 mL, 2.32 g) was mixed with H_2BDC (4.26 mmol, 0.708 g) in DMF (24 mL) in a glass reactor. The reactor was sealed and heated using an oil bath under stirring at 100 °C for 15 min. The solid collected was then washed 3 times with DMF and 2 times with EtOH. The solid was finally dried under vacuum at room temperature. The sample was then activated at 150 °C.

1.5 Synthesis of MOF-5(Zn)

MOF-5(Zn) was synthesized using the approach described by Trachemontagne *et. al.*[S4] A solution of $Zn(OAc)_2 \cdot 2H_2O$ (77.4 mmol, 16.99 g) in 500 mL DMF was added to H_2BDC (30.5 mmol, 5.065 g) and triethylamine (83.5 mmol, 8.5 mL) in 400 mL of DMF under stirring over 15 min. This formed a precipitate that was kept under stirring for another 2.5 h. The precipitate was then filtered and immersed in DMF (250 mL) overnight. It was filtered again and transferred to 350 mL of chloroform for the next 7 days with 3 times replacement of chloroform. After 7 days, the product was collected and dried under vacuum overnight. It was then activated at 120 °C under vacuum for 6 h.

1.6 Synthesis of MIL-125(Ti)

MIL-125(Ti) was synthesized based on the approach used by Dan-Hardi *et. al.*[S5] with some modifications. $Ti(O^iPr)_4$ (7.1 mmol, 2.1 mL) and H_2BDC (21.2 mmol, 3.53 g) were mixed in 54 mL of DMF and 6 mL of methanol. The reaction mixture was stirred at 25 °C for 30 min resulting in a transparent homogeneous solution. The solution was then transferred to a 100 mL Teflon-lined autoclave and was heated at 150 °C in the furnace for 16 h. After cooling down to room temperature, the powder formed was collected via centrifugation. This was then washed with 80 mL of EtOH for three times and was finally dried at 80 °C for 16 h.

1.7 Synthesis of MIL-100(Fe)

MIL-100(Fe) was synthesized according to Schertenleib *et. al.*[S6] $FeCl_3 \cdot 6H_2O$ (36 mmol, 9.72 g) and H_3BTC (16 mmol, 3.36 g) were mixed in 120 mL of distilled water. The reaction mixture was transferred to a 180 mL Teflon-lined autoclave and was heated to 130 °C for 72 h. After cooling down to room temperature, the orange powder formed was filtered under vacuum and washed with water and ethanol. The obtained powder was loaded into a double-thickness Whatman cellulose extraction thimble and underwent Soxhlet purification

with methanol for 24 h. The solid product was finally dried overnight under vacuum.

1.8 Synthesis of an Al-BDC porous coordination network

Al-BDC was synthesized using a solvothermal approach as described by Moumen *et. al.*[S7] $AlCl_3$ (2.1 mmol, 0.281 g) and H_2BDC (3.37 mmol, 0.560 g) were mixed in 30 mL of DMF. The reaction mixture was transferred to a 50 mL round bottom flask and heated at 130 °C under static conditions. After 72 h, the obtained powder was filtered and washed with DMF and acetone to remove unreacted materials in the pores. The solid was finally dried at 80 °C overnight.

2 MOF Characterization

Scanning Transmission Electron Microscopy (STEM) images, **STEM Energy-Dispersive X-ray spectroscopy (STEM-EDX)** elemental mapping, and EDX spectra were acquired with a JEOL JEM-F200 (200 kV). Samples were prepared by sonicating 1 mg of MOF or ZrO_2 in 1 mL of ethanol for 2 min and then dropcasting 10 μL onto Holey Carbon 300 mesh Cu TEM grids.

Powder X-ray Diffraction (pXRD) patterns were acquired for solid samples on low-background silica sample holders using a SmartLab Rigaku diffractometer equipped with an HyPix-3000 detector and a 9kW Cu rotating anode (45kV; 200 mA) or using a Malvern PANalytical Empyrean diffractometer with a PIXcel3D solid state detector and a Cu anode.

Fourier-Transform Infrared (FTIR) spectra were acquired by attenuated total reflectance (ATR) on a PerkinElmer Spectrum Two spectrometer with a diamond crystal.

Nitrogen Adsorption isotherms were acquired using the microtrac Belsorp Max X instrument.

2.1 Characterization of Zr-MOFs

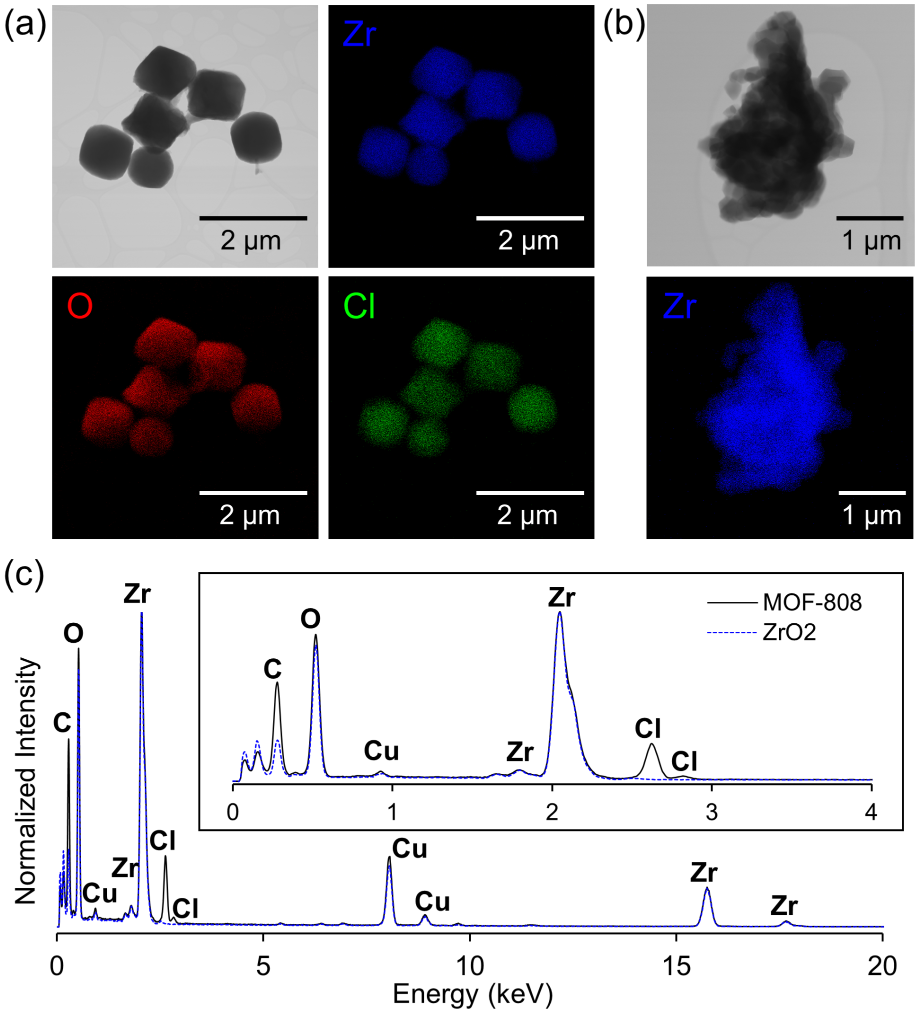

Figure S1: (a) STEM bright field image (top left) of MOF-808(Zr) particles and the corresponding EDX elemental maps for Zr, O, and Cl. (b) STEM bright field image (top right) of ZrO_2 produced from MOF-808(Zr) heated at 900 °C and the corresponding EDX elemental map for Zr. (c) EDX spectra for the same regions of the MOF-808(Zr) and ZrO_2 STEM/EDX images. Cu peaks are from the TEM grid.

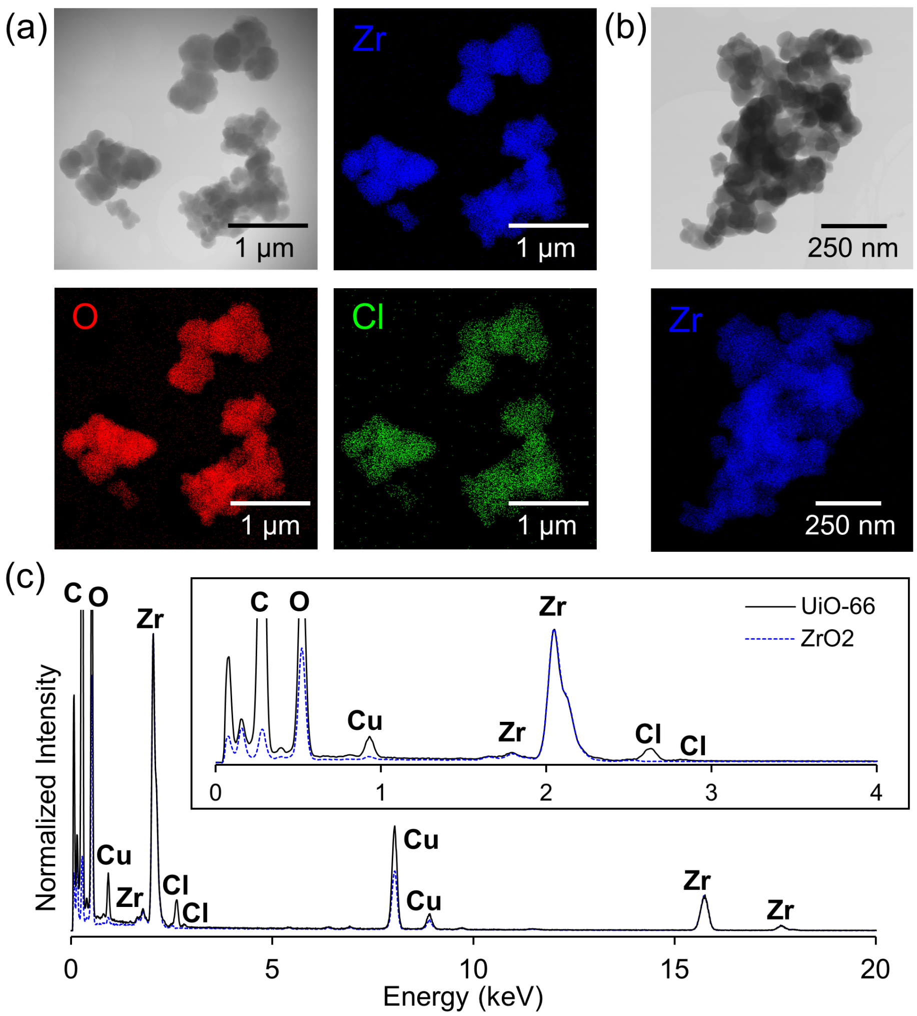

Figure S2: (a) STEM bright field image (top left) of UiO-66(Zr) particles and the corresponding EDX elemental maps for Zr, O, and Cl. (b) STEM bright field image (top right) of ZrO_2 produced from UiO-66(Zr) heated at 900 °C and the corresponding EDX elemental map for Zr. (c) EDX spectra for the same regions of the UiO-66(Zr) and ZrO_2 STEM/EDX images. Cu peaks are from the TEM grid.

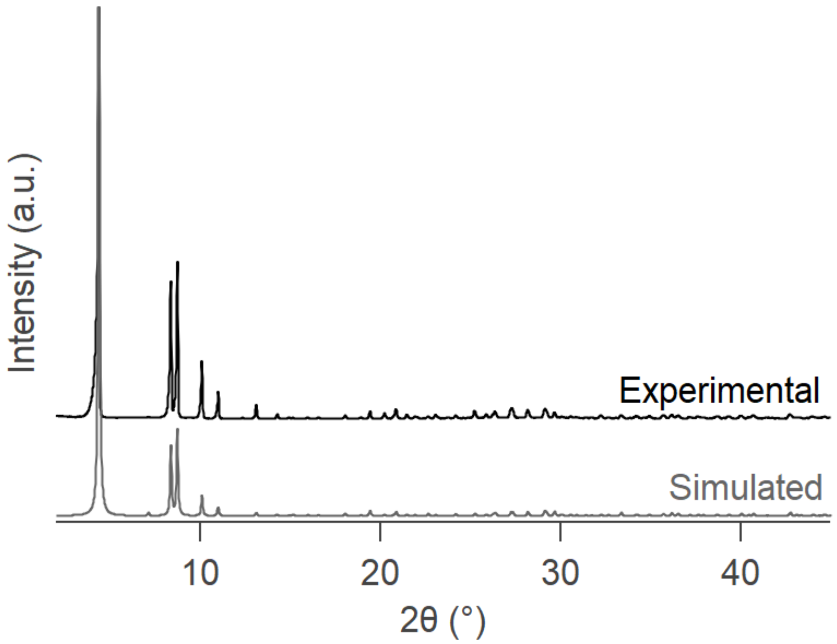

Figure S3: pXRD data for MOF-808(Zr). The simulated data is given in grey and is based on CCDC 1002672.[S8]

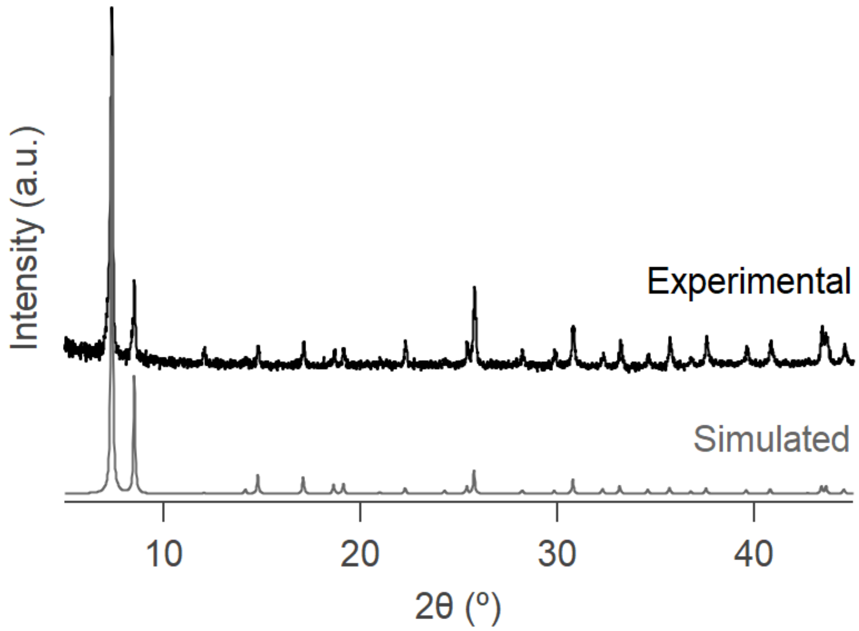

Figure S4: pXRD data for UiO-66(Zr). The simulated data is given in grey and is based on the data from CCDC 837796. The additional line at 12° is more intense than in the reference pattern as has been previously reported for solvated as-synthesized MOFs.[S9]

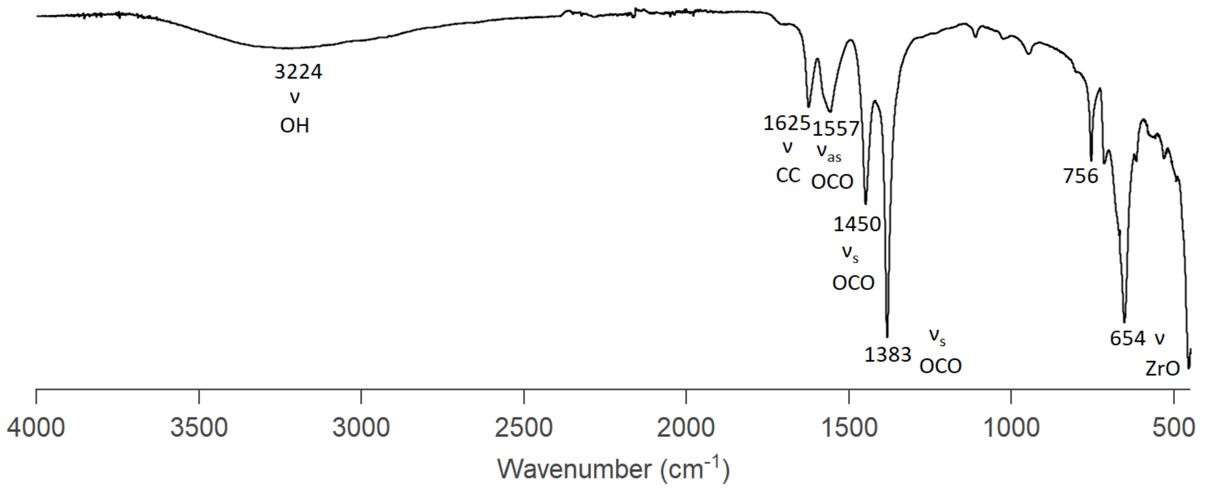

Figure S5: FTIR spectrum of MOF-808(Zr), which matches the FTIR spectra reported in the literature and can be assigned based on the computationally calculated active vibrational frequencies.[S10]

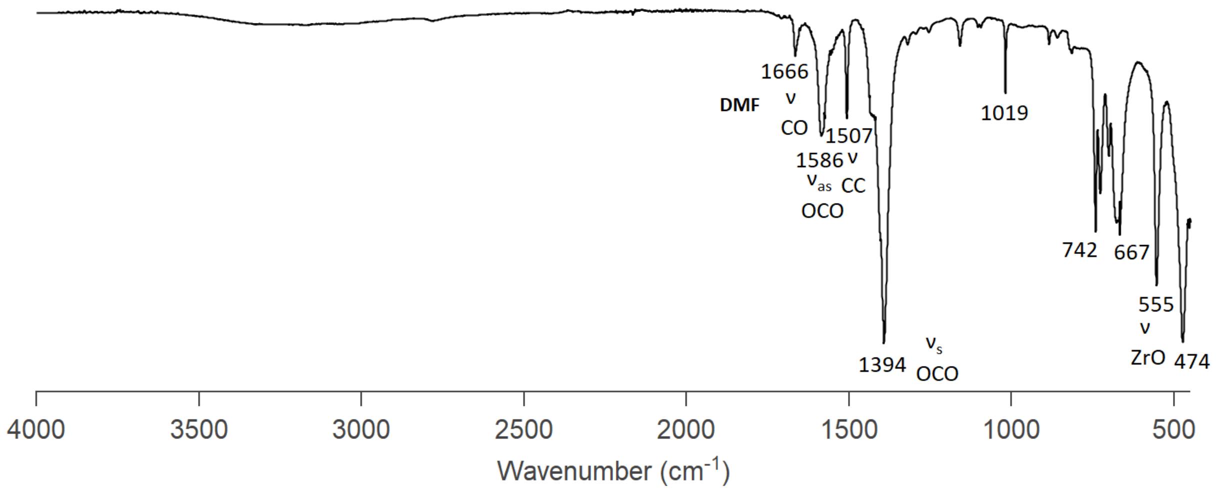

Figure S6: FTIR spectrum of UiO-66(Zr), which matches the FTIR spectra reported in the literature including a peak corresponding to adsorbed DMF.[S11,S12]

2.2 Characterization of other MOFs

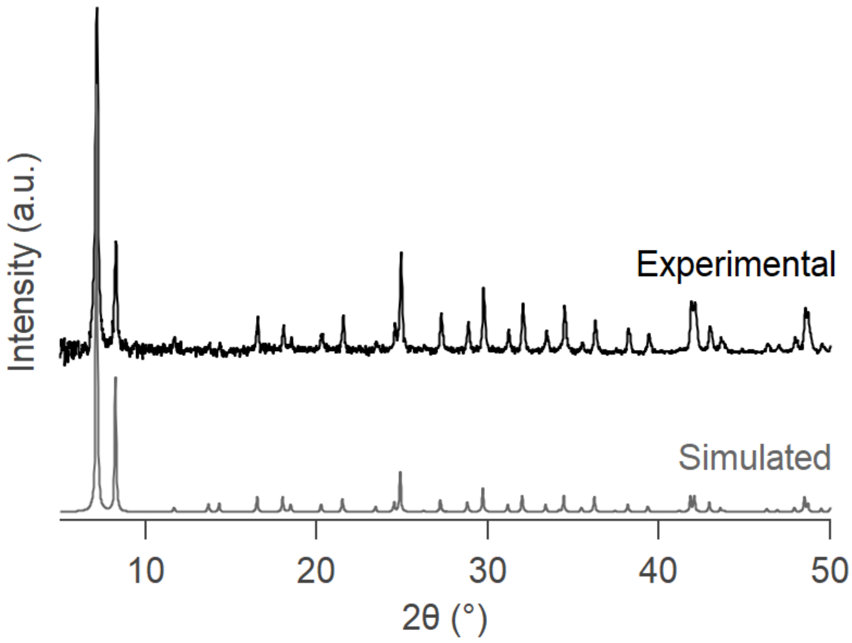

Figure S7: pXRD data for UiO-66(Ce). The simulated data is given in grey and is based on CCDC 1036904.[S3]

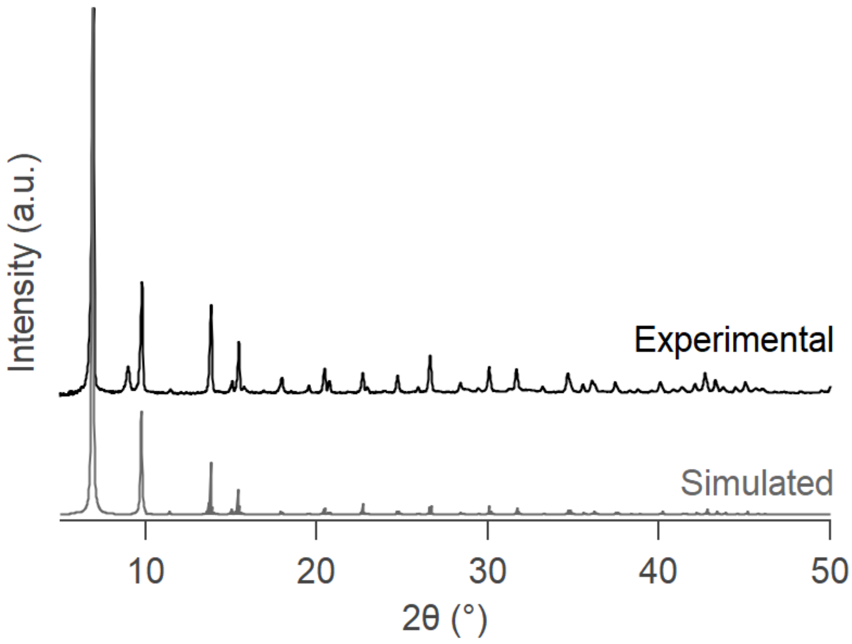

Figure S8: pXRD data for MOF-5(Zn). The simulated data is given in grey and is based on ICSD 144277.[S13]

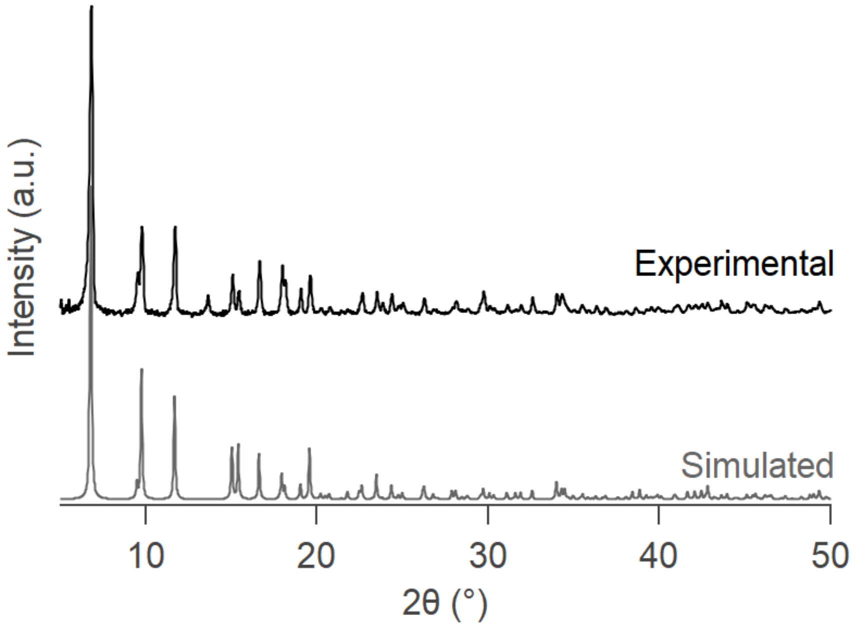

Figure S9: pXRD data for MIL-125(Ti). The simulated data is given in grey and is based on data from CCDC 751157.[S5]

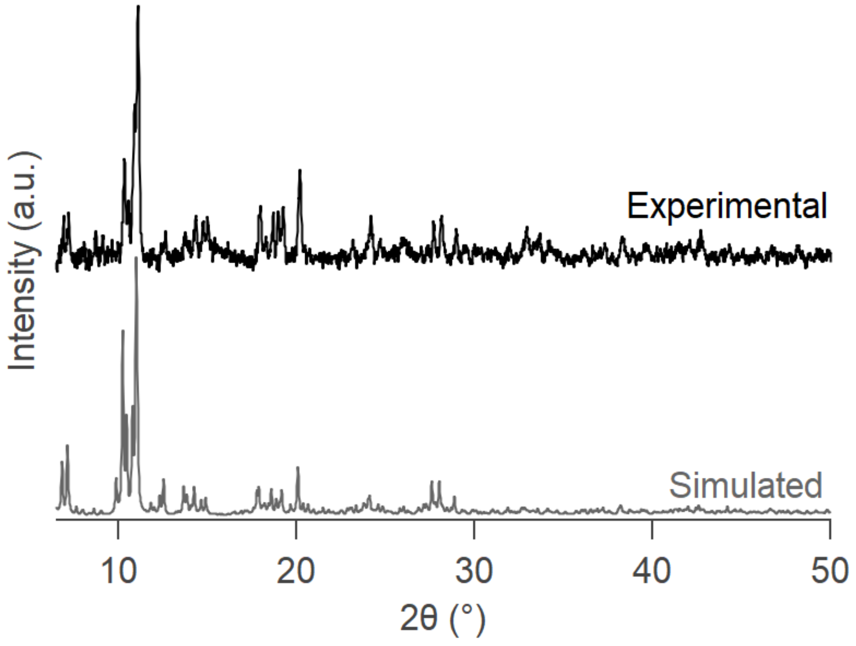

Figure S10: pXRD data for MIL-100(Fe). The simulated data is given in grey and is based on data from CCDC 640536.[S14]

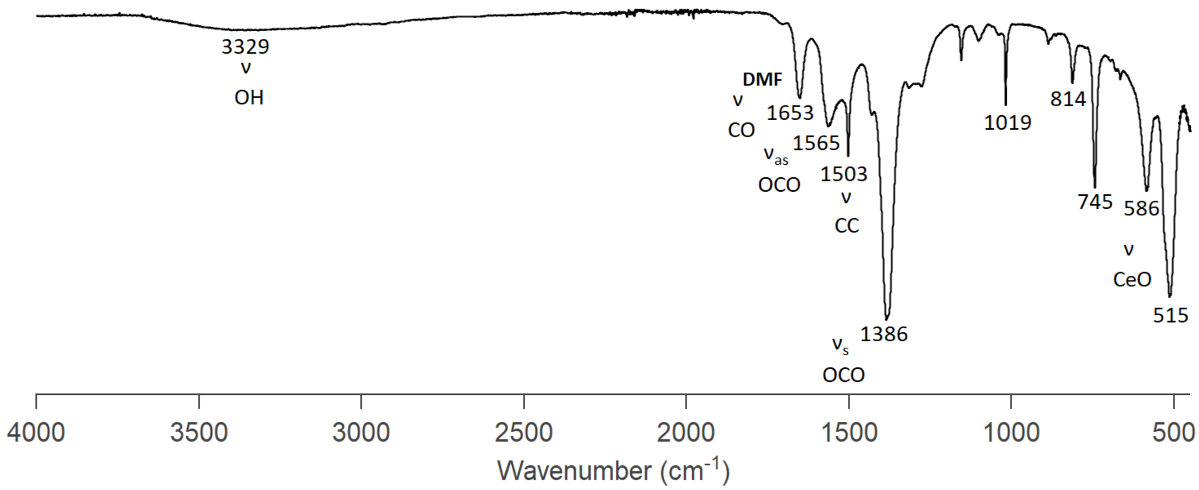

Figure S11: FTIR spectrum of UiO-66(Ce), which matches the FTIR spectra reported in the literature.[S3]

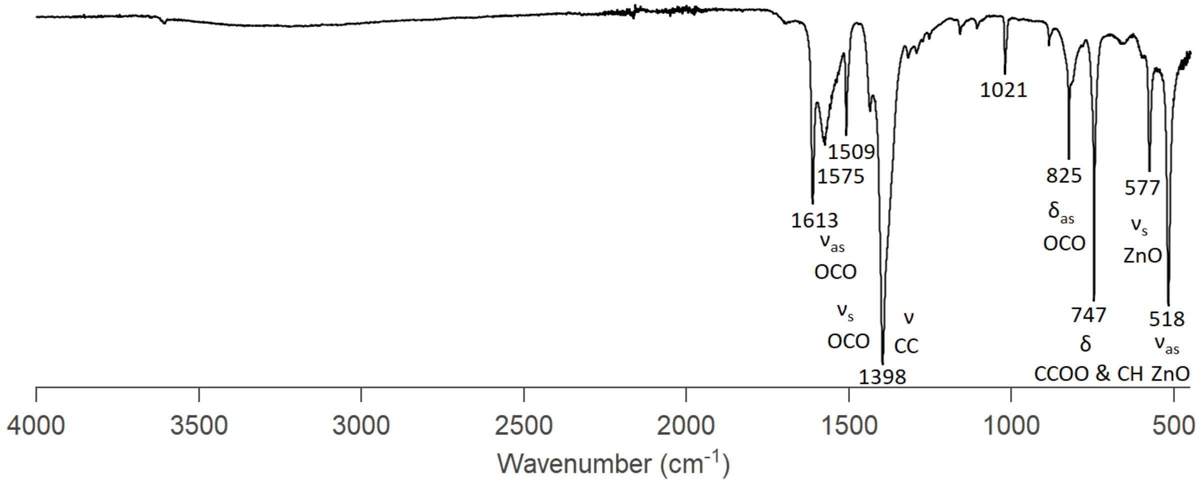

Figure S12: FTIR spectrum of MOF-5(Zn), which matches the FTIR spectra reported in the literature[S15] and can be assigned based on the computationally calculated active vibrational frequencies.[S16]

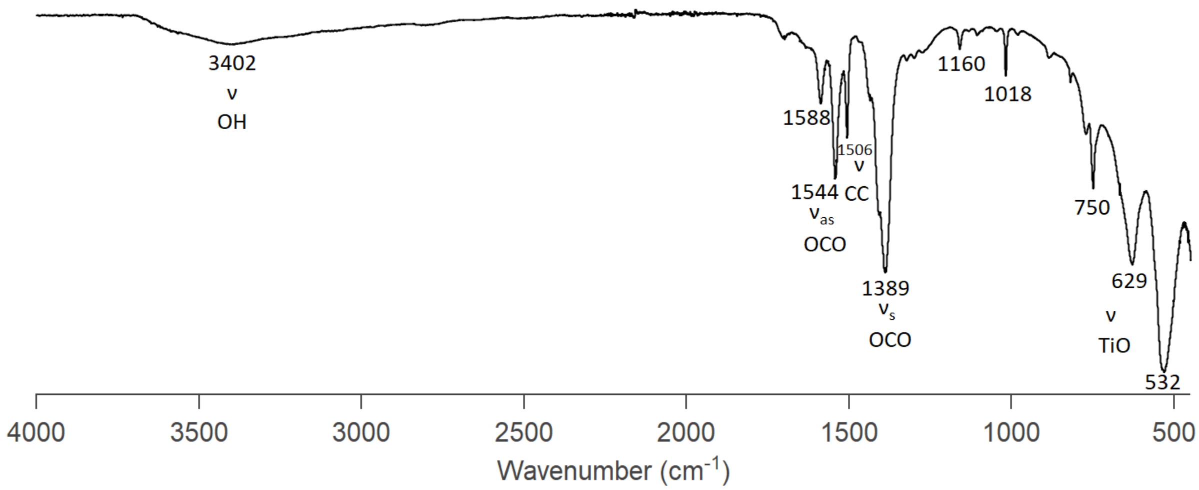

Figure S13: FTIR spectrum of MIL-125(Ti), which matches the FTIR spectra reported in the literature.[S17,S18]

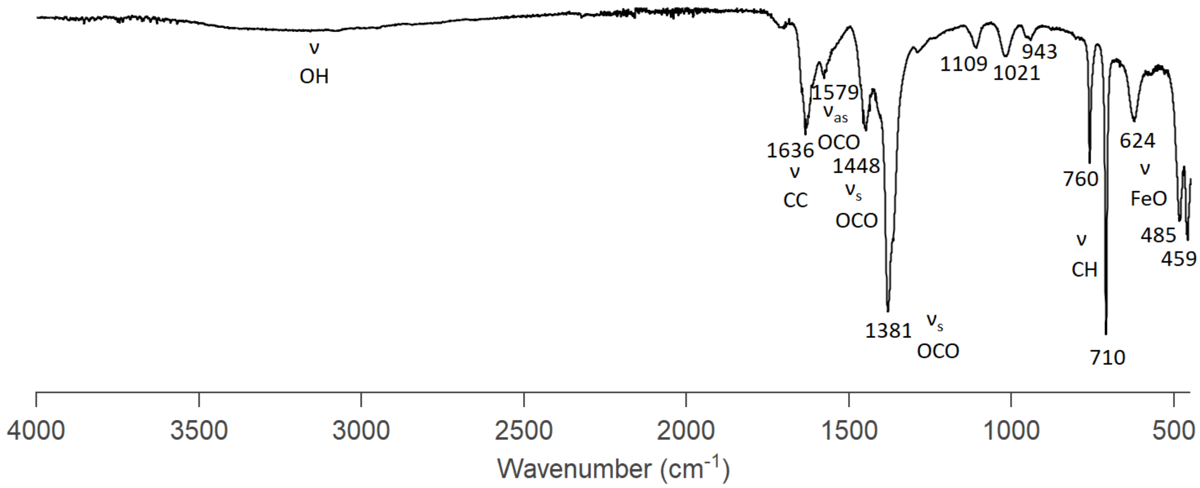

Figure S14: FTIR spectrum of MIL-100(Fe), which matches the FTIR spectra reported in the literature.[S19]

3 TGA Acquisition

The mass loss as a function of temperature was measured in the range 30 – 900 °C, under air, using a ramp rate of 2 or 5 °C/min on a Discovery TGA 5500 instrument by transferring each sample into a dry alumina crucible. A few factors affect the quality of the measurement. The crucible should be dry and heated using a Bunsen burner to remove residual volatile

compounds.[S20] Alumina is the preferred material for the crucible since it is thermally resistant up to 1200 °C. The combustion is done in air with a flow rate of 50 mL/min and a balance flow of 10 mL/min. The TGA analysis for determining the experimental molar mass is done in air to confirm the complete oxidation of the MOF to its corresponding oxide. For the TGA-MS analysis, we have attached an MKS Cirrus^{TM} 3 gas analyser, equipped with a quadrupole mass spectrometer to investigate the chemical nature of the species leaving the sample in the TGA run. While doing the MS, the combustion in TGA is done under a nitrogen atmosphere with a flow rate of 50 mL/min and a balance flow of 10 mL/min.

4 Quantitative NMR analysis

To digest a MOF, a basic solution of 1 M base (NaOH, $NaHCO_3$, or NH_4HCO_3) in D_2O was added to the MOF so that the concentration of the MOF was 1.53 mM. The amount of MOF and volume of basic solution were determined based on the experimental molar mass determined by TGA. This solution was then sonicated until a clear solution was obtained (for majority of the MOFs) in the case of bicarbonates. In the case of NaOH digestion, the solution was centrifuged to remove the precipitate. 490 μL of the clear MOF digestion solution was taken and mixed with 10 μL of 0.1 M sodium salt of 3-(trimethylsilyl)propionic-2,2,3,3-d_4 acid (TMSP-d_4) solution. This gives a final concentration of 1.5 mM for the MOF and 2 mM for TMSP-d_4. For acid digestion, dimethyl sulfone (0.109 mmol, 10.3 mg) was dissolved in 7 mL DMSO-d_6. 0.7 mL from this was added to 1.0717 mmol of MOF sample along with a few drops of D_2SO_4 and was subjected to sonication. The solution was then used for quantitative ^{1}H NMR measurements at 298 K on a Bruker Ascend^{TM}500 MHz NMR spectrometer. For a quantitative NMR experiment with a 90 degree pulse, the relaxation delay time (D1, an acquisition parameter) needs to be set according to the longest T_1 value of the anticipated species in the sample ($5\times T_1$, see Table 2). For a quantitative NMR experiment with a 30 degree pulse, D1 should be $3\times T_1$. Unless otherwise mentioned, all our

NMR experiments were quantitative.

5 Inversion recovery experiments

The linker molecule was dissolved in 1M NH_4HCO_3 in D_2O solution so that the final concentration of the linker molecules was always 50 mM or less. This is then homogenized for proper dissolution and analysed with NMR. The inversion recovery experiments, were performed using the t1ir pulse program from the Bruker database at 298 K on a Bruker Ascend^{TM}600 MHz NMR spectrometer. The T_1 was then determined using the relaxation module in Topspin.

Alternatively, an estimation of the D1 delay time can be obtained by performing multiple ^{1}H NMR experiments with increasing delay times until the concentration of the analyte stops increasing.

6 Quantification of chloride and nitrate via UV-vis

6.1 Chloride quantification

To quantify the amount of chloride ions present, the Spectroquant chloride test protocol (1.14897.0001) for the chloride concentration range of 10 to 250 mg/L was used. With this test, chloride ions react with mercury(II) thiocyanate to form mercury(II) chloride. The thiocyanate released in the process in turn reacts with iron(III) ions to form red iron(III) thiocyanate, which can be quantified photometrically.

MOFs were digested in 1 M aqueous ammonium bicarbonate (around 5 mg of MOF in 2 mL of basic solution or using the same solution as for qNMR). After sonication overnight to digest the MOF, 1 mL was transferred to a separate vial to which Reagent I (2.5 mL) and Reagent II (0.50 mL) were added in succession. The solution was mixed for around 1 min before being transferred to a 10 mm cuvette, which was used to measure the UV-vis

absorption on a PerkinElmer Lambda 365 spectrometer. If precipitation of the protonated carboxylate linker was observed, the mixture was centrifuged to recover the supernatant for measurements. A blank sample was also prepared in the same way by adding Reagent I (2.5 mL) and Reagent II (0.50 mL) to 1 mL of ammonium bicarbonate (1 M) without any MOF, which was used as a baseline for the measurements. The pH of the solution was measured to make sure that it was approximately 1 (the pH of the solution can be adjusted with a few drops of conc. HNO_3 or NH_3 solution as long as any impact on the concentration is taken into account; pH measurements can introduce additional Cl ions, so ideally separate solutions should be used for pH and UV-vis measurements or the pH can be estimated by adding a small drop of solution onto pH indicator paper/strips). To calculate the chloride concentration ([Cl]), the absorption at 500 nm (A_{500}) was used: [Cl] = 107 × A_{500} (in mg/L) with a method detection limit of ± 10 mg/L.

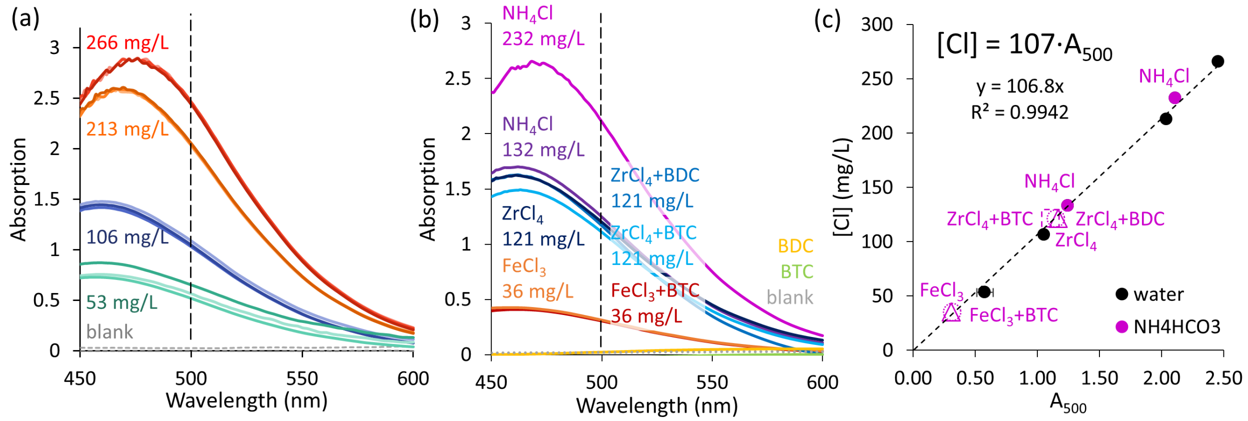

Figure S15: (a) UV-vis spectra of four NH_4Cl solutions ([Cl] = 266, 213, 106, 53 mg/L) in ultrapure water measured in triplicate. (b) UV-vis spectra of two NH_4Cl solutions ([Cl] = 232, 133 mg/L), three $ZrCl_4$ solutions ([Cl] = 121 mg/L) alone and with BDC (6.7 mM) or BTC (6.8 mM), two $FeCl_3 \cdot 6H_2O$ solutions ([Cl] = 36 mg/L) alone and with BTC (2.1 mM), and of the linkers alone (7 mM) in 1 M NH_4HCO_3. (c) Calibration curve showing the linear relationship between the Cl concentration and the absorption at 500 nm (A_{500}). Error bars correspond to the standard deviation from three repetitions (for some data points they are smaller than the marker). Data points for solutions of $ZrCl_4$ alone (dashed circle) and with BDC (triangle) or BTC (dashed square) and of $FeCl_3 \cdot 6H_2O$ alone (dashed circle) and with BTC (triangle) in 1 M NH_4HCO_3 are also shown on the calibration curve.

To validate this commercial Cl quantification test, ammonium chloride solutions of differ-

ent concentrations (7.5, 6, 3, 1.5 mM) were prepared by serial dilution in ultrapure MQ water to achieve Cl concentrations of 266, 213, 106, and 53 mg/L. Reagent I and reagent II were then added sequentially to 1 mL of each solution as described above (repeated three times for each Cl concentration). The UV-vis spectra were then acquired (Figure S15(a)). There is a clear linear relationship between the Cl concentration and the absorption at 500 nm (A_{500}) that matches the one provided by the supplier: [Cl] = 107 × A_{500} (Figure S15(c)). In addition, this relationship still holds for ammonium chloride solutions in 1 M aqueous ammonium bicarbonate, which was used for MOF digestion (Figure S15(b)). This indicates a tolerance towards variations in pH, since the pH of the solutions in water after addition of reagents I and II was 0.7 while the pH of the solutions in ammonium bicarbonate after addition of the reagents was 1.8. Furthermore, the presence of Zr^{4+} ions, Fe^{3+} ions, and/or free linker (BDC or BTC) do not significantly affect the measured absorption at 500 nm (Figure S15(b)). Overall, this demonstrates the robustness of this Cl quantification test and its suitability for determining the Cl content in MOF digestion solutions. However, potential matrix interference effects should be checked for MOFs with other metal ions or linkers.

6.2 Nitrate quantification

To quantify the amount of nitrate ions present, the Spectroquant nitrate test protocol (1.14764.0001) for the nitrate concentration range of 4 to 221 mg/L was used. With this test, nitrate ions react with 2,6-dimethylphenol in a solution of sulfuric and phosphoric acid to form 4-nitro-2,6-dimethylphenol, which can be quantified photometrically.

The MOF was digested in 1 M aqueous ammonium bicarbonate (same solution as for qNMR). The MOF digestion solution was diluted 2-fold with ultrapure water and 0.5 mL of the diluted MOF digestion solution was transferred to a reaction cell with a sulfuric and phosphoric acid solution followed by 1 mL of Reagent NO3-1K. The solution was mixed and allowed to react for 10-30 min before transferring some of the solution to a 10 mm cuvette, which was used to measure the UV-Vis absorption on a PerkinElmer Lambda 365

spectrometer. A blank sample was also prepared in the same way by 2-fold dilution of ammonium bicarbonate (1 M) without any MOF in ultrapure water and adding 0.5 mL of this diluted solution to a reaction cell followed by Reagent NO3-1K. The blank was then used as a baseline for the measurements. To calculate the concentration of nitrate ions ($[NO_3]$), the absorption at 340 nm (A_{340}) was used: $[NO_3] = 167 \times A_{340}$ (in mg/L) with a detection limit of $\pm$ 1.5 mg/L.

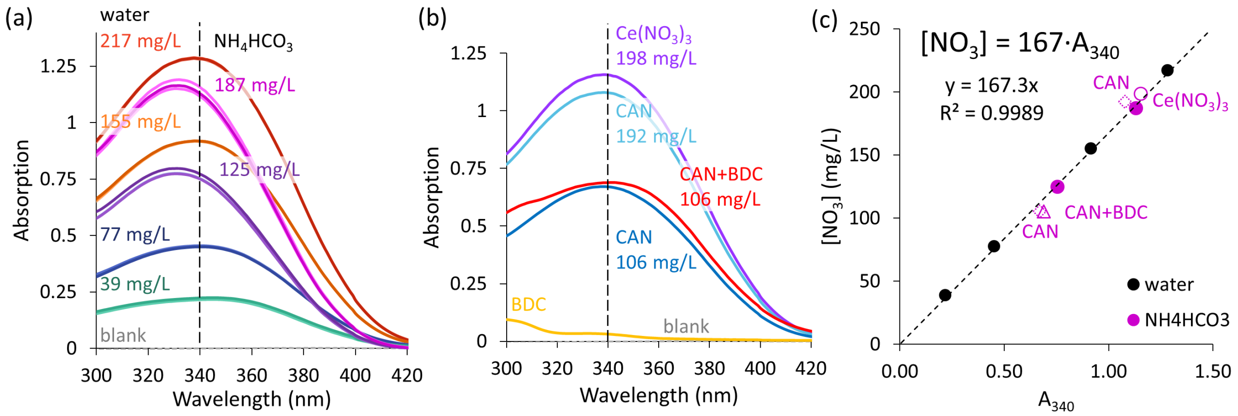

Figure S16: (a) UV-vis spectra of four $Ce(NO_3)_3 \cdot 6H_2O$ solutions ($[NO_3]$ = 217, 155, 77, 39 mg/L) in ultrapure water and of two $Ce(NO_3)_3 \cdot 6H_2O$ solutions ($[NO_3]$ = 187, 125 mg/L) in 1 M NH_4HCO_3 (measured in triplicate). (b) UV-vis spectra of solutions of $Ce(NO_3)_3 \cdot 6H_2O$ ($[NO_3]$ = 198 mg/L), $(NH_4)_2[Ce(NO_3)_6]$ ($[NO_3]$ = 192, 106 mg/L) alone and with BDC (3.6 mM), and of BDC alone (3.6 mM) in 1 M NH_4HCO_3. (c) Calibration curve showing the linear relationship between the NO_3^- concentration and the absorption at 340 nm (A_{340}). Error bars correspond to the standard deviation from three repetitions (for most data points they are smaller than the marker). Data points for solutions of $Ce(NO_3)_3 \cdot 6H_2O$ (circle) and $(NH_4)_2[Ce(NO_3)_6]$ (dashed diamond) alone and with BDC (triangle) in 1 M NH_4HCO_3 are also shown on the calibration curve.

To validate this commercial NO_3^- quantification test, cerium nitrate hexahydrate solutions of different concentrations (1.2, 0.8, 0.4, 0.2 mM) were prepared by serial dilution in ultrapure MQ water to achieve NO_3^- concentrations of 217, 155, 77 and 39 mg/L. 0.5 mL of each solution were added to a reaction cell followed by 1 mL of Reagent NO3-1K as described above. The UV-vis spectra were then acquired (Figure S16(a)) three times for each NO_3^- concentration. A clear linear relationship between the NO_3^- concentration and the absorption at 340 nm can be seen (A_{340}), which matches that provided by the supplier: $[NO_3] =$

167 × A_{340} (Figure S16(c)). In addition, like for the Cl quantificantion, this relationship still holds for cerium nitrate and cerium ammonium nitrate (CAN) solutions in 1 M aqueous ammonium bicarbonate, which was used for MOF digestion (Figure S16). Moreover, the presence of Ce^{4+} ions and/or free linker (BDC) do not significantly affect the measured absorption at 340 nm (Figure S15(b)). Overall, this commercial test is sufficiently robust and suitable for quantification of the NO_3^- content in MOF digestion solutions. However, potential matrix interference effects should be checked for MOFs with other metal ions or linkers.

7 UiO-66(Zr): minimal formula

Here we demonstrate the minimal formula derivation for UiO-66(Zr) using the methodology described in the main text. Based on a qualitative analysis, we start with the general formula: $Zr_6O_4(\mu_3{-}OH)_4(OH)_w(BDC)_x(DMF)_y(DMA)_z(Cl)_i(H_2O)_n$, where BDC is the benzenedicarboxylate linker and DMA is dimethylamine likely formed by decomposition of DMF, the solvent used for the synthesis of UiO-66(Zr).

7.1 TGA analysis to determine the molar mass of MOF

As the first step, we focus on determining the molar mass of our UiO-66(Zr) sample using TGA analysis (Figure 2a).

The ideal structure of UiO-66(Zr), without defects (i.e., fully coordinated by linkers) has the ideal minimal formula $Zr_6O_4(OH)_4(BDC)_6$ and a molar mass of 1664.1 g/mol. The cluster node is twelve-connected in the MOF structure, but each BDC linker is shared by two nodes, hence the stoichiometry in the minimal formula.

The complete combustion of an idealized UiO-66 is:

$$Zr_6O_4(OH)_4(BDC)_6 + 45O_2 \xrightarrow{\Delta} 6ZrO_2 + 48CO_2 + 14H_2O \quad (1)$$

The experimental molar mass of UiO-66(Zr) at room temperature (M_{exp}) can be calculated from the percentage residual inorganic weight formed after complete combustion. The residual inorganic solid corresponds to 6 molar equivalents of ZrO_2 ($M_{ZrO_2} = 123.2$ g/mol) based on the combustion of the idealized minimal formula and confirmed experimentally by pXRD and STEM/EDX (Figure S2 and S17). Therefore, M_{exp} can be obtained from the percentage residual weight of ZrO_2 (W_{ZrO_2}) using equation 2:

$$M_{exp} = \frac{100\%}{W_{ZrO_2}} \times 6M_{ZrO_2} = \frac{100\%}{39.9\%} \times 739.3 \tag{2}$$

Thus the experimentally determined experimental molar mass of UiO-66(Zr) is 1852.9 g/mol based on a residual weight of 39.9% (Figure 2(a)), which is significantly higher than the molar mass from the ideal minimal formula (1664.1 g/mol). This significant difference is in part due to the adsorbed water in the MOF.

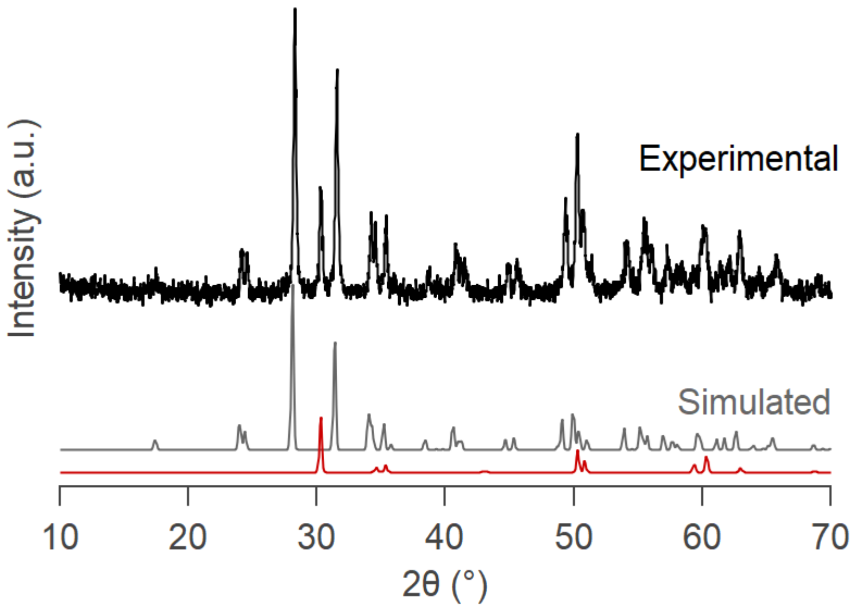

Figure S17: pXRD data confirming the formation of just ZrO_2 at the end of TGA analysis of UiO-66(Zr). The experimental data matches the simulated data based on ICSD 26488 (P121/c1 monoclinic ZrO_2)[S21] and ICSD 68781 (P42/nmcZ tetragonal ZrO_2),[S22] which is given in grey and maroon respectively.

7.2 NMR analysis to determine the concentration of organics

A solution containing 1.5 mM UiO-66(Zr) (based on the experimental molar mass) and 2 mM TMSP-d_4 was prepared from the MOF digestion solution for quantitative NMR analysis (Figure S18).

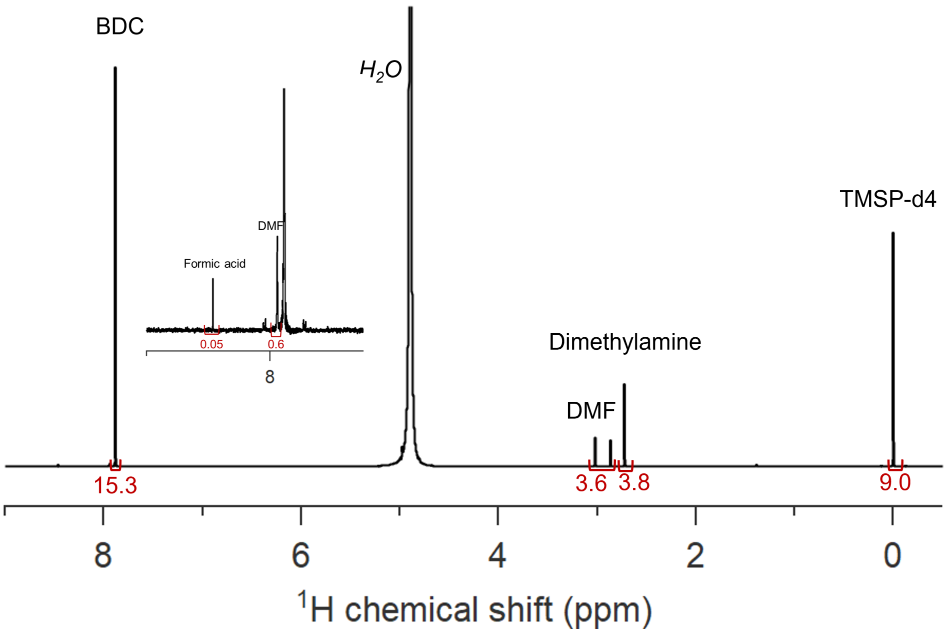

Figure S18: ^{1}H NMR spectrum in D_2O of 1.5 mM UiO-66(Zr) digested using 1 M NH_4HCO_3, with 2 mM TMSP-d_4 as internal standard. The inset shows the presence of weak peaks corresponding to formic acid and the aldehyde proton of DMF as confirmed by its matching integration with the methyl protons of DMF. The integration of the formate group gives a number of equivalents lower than 0.1 (0.06), which we deem insignificant.

The concentration of each organic species present in the MOF ([analyte]) can be determined using the following equation:

$$[analyte] = \frac{\frac{I_{analyte}}{n_{analyte}}}{\frac{I_{\mathrm{TMSP-d_4}}}{9}} \times [\mathrm{TMSP-d_4}] \qquad (3)$$

where $I_{analyte}$ denotes the integral and $n_{analyte}$ represents the number of protons corresponding to the integral of the organic analyte. Using equation 3, we find a BDC concentration of 7.6 mM, DMF concentration of 1.2 mM, and dimethylamine (DMA) concentration of 1.3 mM. Note that the DMF gives rise to 2 peaks of equal integral values (each corre-

sponding to three protons) due to slow rotation around the C-N bond resulting in the CH_3 groups being in different chemical environments. Using the MOF concentration of 1.5 mM in the NMR tube, the amount of BDC (x), DMF (y), and DMA (z) in the minimal formula, $(Zr_6O_4(\mu_3{-}OH)_4(OH)_w(BDC)_x(DMF)_y(DMA)_z(Cl)_i(H_2O)_n)$, can be easily calculated:

$$\begin{aligned} x &= \frac{[BDC]}{[UiO-66]} = \frac{7.6}{1.5} = 5.1 \\ y &= \frac{[DMF]}{[UiO-66]} = \frac{1.2}{1.5} = 0.8 \\ z &= \frac{[DMA]}{[UiO-66]} = \frac{1.3}{1.5} = 0.9 \end{aligned} \tag{4}$$

7.3 UV-vis analysis to determine the amount of inorganic ligands

Now to determine the amount of chloride in the sample we turned to UV-vis spectroscopy. A UiO-66(Zr) solution of 1.3 mM concentration shows the presence of 3.5 mM chloride based on the absorption at 500 nm.

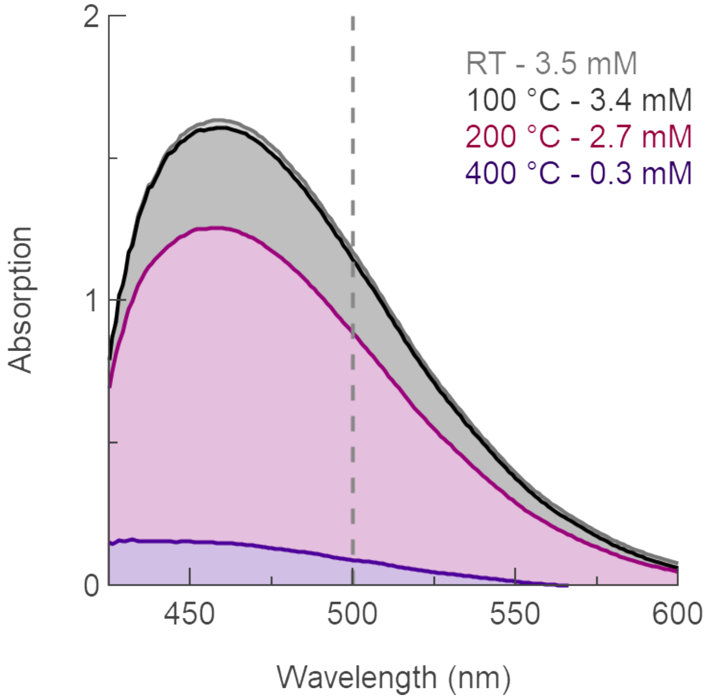

Figure S19: Chloride analysis of UiO-66(Zr) heated to various temperatures.

From this concentration, we can then calculate the i variable in the minimal formula as

$$i = \frac{[Cl]}{[UiO-66]} = \frac{3.5}{1.3} = 2.7 \tag{5}$$

From the above analysis, we arrive at the minimal formula:

$Zr_6O_4(\mu_3{-}OH)_4(OH)_w(BDC)_{5.1}(DMF)_{0.8}(DMA)_{0.9}(Cl)_{2.7}(H_2O)_n$

7.4 Determination of hydroxide amount for charge balancing

As done in the main text, the amount of hydroxides in the minimal formula can be adjusted to ensure that the 6 Zr^{4+} cations in the cluster are charge balanced by the sum of all the anionic species:

$$\begin{aligned} C^{Zr^{4+}} &= C^{O^{2-}} + C^{OH^{-}} + C^{BDC^{2-}} + C^{Cl^{-}} \\ 6 \times 4 &= 4 \times 2 + 4 + w + 2x + i \\ 24 &= 12 + w + (2 \times 5.1) + 2.7 \\ w &= -0.9 \end{aligned} \tag{6}$$

This is an unexpected result since a decrease in the amount of hydroxides would destabilize the cluster core. Considering the highly acidic reaction conditions, we propose that dimethylamine is present in the MOF pores as dimethylammonium chloride salt. Therefore, the minimal formula becomes: $Zr_6O_4(\mu_3{-}OH)_4(OH)_w(BDC)_{5.1}(DMF)_{0.8}(Cl)_{1.8}(H_2O)_n(H_2NMe_2Cl)_{0.9}$. If one now repeats the analysis in Equation 6, one arrives at:

$$
\begin{aligned}
C^{Zr^{4+}} &= C^{O^{2-}} + C^{OH^-} + C^{BDC^{2-}} + C^{Cl^-} \\
6 \times 4 &= 4 \times 2 + 4 + w + 2x + i \\
24 &= 12 + w + (2 \times 5.1) + 1.8 \\
w &= 0
\end{aligned} \tag{7}
$$

which is a quite neat result: $Zr_6O_4(OH)_4(BDC)_{5.1}(DMF)_{0.8}(Cl)_{1.8}(H_2O)_n(H_2NMe_2Cl)_{0.9}$.

7.5 Water content in the MOF framework

Disregarding water from the minimal formula, we get a dry molar mass of 1712.0 g/mol. The total amount of adsorbed water can be calculated from the difference between the experimental molar mass ($M_{exp} = 1852.9$ g/mol) and the molar mass of the anhydrous MOF determined so far ($M_{anhy} = 1712.04$ g/mol), taking into account the molar mass of water (18 g/mol).

$$
n = \frac{M_{exp} - M_{anhy}}{18\ g/mol} = 7.8 \tag{8}
$$

Therefore, using equation 8, the amount of water in the MOF was found to be 7.8 per formula unit. Thus, the final experimental minimal formula for our UiO-66(Zr) is:

$Zr_6O_4(OH)_4(BDC)_{5.1}(DMF)_{0.8}(Cl)_{1.8}(H_2O)_{7.8}(H_2NMe_2Cl)_{0.9}$. This includes coordinated water molecules to satisfy the coordination number of Zr in the Zr_6 cluster nodes of the MOF. TGA-MS analysis of UiO-66(Zr) proves that only water is adsorbed in the pores and not other molecules from the air, such as CO_2 (Figure S20).

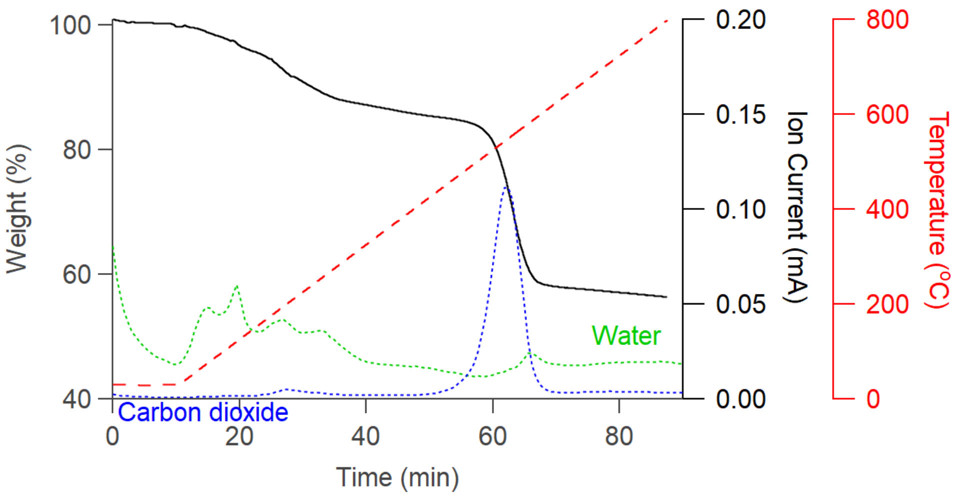

Figure S20: TGA-MS analysis of UiO-66(Zr). The black line corresponds to the sample combustion with increasing temperature (red line) under N_2 atmosphere. The ionic current corresponding to 18 amu (water; green line) and 44 amu (carbon dioxide; blue line) is plotted with respect to time and temperature.

Independent analysis by a separate researcher in the same lab confirmed the reproducibility and accuracy of the values determined by qNMR for the experimental minimal formula of UiO-66(Zr). The standard deviation of the repeat experiments was ± 0.3 for the amount of BDC linker (x) and ± 0.1 for the amount of DMF (y). The same values were obtained for the amount of H_2NMe_2Cl (z) and chloride (i). However, like for MOF-808, the residual weight determined by TGA (W_{ZrO_2}) was observed to decrease, corresponding to an increase in the amount of adsorbed water over time and resulting in a significantly higher experimental molar mass for the hydrated MOF. Nevertheless, this did not affect the values determined by qNMR or UV-vis if the experimental molar mass was determined by TGA for the sample within a few days of the measurements.

8 Generalization to other MOFs

To prove the generality of the proposed methodology, we expanded our analysis to Ce, Zn, Ti, and Fe-based MOFs. We focus on UiO-66(Ce), MOF-5(Zn), MIL-125(Ti), and MIL-100(Fe) for this purpose.

8.1 UiO-66(Ce): minimal formula

The minimal formula of an ideal UiO-66(Ce) is $Ce_6O_4(\mu_3{-}OH)_4(BDC)_6$, which has a molar mass of 1957.4 g/mol. Therefore, to determine an experimental minimal formula, we start with the general formula $Ce_6O_4(\mu_3{-}OH)_4(OH)_w(BDC)_x(NO_3)_i(DMF)_y(EtOH)_z(H_2O)_n$, where BDC is the benzenedicarboxylate linker, NO_3^- ions could be present from the $(NH_4)_2[Ce(NO_3)_6]$ MOF synthesis precursor, DMF is the solvent used for the synthesis, and EtOH was used to wash the MOF.

8.1.1 TGA analysis to determine the molar mass of MOF

The complete combustion of an idealized UiO-66(Ce) is:

$$Ce_6O_4(OH)_4(BDC)_6 + 45O_2 \xrightarrow{\Delta} 6\,CeO_2 + 48CO_2 + 14H_2O \tag{9}$$

The formation of CeO_2 as the residual solid after heating up to 900 °C was confirmed by pXRD (Figure S21).

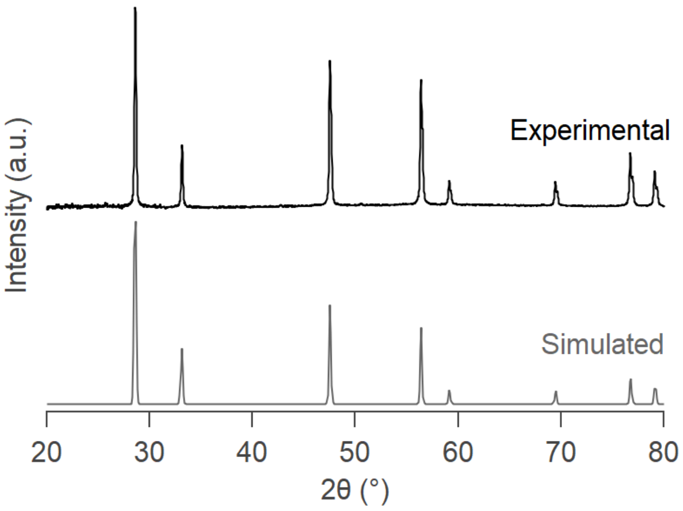

Figure S21: pXRD data confirming the formation of CeO_2 at the end of TGA. The simulated data is given in grey and is based on the data from ICSD 118604.[S23]

The experimental molar mass of UiO-66(Ce) at room temperature (M_{exp}) can be calcu-

lated from the percentage residual inorganic weight of CeO_2 formed after complete combustion (W_{CeO_2}), which corresponds to 6 molar equivalents of CeO_2 ($M_{CeO_2} = 172.11$ g/mol). This can be done using equation 10:

$$M_{exp} = \frac{100\%}{W_{CeO_2}} \times 6M_{CeO_2} = \frac{100\%}{45.2\%} \times 1032.7 \tag{10}$$

Thus, the experimentally determined molar mass of UiO-66(Ce) is 2284.7 g/mol based on a residual weight of 45.2% (Figure S22), which is significantly higher than the molar mass from the ideal minimal formula (1957.4 g/mol).

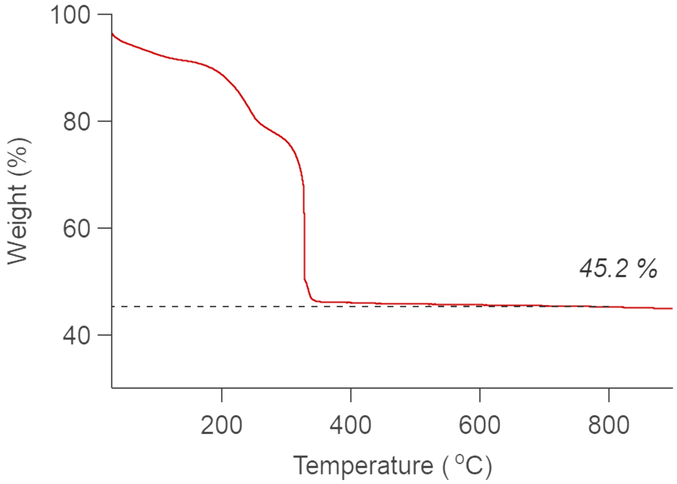

Figure S22: TGA analysis of UiO-66(Ce).

8.1.2 NMR analysis to determine the concentration of organics

A solution containing 1.6 mM UiO-66(Ce) (based on the experimental molar mass) and 2 mM TMSP-d_4 was prepared from the MOF digestion solution for quantitative NMR analysis (Figure S23).

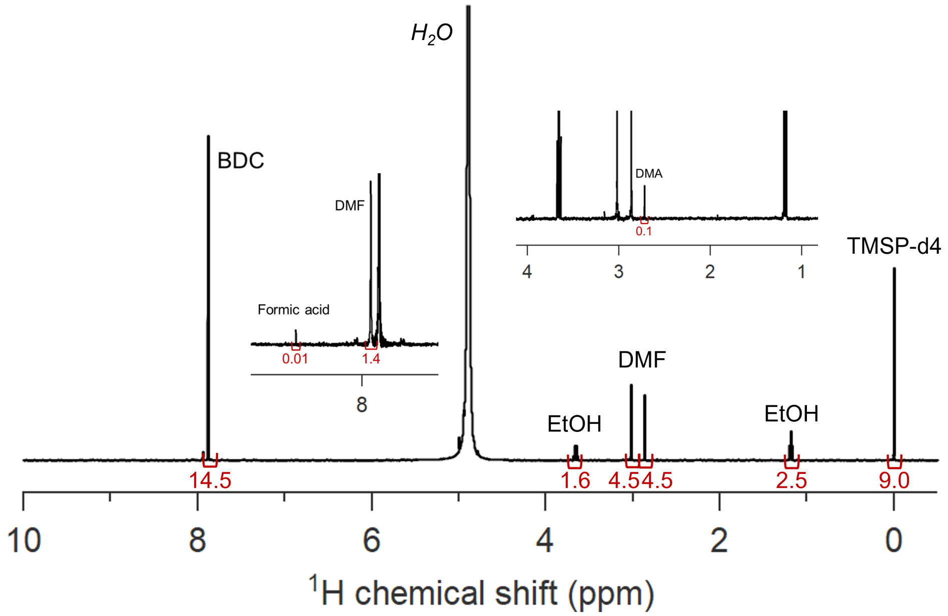

Figure S23: ^{1}H NMR spectrum in D_2O of 1.6 mM UiO-66(Ce) digested using 1 M NH_4HCO_3, with 2 mM TMSP-d_4 as internal standard. The insets show the presence of weak peaks corresponding to DMA, formic acid, and the aldehyde proton of DMF as confirmed by its matching integration with the methyl protons of DMF. The integration of the formate and DMA peaks give a number of equivalents lower than 0.1 (0.01 and 0.02, respectively), which is negligible for the minimal formula.

Using equation 3, we find a BDC concentration of 7.2 mM, DMF concentration of 3 mM, and EtOH concentration of 1.7 mM. Using the MOF concentration of 1.6 mM in the NMR tube, the amount of BDC (x), DMF (y) and EtOH (z) in the minimal formula $(Ce_6O_4(\mu_3{-}OH)_4(OH)_w(BDC)_x(NO_3)_i(DMF)_y(EtOH)_z(H_2O)_n)$ can be easily calculated:

$$\begin{aligned} x &= \frac{[BDC]}{[UiO-66(Ce)]} = \frac{7.2}{1.6} = 4.5 \\ y &= \frac{[DMF]}{[UiO-66(Ce)]} = \frac{3.0}{1.6} = 1.9 \\ z &= \frac{[EtOH]}{[UiO-66(Ce)]} = \frac{1.7}{1.6} = 1.1 \end{aligned} \tag{11}$$

8.1.3 UV-vis analysis to determine the amount of inorganic ligands

While UiO-66(Zr) contains chloride from the synthesis, UiO-66(Ce) was synthesized from $(NH_4)_2[Ce(NO_3)_6]$ and no chloride-containing species were used. So, UiO-66(Ce) may contain

nitrate ions instead of chloride. Like for the chloride content, the amount of nitrate present in the MOF can be determined photometrically using a commercial test kit as described above. In this way, the concentration of nitrate in a 2-fold diluted 1.6 mM digestion solution of UiO-66(Ce) (final concentration of 0.8 mM) was found to be 1.7 mM (Figure S24).

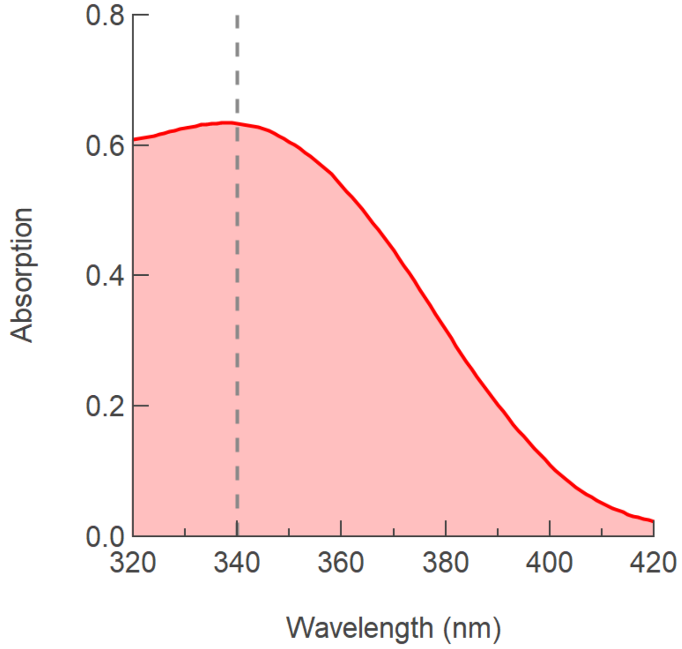

Figure S24: UV-vis absorption spectrum used for determining the nitrate content of UiO-66(Ce) after digestion in 1 M NH_4HCO_3. Note: BDC gives rise to absorption peaks around 300 nm, but the absorption of BDC at the concentration present in the MOF digestion solution was determined to have a negligible contribution towards the absorption at 340 nm.

From the nitrate concentration, we can then calculate the i variable in the minimal formula as:

$$i = \frac{[\mathrm{NO_3}]}{[UiO - 66(Ce)]} = \frac{1.7}{0.8} = 2.1 \tag{12}$$

8.1.4 Determination of hydroxide amount and water content

As done in the main text, the amount of hydroxides in the minimal formula can be adjusted to ensure that the Ce^{4+} cation in the node is charge balanced by the sum of all the anionic

species:

$$\begin{aligned}
C^{Ce^{4+}} &= C^{O^{2-}} + C^{OH^-} + C^{BDC^{2-}} + C^{NO_3^-} \\
6 \times 4 &= 4 \times 2 + 4 + w + 2x + i \\
24 &= 12 + w + (2 \times 4.5) + 2.1 \\
w &= 0.9
\end{aligned} \tag{13}$$

Thus, we get an anhydrous minimal formula:

$Ce_6O_4(\mu_3{-}OH)_4(OH)_{0.9}(BDC)_{4.5}(NO_3)_{2.1}(DMF)_{1.9}(EtOH)_{1.1}$ with a molar mass of 2046.3 g/mol.

Using equation 8, the amount of water in the MOF was found to be 13.2 per formula unit. Thus, the final experimental minimal formula for our UiO-66(Ce) is: $Ce_6O_4(\mu_3{-}OH)_4(OH)_{0.9}(BDC)_{4.5}(NO_3)_{2.1}(DMF)_{1.9}(EtOH)_{1.1}(H_2O)_{13.2}$

8.2 MOF-5(Zn): minimal formula

The minimal formula of an ideal MOF-5(Zn) is $Zn_4O(BDC)_3$, which has a molar mass of 769.9 g/mol. Therefore, to determine an experimental minimal formula, we start with the general formula $Zn_4O(BDC)_x(H_2O)_n$, where BDC is the benzenedicarboxylate linker.

8.2.1 TGA analysis to determine the molar mass of MOF

The complete combustion of an idealized MOF-5(Zn) is:

$$Zn_4O(BDC)_3 + 22.5O_2 \xrightarrow{\Delta} 4ZnO + 24CO_2 + 6H_2O \tag{14}$$

The formation of ZnO as the residual solid after heating up to 900 °C was confirmed by pXRD (Figure S25).

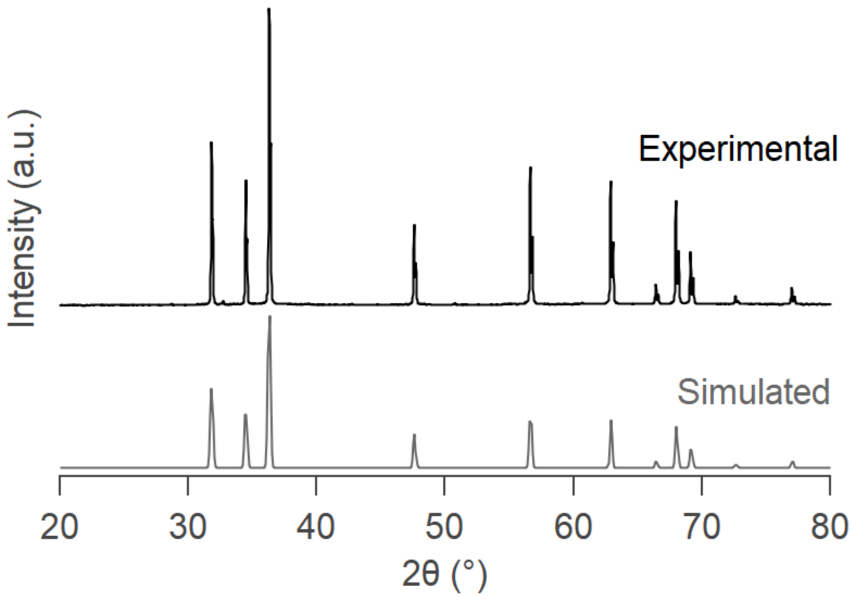

Figure S25: pXRD data confirming the formation of ZnO at the end of TGA. The simulated data for ZnO based on ICSD 26170 is given in grey.[S24]

The experimental molar mass of MOF-5(Zn) at room temperature (M_{exp}) can be calculated from the percentage residual inorganic weight of ZnO formed after complete combustion (W_{ZnO}), which corresponds to 4 molar equivalents of ZnO ($M_{\mathrm{ZnO}} = 81.4$ g/mol). This can be done using equation 15:

$$M_{exp} = \frac{100\%}{W_{\mathrm{ZnO}}} \times 4M_{\mathrm{ZnO}} = \frac{100\%}{40.9\%} \times 325.6 \tag{15}$$

Thus, the experimentally determined molar mass of MOF-5(Zn) is 796.0 g/mol based on a residual weight of 40.9% (Figure S26), which is slightly higher than the molar mass from the ideal minimal formula (769.9 g/mol).

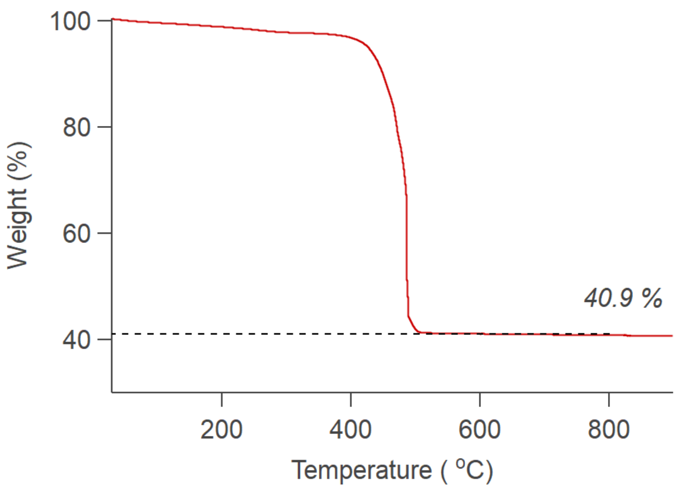

Figure S26: TGA analysis of MOF-5(Zn).

8.2.2 NMR analysis to determine the concentration of organics

A solution containing 1.82 mM MOF-5(Zn) (based on the experimental molar mass) and 2 mM TMSP-d_4 was prepared from the MOF digestion solution for quantitative NMR analysis (Figure S27).

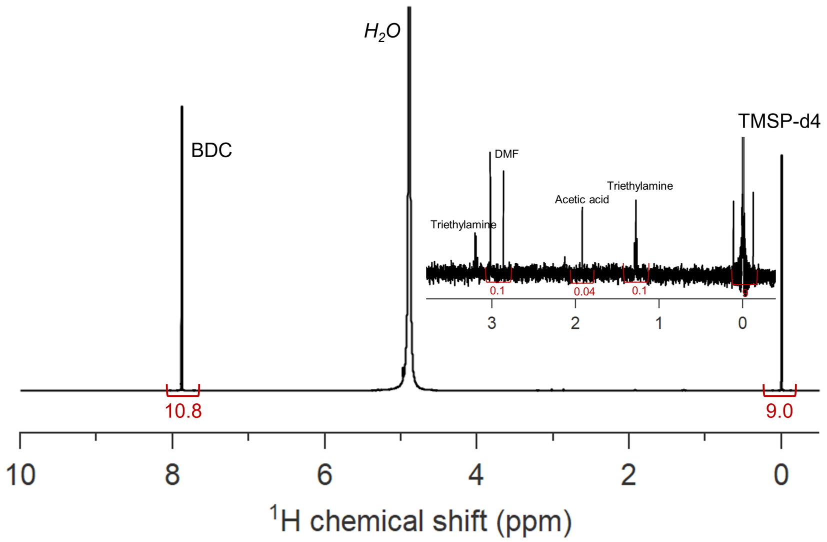

Figure S27: ^1H NMR spectrum in D_2O of 1.82 mM MOF-5(Zn) digested using 1 M NH_4HCO_3, with 2 mM TMSP-d_4 as internal standard. The inset shows the presence of weak peaks corresponding to acetic acid, triethylamine and DMF. The integration of all these peaks gives a number of equivalents lower than 0.1 and are deemed insignificant.

Using equation 3, we find a BDC concentration of 5.4 mM. Using the MOF concentration of 1.82 mM in the NMR tube, the amount of BDC (x) in the minimal formula, ($Zn_4O(BDC)_x(H_2O)_n$), can be easily calculated:

$$x = \frac{[BDC]}{[MOF-5(Zn)]} = \frac{5.4}{1.82} = 3.0 \tag{16}$$

Since the formula already is charge neutral, we don't need to charge balance. Also, the formula obtained is quite close to the ideal one, suggesting very few defects in this MOF. Thus, we get an anhydrous minimal formula: $Zn_4O(BDC)_3$ with a molar mass of 769.9 g/mol.

Using equation 8, the amount of water in the MOF was found to be 1.5 per formula unit. Thus, the final experimental minimal formula for our MOF-5(Zn) is:

$Zn_4O(BDC)_3(H_2O)_{1.5}$

8.3 MIL-125(Ti): minimal formula

The minimal formula of an ideal MIL-125(Ti) is $Ti_8O_8(OH)_4(BDC)_6$, which has a molar mass of 1563.7 g/mol. Therefore, to determine an experimental minimal formula, we start with the general formula $Ti_8O_8(OH)_{4+w}(BDC)_x(EtOH)_y(DMA)_z(TMA)_u(H_2O)_n$, where BDC is the benzenedicarboxylate linker, EtOH is used for washing the MOF, and DMA (dimethylamine) and TMA (trimethylamine) are the decomposition products of DMF, the solvent used for the synthesis.[S25,S26]

8.3.1 TGA analysis to determine the molar mass of MOF

The complete combustion of an idealized MIL-125(Ti) is:

$$Ti_8O_8(OH)_4(BDC)_6 + 52O_2 \xrightarrow{\Delta} 8TiO_2 + 48CO_2 + 28H_2O \tag{17}$$

The formation of TiO_2 as the residual solid after heating up to 900 °C was confirmed by

pXRD (Figure S28).

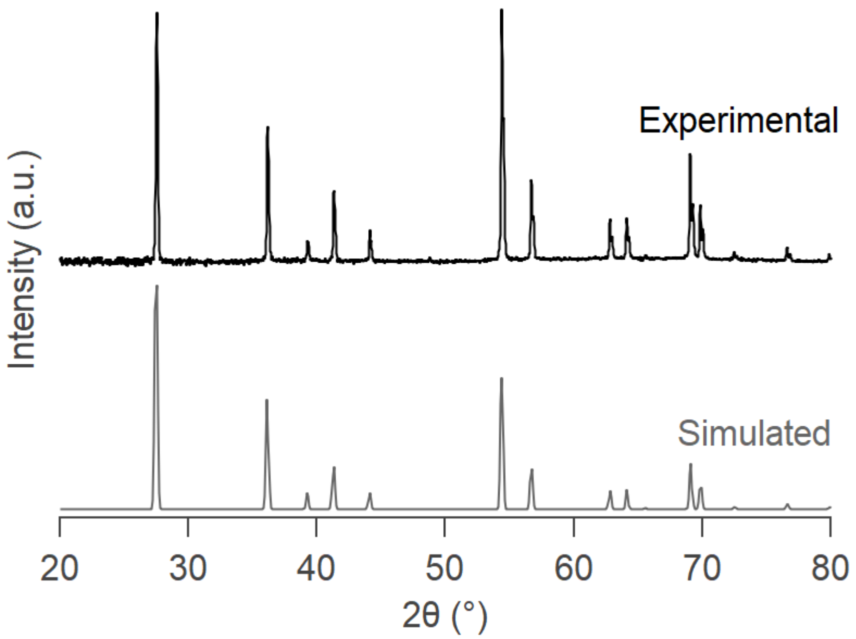

Figure S28: pXRD data confirming the formation of TiO_2 at the end of TGA. The simulated data for rutile TiO_2 based on ICSD 44881 is given in grey.[S27]

The experimental molar mass of MIL-125(Ti) at room temperature (M_{exp}) can be calculated from the percentage residual inorganic weight of TiO_2 formed after complete combustion ($W_{\mathrm{TiO_2}}$), which corresponds to 8 molar equivalents of TiO_2 ($M_{\mathrm{TiO_2}} = 79.86$ g/mol). This can be done using equation 18:

$$M_{exp} = \frac{100\%}{W_{\mathrm{TiO_2}}} \times 8M_{\mathrm{TiO_2}} = \frac{100\%}{30.2\%} \times 638.9 \tag{18}$$

Thus, the experimentally determined experimental molar mass of MIL-125(Ti) is 2115.6 g/mol based on a residual weight of 30.2% (Figure S29), which is higher than the molar mass from the ideal minimal formula (1563.6 g/mol). This significant difference is in part due to the adsorbed water in the MOF.

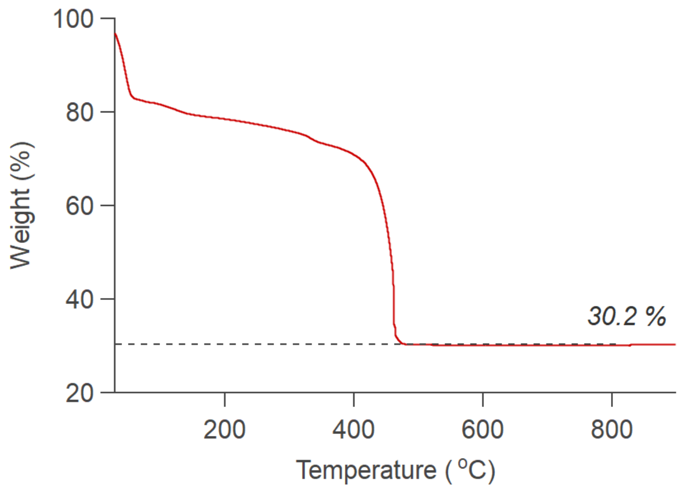

Figure S29: TGA analysis of MIL-125(Ti).

8.3.2 NMR analysis to determine the concentration of organics

A solution containing 1.5 mM MIL-125(Ti) (based on the experimental molar mass) and 2 mM TMSP-d_4 was prepared from the MOF digestion solution for quantitative NMR analysis (Figure S30).

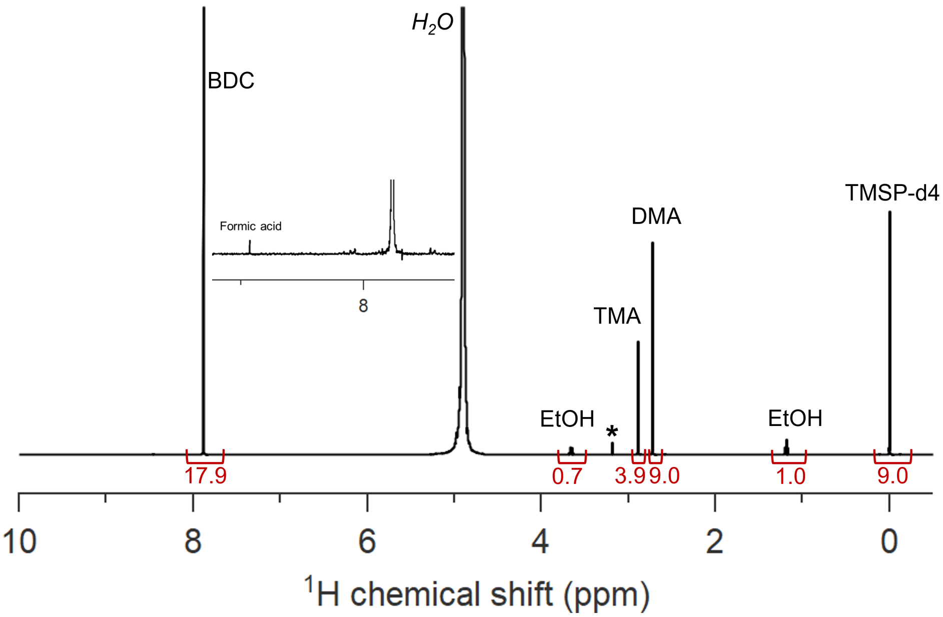

Figure S30: ^{1}H NMR spectrum in D_2O of 1.5 mM MIL-125(Ti) digested using 1 M NH_4HCO_3, with 2 mM TMSP-d_4 as internal standard. The inset shows the presence of a weak peak corresponding to formic acid and an unidentified peak at 3.2 ppm is labelled with an asterisk (*). The integration of the formic acid peak gives a number of equivalents lower than 0.1.

Using equation 3, we find BDC, EtOH, DMA, and TMA concentrations of 8.95 mM, 0.7 mM, 3 mM, and 0.9 mM, respectively. Using the MOF concentration of 1.5 mM in the NMR tube, the amount of BDC (x), EtOH (y), DMA (z), and TMA (u) in the minimal formula ($Ti_8O_8(OH)_{4+w}(BDC)_x(EtOH)_y(DMA)_z(TMA)_u(H_2O)_n$) can be easily calculated:

$$\begin{aligned} x &= \frac{[BDC]}{[MIL-125(Ti)]} = \frac{8.95}{1.5} = 6.0 \\ y &= \frac{[EtOH]}{[MIL-125(Ti)]} = \frac{0.7}{1.5} = 0.5 \\ z &= \frac{[DMA]}{[MIL-125(Ti)]} = \frac{3}{1.5} = 2.0 \\ u &= \frac{[TMA]}{[MIL-125(Ti)]} = \frac{0.9}{1.5} = 0.6 \end{aligned} \tag{19}$$

Since the formula already is charge neutral, a charge balancing step is not necessary. Also, the formula obtained is very close to the ideal one, suggesting very few defects in this MOF. However, it seems quite a lot of solvent is trapped in the pores of the MOF. Thus we get an anhydrous minimal formula: $Ti_8O_8(OH)_4(BDC)_6(EtOH)_{0.5}(DMA)_2(TMA)_{0.6}$ with a molar mass of 1712.3 g/mol.

Using equation 8, the amount of water in the MOF was found to be 22.4 per formula unit. Thus the final experimental minimal formula for our MIL-125(Ti) is:

$Ti_8O_8(OH)_4(BDC)_6(EtOH)_{0.5}(DMA)_2(TMA)_{0.6}(H_2O)_{22.4}$

8.4 MIL-100(Fe): minimal formula

The minimal formula of an ideal MIL-100(Fe) is $Fe_3O(OH)(H_2O)_2(BTC)_2$, which has a molar mass of 650.8 g/mol. Therefore, to determine an experimental minimal formula, we start with the general formula $Fe_3O(OH)_w(BTC)_x(MeOH)_y(Cl)_i(H_2O)_n$, where BTC is the benzenetricarboxylate linker, MeOH is used for washing the MOF, and chloride comes from the precursor, $FeCl_3 \cdot 6H_2O$.

8.4.1 TGA analysis to determine the molar mass of MOF

The complete combustion of an idealized MIL-100(Fe) is:

$$2\,[Fe_3O(OH)(H_2O)_2(BTC)_2] + 30O_2 \xrightarrow{\Delta} 3\,Fe_2O_3 + 36CO_2 + 11H_2O \tag{20}$$

The formation of Fe_2O_3 as the residual solid after heating up to 900 °C was confirmed by pXRD (Figure S31).

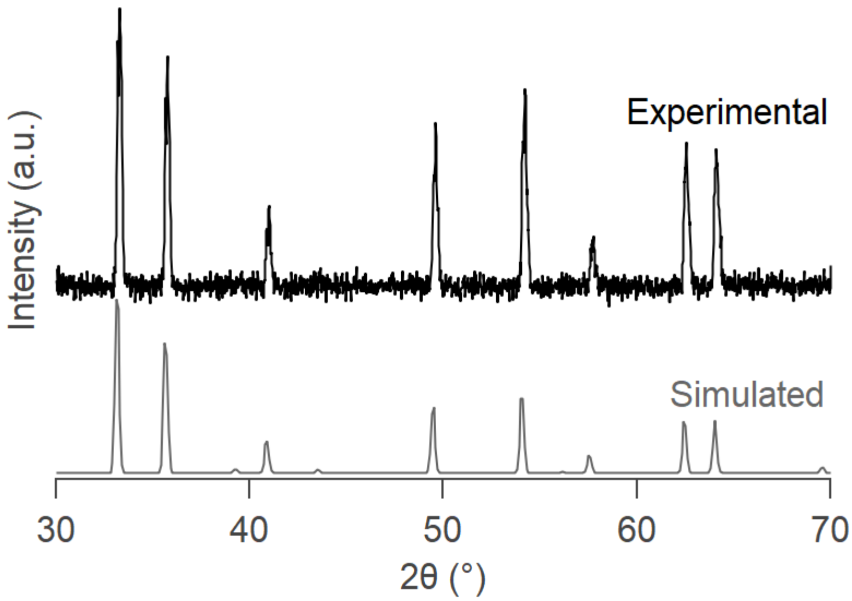

Figure S31: pXRD data confirming the formation of Fe_2O_3 at the end of TGA. The simulated data for hematite, α-Fe_2O_3, based on ICSD 15840 is given in grey.[S28]

The experimental molar mass of MIL-100(Fe) at room temperature (M_{exp}) can be calculated from the percentage residual inorganic weight of Fe_2O_3 formed after complete combustion ($W_{Fe_2O_3}$), which corresponds to 1.5 molar equivalents of Fe_2O_3 ($M_{Fe_2O_3} = 159.7$ g/mol). This can be done using equation 21:

$$M_{exp} = \frac{100\%}{W_{Fe_2O_3}} \times 1.5 M_{Fe_2O_3} = \frac{100\%}{29.1\%} \times 239.6 \tag{21}$$

Thus, the experimentally determined molar mass of MIL-100(Fe) is 823.4 g/mol based

on a residual weight of 29.1% (Figure S32), which is significantly higher than the molar mass from the ideal minimal formula (650.8 g/mol).

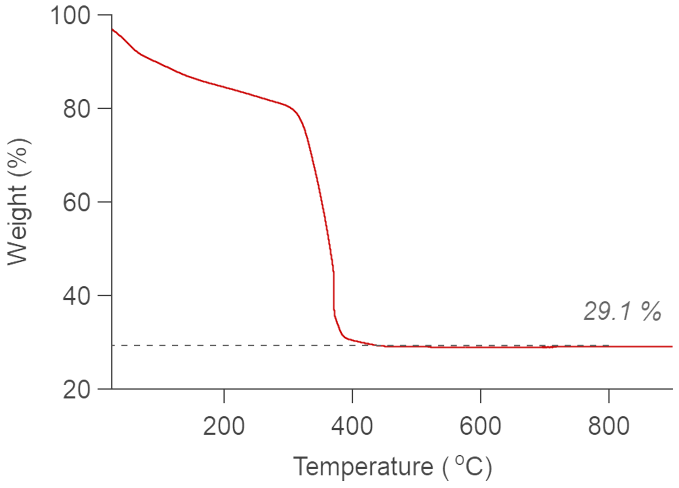

Figure S32: TGA analysis of MIL-100(Fe).

8.4.2 NMR analysis to determine the concentration of organics

A solution containing 1.04 mM MIL-100(Fe) (based on the experimental molar mass) and 2 mM TMSP-d_4 was prepared from the MOF digestion solution for quantitative NMR analysis (Figure S33).

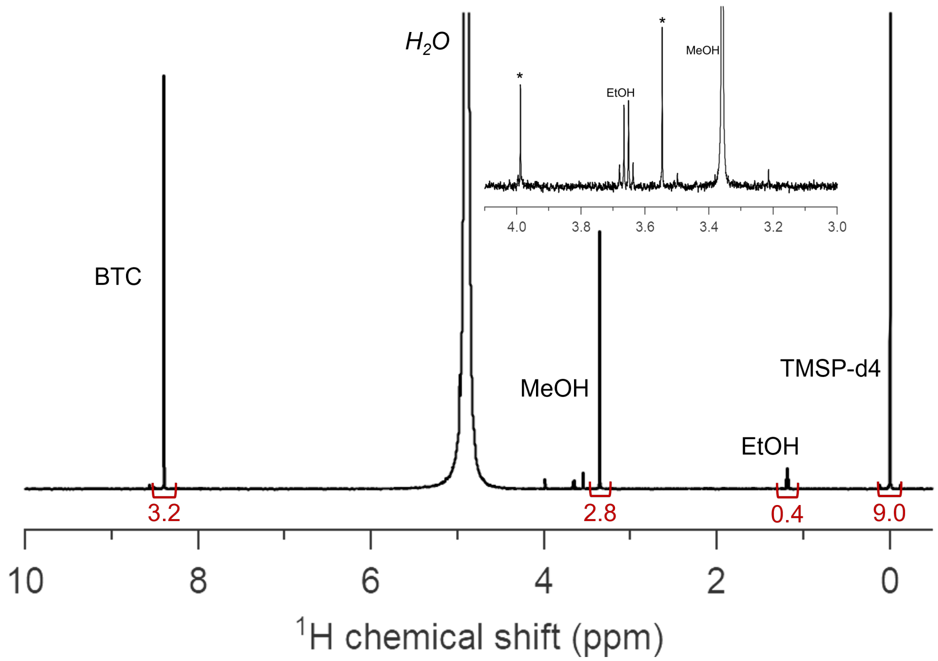

Figure S33: ^{1}H NMR spectrum in D_2O of 1.04 mM MIL-100(Fe) digested using 1 M NH_4HCO_3, with 2 mM TMSP-d_4 as internal standard. The inset clearly shows the splitting of one of the EtOH peaks, suggesting the absence of peak broadening due to paramagnetism and some unidentified peaks labelled with asterisks (*).

Using equation 3, we find BTC, MeOH, and EtOH concentrations of 2.1 mM, 1.9 mM, and 0.3 mM, respectively. Using the MOF concentration of 1.04 mM in the NMR tube, the amount of BDC (x), MeOH (y), and EtOH (z) in the minimal formula, $(Fe_3O(OH)_w(BTC)_x(MeOH)_y(EtOH)_z(Cl)_i(H_2O)_n)$, can be easily calculated:

$$\begin{aligned} x &= \frac{[BTC]}{[MIL-100(Fe)]} = \frac{2.1}{1.04} = 2.0 \\ y &= \frac{[MeOH]}{[MIL-100(Fe)]} = \frac{1.9}{1.04} = 1.8 \\ z &= \frac{[EtOH]}{[MIL-100(Fe)]} = \frac{0.3}{1.04} = 0.3 \end{aligned} \tag{22}$$

8.4.3 UV-vis analysis to determine the amount of inorganic ligands

Now to determine the amount of chloride in the sample, we turned to UV-vis spectroscopy. A MIL-100(Fe) solution of 1.04 mM concentration shows the presence of 0.8 mM chloride

based on the absorption at 500 nm.

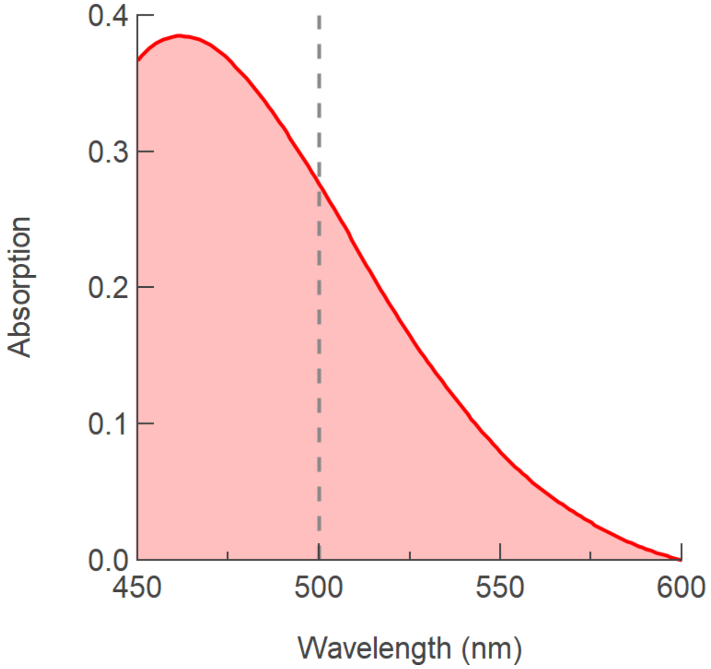

Figure S34: UV-vis absorption spectrum used for determining the chloride content of MIL-100(Fe) after digestion in 1 M NH_4HCO_3.

From this concentration, we can then calculate the i variable in the minimal formula as

$$i = \frac{[Cl]}{[MIL-100(Fe)]} = \frac{0.8}{1.04} = 0.8 \tag{23}$$

From the above analysis, we arrive at the minimal formula:

$Fe_3O(OH)_w(BTC)_2(MeOH)_{1.8}(EtOH)_{0.3}(Cl)_{0.8}(H_2O)_n$

8.4.4 Determination of hydroxide amount for charge balancing

As done in the main text, the amount of hydroxides in the minimal formula can be adjusted to ensure that the 3 Fe^{3+} cations in the cluster are charge balanced by the sum of all the

anionic species:

$$\begin{aligned} C^{Fe^{3+}} &= C^{O^{2-}} + C^{OH^-} + C^{BTC^{3-}} + C^{Cl^-} \\ 3 \times 3 &= 1 \times 2 + w + 3x + i \\ 9 &= 2 + w + (3 \times 2) + 0.8 \\ w &= 0.2 \end{aligned} \tag{24}$$

Thus, we get an anhydrous minimal formula: $Fe_3O(OH)_{0.2}(BTC)_2(MeOH)_{1.8}(EtOH)_{0.3}(Cl)_{0.8}$ with a molar mass of 701 g/mol.

Using equation 8, the amount of water in the MOF was found to be 6.8 per formula unit. Thus, the final experimental minimal formula for our MIL-100(Fe) is:

$Fe_3O(OH)_{0.2}(BTC)_2(MeOH)_{1.8}(EtOH)_{0.3}(Cl)_{0.8}(H_2O)_{6.8}$

9 Minimal formula for unknown frameworks

This section showcases the implementation of our methodology to samples for which there is a lack of structural information. The MOF that we analyse was made using $AlCl_3$ (2.1 mmol, 0.281 g) and H_2BDC (3.37 mmol, 0.560 g) in 30 mL of DMF. The FTIR spectrum of the obtained MOF seems to match fairly well with the expected spectrum for MIL-53(Al) (Figure S35). However, the pXRD pattern (Figure S36) does not appear to match with the reported pXRD patterns of known structures of Al-BDC MOFs, such as MIL-53(Al). Nevertheless, we have confirmed its porosity through adsorption measurements (Figure S37), which demonstrate that it is an Al-BDC MOF whose exact structure is unknown. For samples like this, we can still assign the number of equivalents of organic moieties with respect to the metal atom that is present. On the other hand, for frameworks with metal-oxo clusters as secondary building units, charge balancing is more challenging as the number of oxo (O^{2-}) and hydroxide ligands that form the metal-containing node of the MOF is not known.

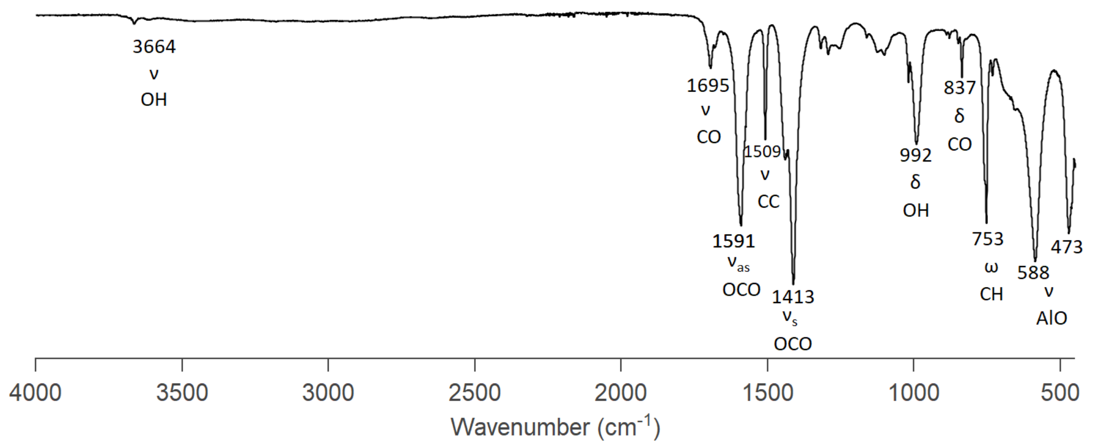

Figure S35: FTIR spectrum of Al-BDC MOF, which matches reasonably well with the FTIR spectra reported in the literature for MIL-53(Al) and can be assigned based on computationally calculated active vibrational frequencies.[S29]

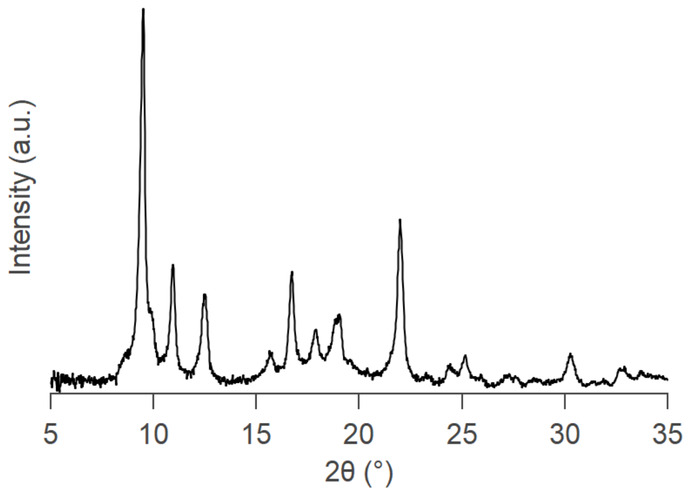

Figure S36: pXRD data for Al-BDC MOF.

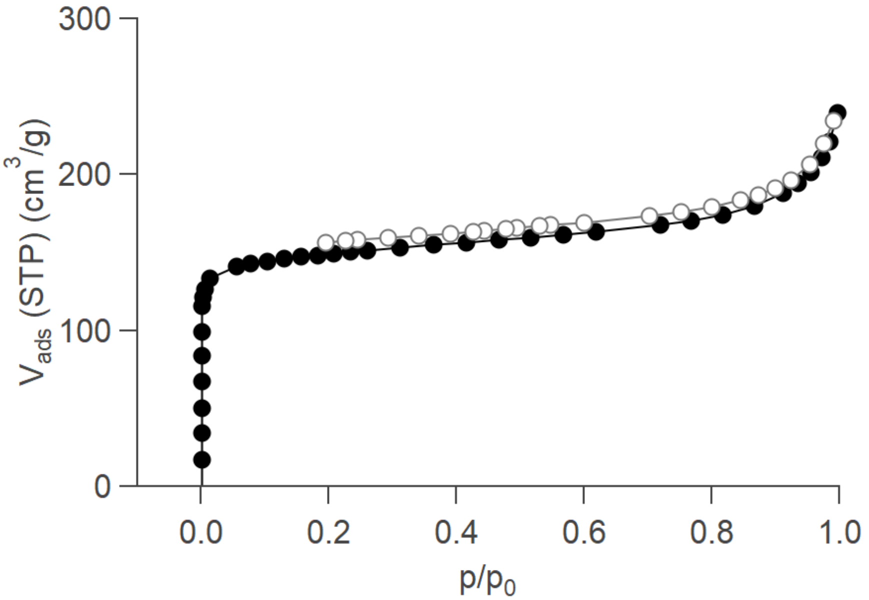

Figure S37: Nitrogen sorption measurement of Al-BDC MOF. The filled symbols represent adsorption and empty symbols represent the desorption step

9.1 Al-BDC MOF: minimal formula

To determine an experimental minimal formula, we start with the general formula $Al(OH)_w(BDC)_x(H_2O)_n$, where BDC is the benzenedicarboxylate linker (assuming there are no oxo ligands like in MIL-53). Chloride content is not considered for the experimental minimal formula of this Al-MOF, even though it was synthesized from $AlCl_3$, since the amount of Cl was evaluated using the commercial photometric method described above and no Cl was detected, indicating Cl does not become incorporated into the MOF.

9.1.1 TGA analysis to determine the molar mass of MOF

The complete combustion of this Al-MOF is:

$$2\,[Al(OH)(BDC)] + 15O_2 \xrightarrow{\Delta} Al_2O_3 + 16CO_2 + 5H_2O \tag{25}$$

The formation of Al_2O_3 as the residual solid after heating up to 900 °C was confirmed by pXRD (Figure S38).

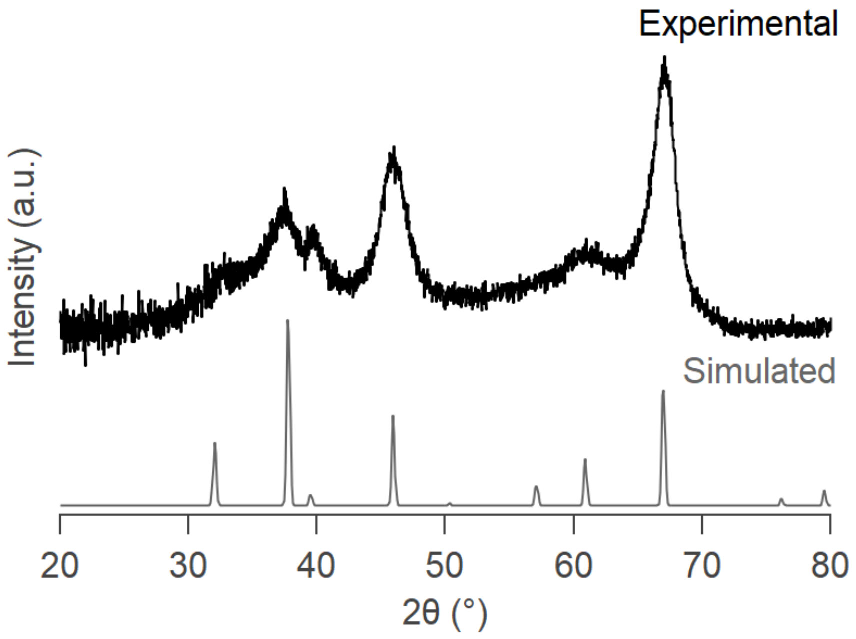

Figure S38: pXRD data confirming the formation of γ-Al_2O_3 at the end of TGA. The simulated data for γ-Al_2O_3 based on ICSD 38876 is given in grey.[S30]

The experimental molar mass of Al-MOF at room temperature (M_{exp}) can be calculated from the percentage residual inorganic weight of Al_2O_3 formed after complete combustion ($W_{Al_2O_3}$), which corresponds to 0.5 molar equivalents of Al_2O_3 ($M_{Al_2O_3}$ = 101.96 g/mol). This can be done using equation 26:

$$M_{exp} = \frac{100\%}{W_{Al_2O_3}} \times 0.5 M_{Al_2O_3} = \frac{100\%}{22.5\%} \times 51 \tag{26}$$

Thus, the experimentally determined molar mass of Al-MOF is 226.7 g/mol based on a residual weight of 22.5% (Figure S39).

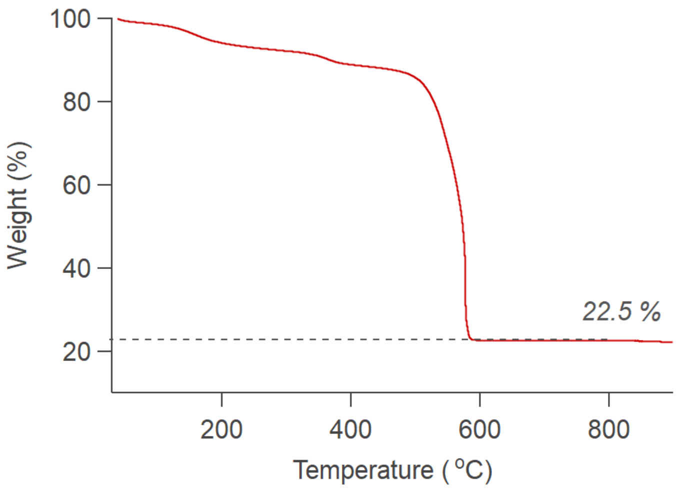

Figure S39: TGA analysis of Al-MOF.

9.1.2 NMR analysis to determine the concentration of organics

A solution containing 1.5 mM Al-MOF (based on the experimental molar mass) and 2 mM TMSP-d_4 was prepared from the MOF digestion solution for quantitative NMR analysis (Figure S40).

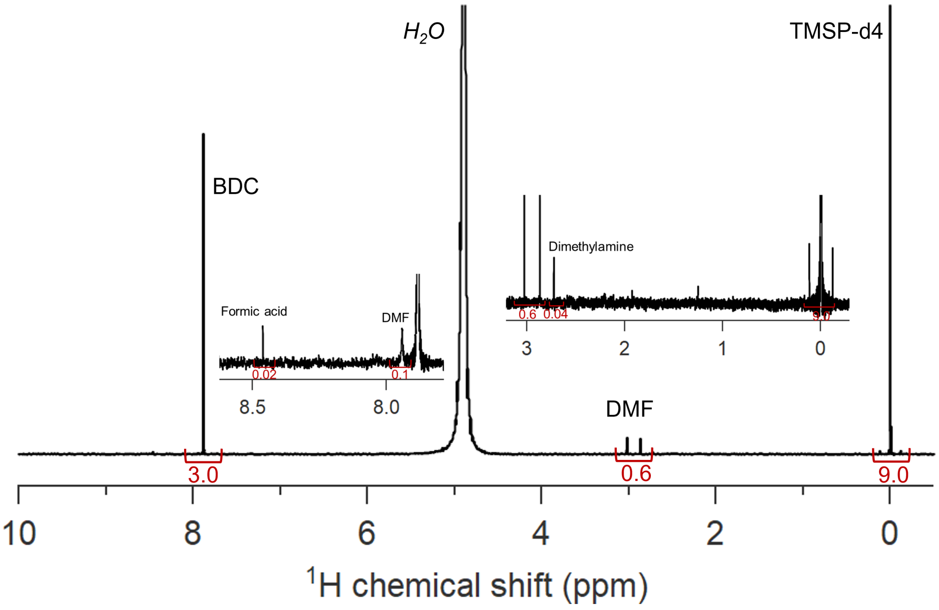

Figure S40: ^{1}H NMR spectrum in D_2O of 1.5 mM Al-MOF digested using 1 M NH_4HCO_3, with 2 mM TMSP-d_4 as internal standard. The inset shows the presence of weak peaks corresponding to dimethyl amine, formic acid and the aldehyde proton of DMF as confirmed by its matching integration with the methyl protons of DMF. The integration of the dimethyl amine and formic acid peaks gives a number of equivalents lower than 0.1 for each, which we consider to be negligible.

Using equation 3, we find a BDC concentration of 1.5 mM and a DMF concentration of 0.2 mM. Using the MOF concentration of 1.5 mM in the NMR tube, the amount of BDC (x) and DMF (y) in the minimal formula, $(Al(OH)_w(BDC)_x(DMF)_y(H_2O)_n)$, can be easily calculated:

$$\begin{aligned} x &= \frac{[BDC]}{[MIL-53(Al)]} = \frac{1.5}{1.5} = 1.0 \\ y &= \frac{[DMF]}{[MIL-53(Al)]} = \frac{0.2}{1.5} = 0.1 \end{aligned} \tag{27}$$

As done in the main text, the amount of hydroxides in the minimal formula can be adjusted to ensure that the Al^{3+} cation in the node is charge balanced by the sum of all the

anionic species:

$$C^{Al^{3+}} = C^{OH^-} + C^{BDC^{2-}}$$
$$3 = w + (2 \times 1) \tag{28}$$
$$w = 1$$

Thus, we get an anhydrous minimal formula: $Al(OH)(BDC)(DMF)_{0.1}$ with a molar mass of 215.4 g/mol.

Using equation 8, the amount of water in the MOF was found to be 0.6 per formula unit. Thus, the final experimental experimental minimal formula for our Al-MOF is:

$Al(OH)(BDC)(DMF)_{0.1}(H_2O)_{0.6}$

10 Supporting Information for Main Text

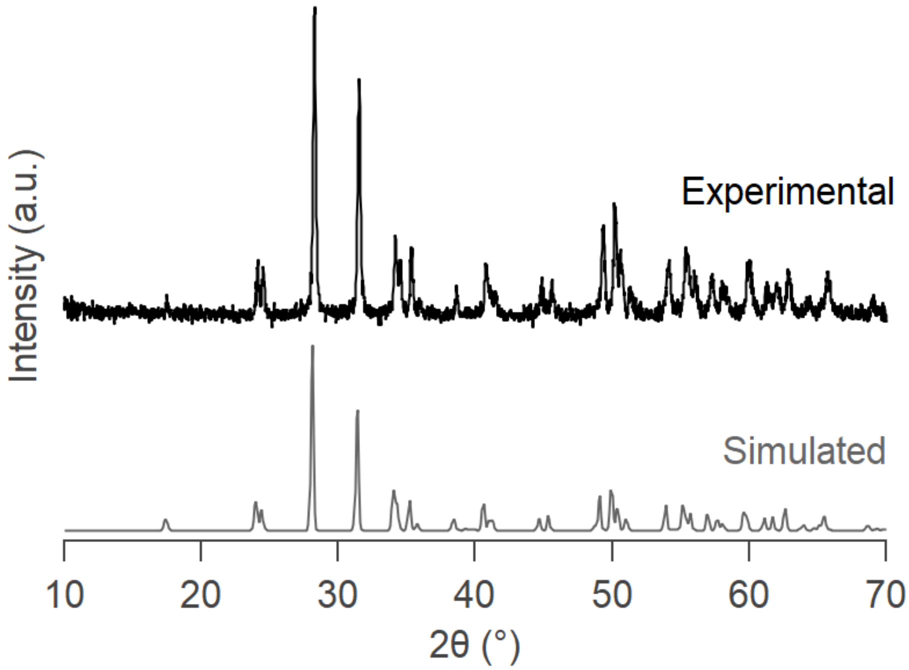

Figure S41: pXRD data confirming the formation of monoclinic ZrO_2 at the end of TGA of MOF-808(Zr). The simulated data for ZrO_2 based on ICSD 26488 is given in grey.[S21]

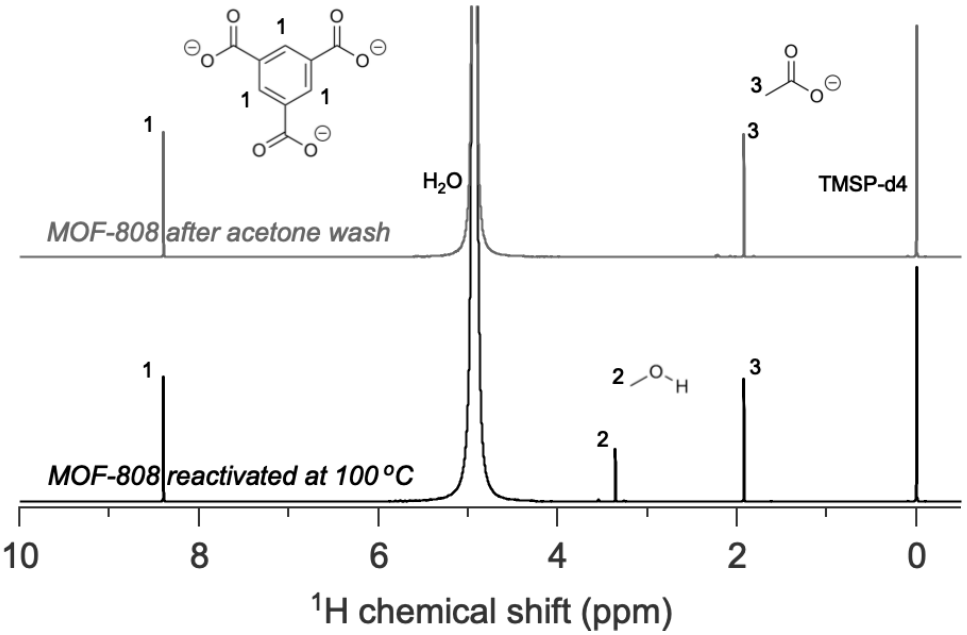

Figure S42: ^{1}H NMR spectra for MOF-808(Zr) digested in 1M NH_4HCO_3 in D_2O (bottom spectrum) after reactivation at 100 °C and (top spectrum) after solvent wash using acetone. The solvent wash was done by stirring the activated MOF-808(Zr) in acetone for 2 days and activating it again using the procedure described in the experimental section.

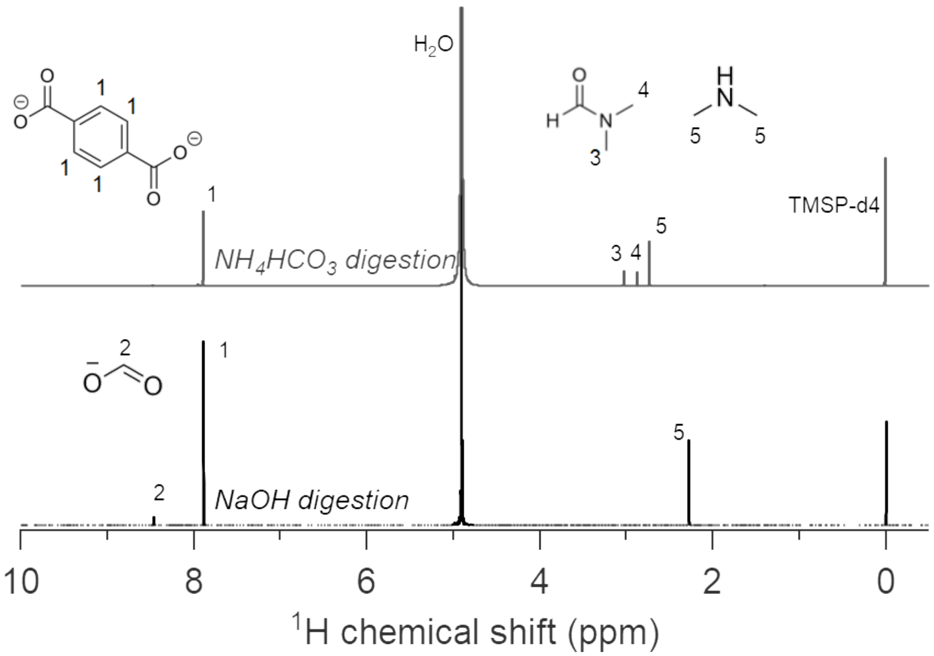

Figure S43: ^{1}H NMR spectra for UiO-66(Zr) digested in (bottom spectrum) 1M NaOH in D_2O and (top spectrum) 1M NH_4HCO_3 in D_2O.

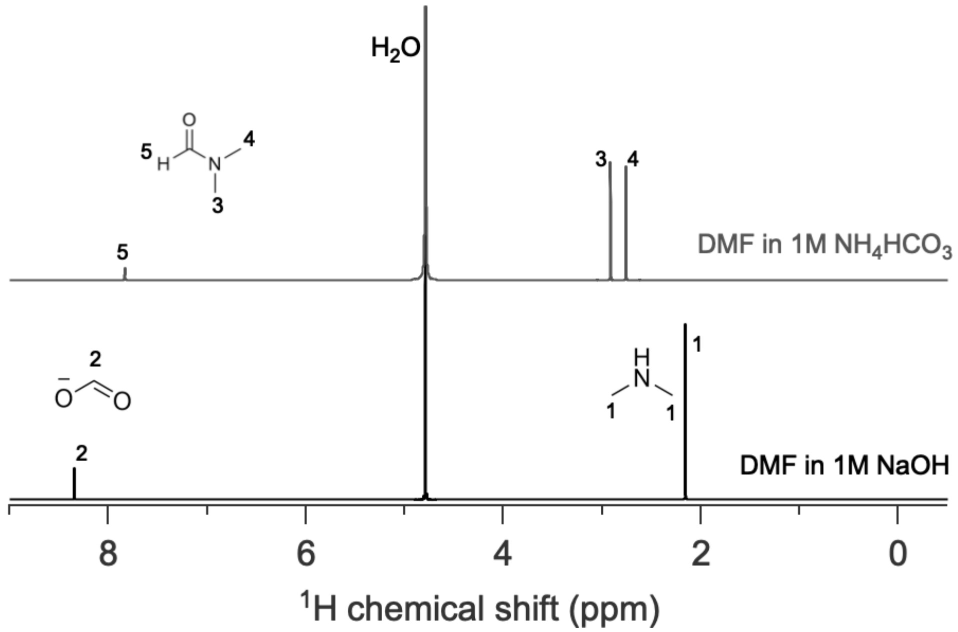

Figure S44: ^{1}H NMR spectra for DMF dissolved in (bottom spectrum) 1M NaOH in D_2O and (top spectrum) 1M NH_4HCO_3 in D_2O.

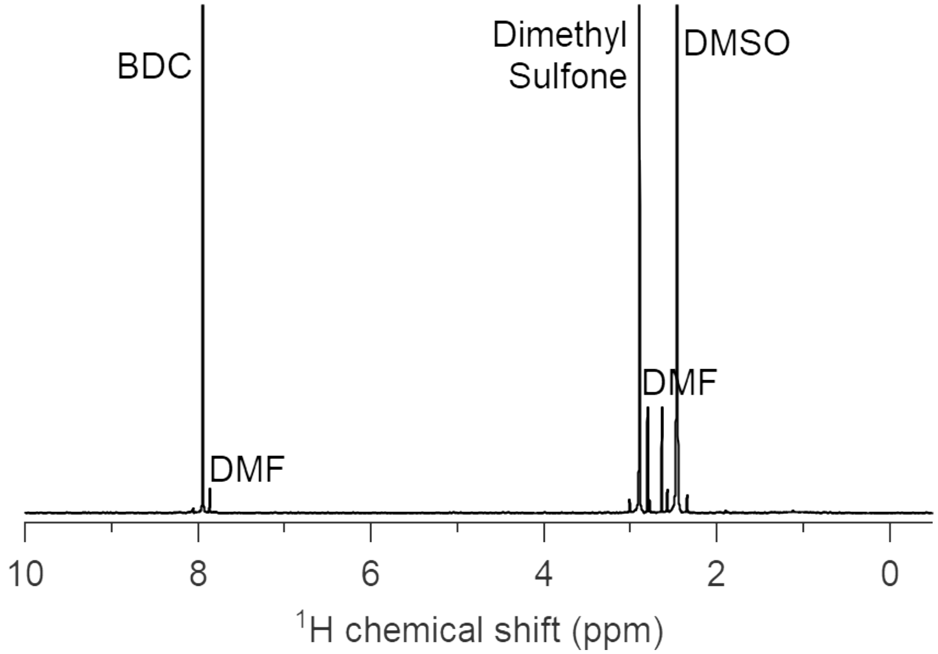

Figure S45: ^{1}H NMR spectra for UiO-66(Zr) digested in D_2SO_4/DMSO solution. Dimethyl-sulfone was used as the internal standard.

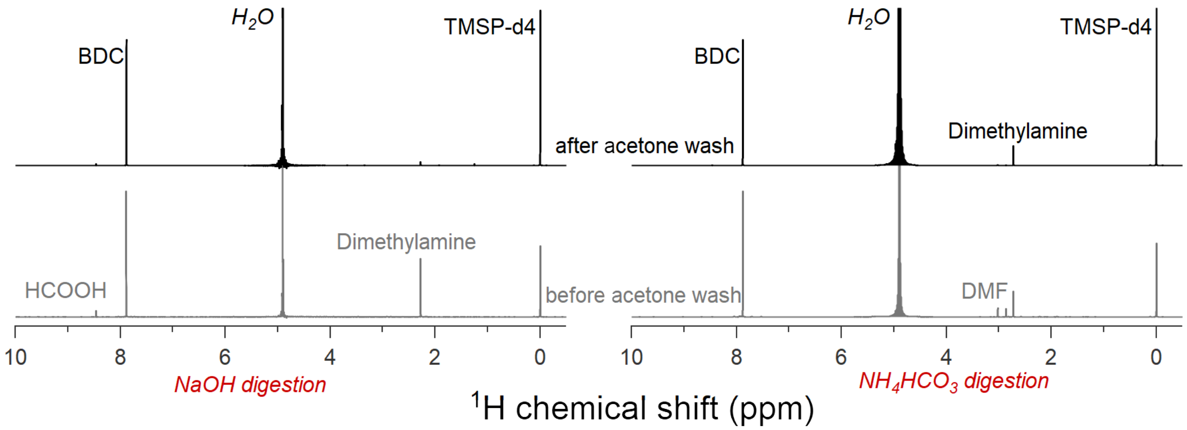

Figure S46: ^{1}H NMR spectra for UiO-66(Zr) before (grey) and after acetone washing (black). The digestion was done in 1M NaOH in D_2O (left spectra) or 1M NH_4HCO_3 in D_2O (right spectra). The peaks are normalised with respect to the BDC peak.

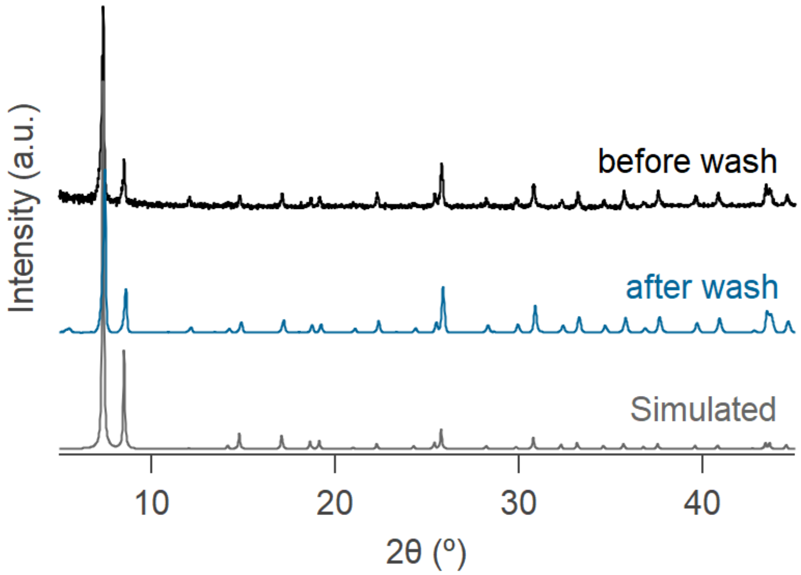

Figure S47: pXRD data for UiO-66(Zr) before (black) and after washing with acetone (blue) demonstrating that the MOF remains stable. The simulated data is given in grey and is based on the data from CCDC 837796.

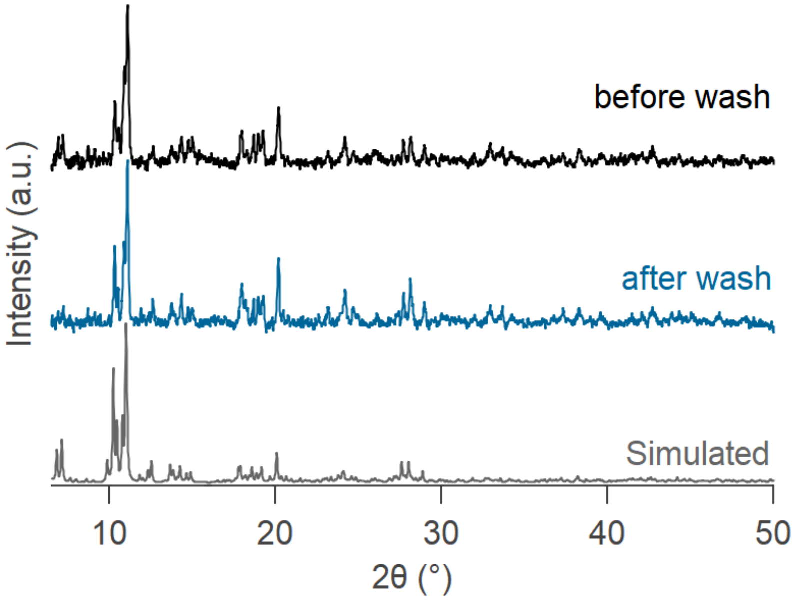

Figure S48: pXRD data for MIL-100(Fe) before (black) and washing with acetone (blue) demonstrating that the MOF remains stable. The simulated data is given in grey and is based on data from CCDC 640536.[S14]

Table S1: T_1 relaxation time and recommended relaxation delay (D1) of non-linker organic components of MOFs and the internal standard. The measurements were done on a 600 MHz spectrometer for 1M NH_4HCO_3 solutions in D_2O.

**Molecule**	**T_1**	**D1**
TMSP-d_4	3.4 s	17 s
Formic acid	20 s	100 s
Acetic acid	5.6 s	28 s
Methanol	8.1 s	40.5 s
Dimethylamine.HCl	5.1 s	25.5 s
DMF	9.4 s, 4.3 s, 7.3 s	47 s, 21.5 s, 36.5 s

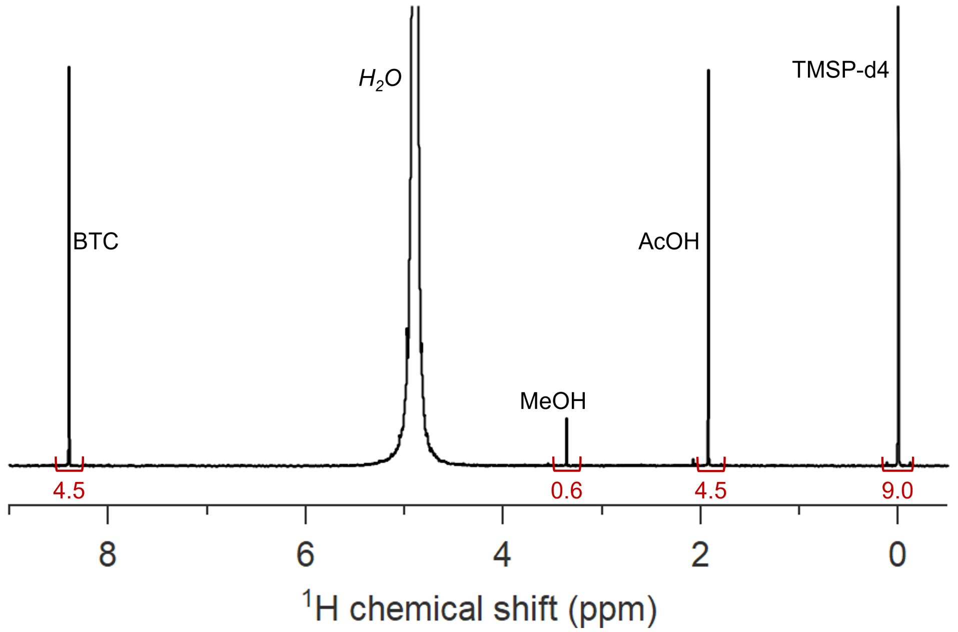

Figure S49: ^{1}H NMR spectrum in D_2O of 1.5 mM MOF-808(Zr) digested using 1M NH_4HCO_3, with 2 mM TMSP-d_4 as internal standard.

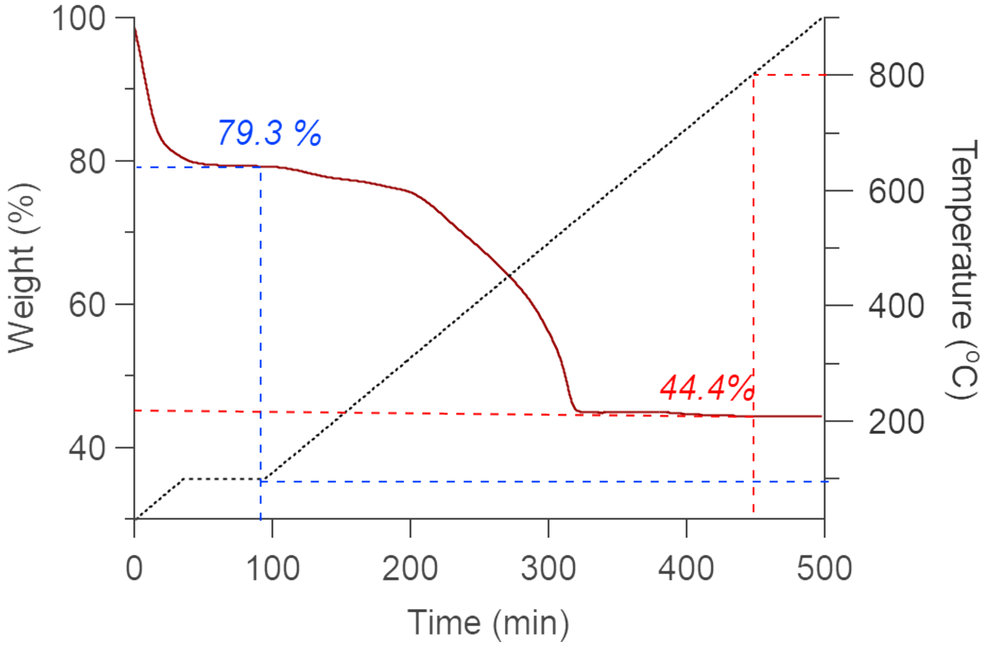

Figure S50: TGA analysis of MOF-808(Zr) with a 1 h isotherm at 100 °C. This is done to remove all the water molecules that are trapped in the pores of the MOF. The red lines show the TGA profile over time and the black line corresponds to the temperature change over time.

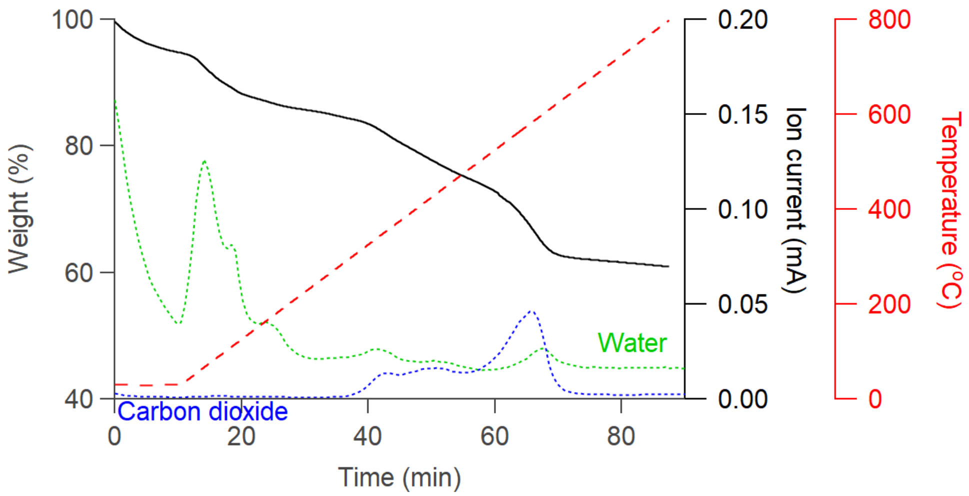

Figure S51: TGA-MS analysis of MOF-808(Zr). The black line corresponds to the sample combustion with temperature (red line) under N_2 atmosphere. The ionic current corresponding to 18 amu (water; green line) and 44 amu (carbon dioxide; blue line) is plotted with respect to time.

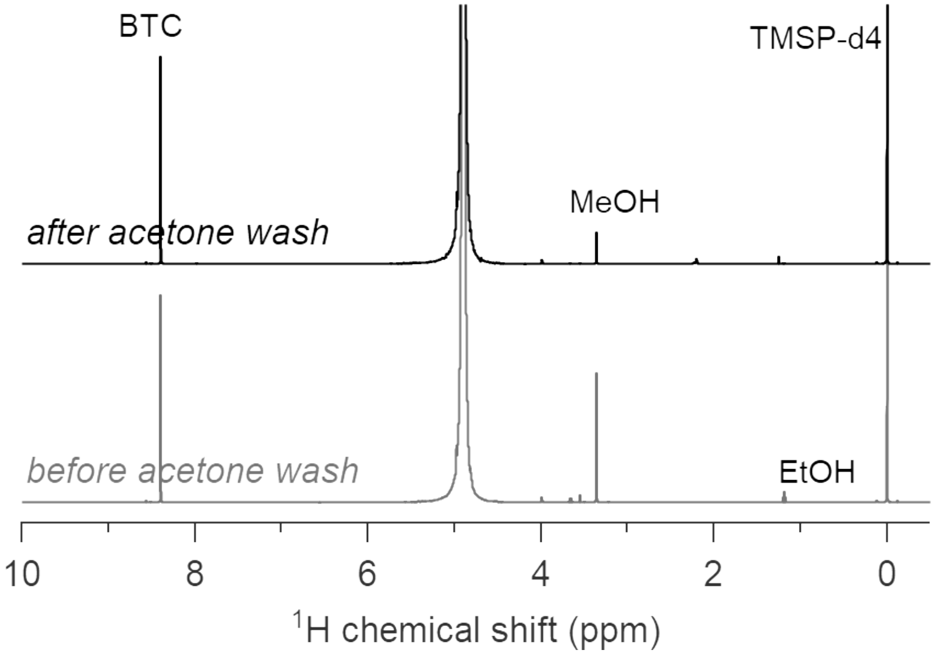

Figure S52: ^{1}H NMR spectra for MIL-100(Fe) digested in 1M NH_4HCO_3 in D_2O (bottom spectrum) control, (top spectrum) after solvent wash using acetone. The solvent wash is done by stirring the activated MIL-100(Fe) in acetone for 24 h and activating it again under vacuum.

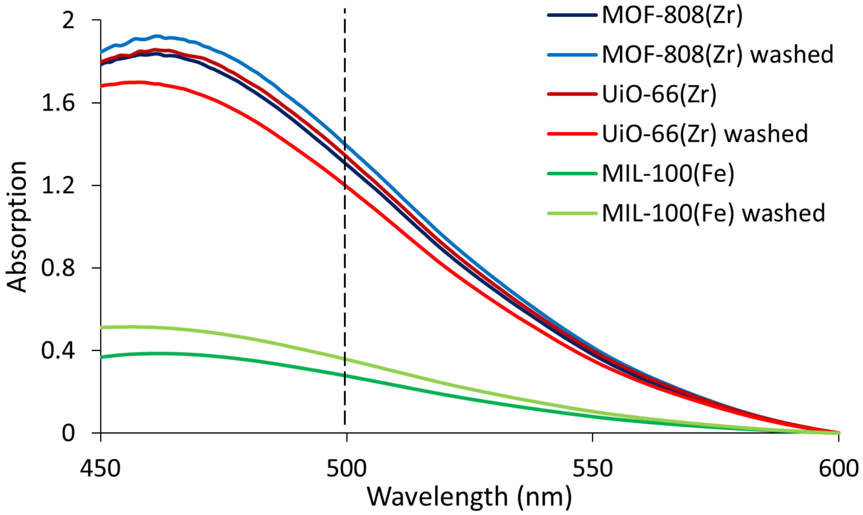

Figure S53: UV-vis absorption spectra used for determining the chloride content of digestion solutions in 1 M NH_4HCO_3 of UiO-66(Zr), MOF-808(Zr), and MIL-100(Fe) before and after washing for several hours with acetone followed by re-activation showing very little change in the chloride content after washing. The spectra for the washed MOFs were scaled based on the concentration of the MOF (to determine the MOF concentration, for UiO-66(Zr) and MOF808-66(Zr) the experimental molar mass of the MOF before washing was used but for MIL-100(Fe) the ideal molar mass was used because removal of solvent from MIL-100(Fe) has a larger effect on its minimal formula and should give a minimal formula close to the ideal).

References

(S1) Reinsch, H.; Waitschat, S.; Chavan, S. M.; Lillerud, K. P.; Stock, N. A facile “green” route for scalable batch production and continuous synthesis of zirconium MOFs. *European Journal of Inorganic Chemistry* **2016**, *2016*, 4490–4498.

(S2) Vermoortele, F.; Bueken, B.; Le Bars, G.; Van de Voorde, B.; Vandichel, M.; Houthoofd, K.; Vimont, A.; Daturi, M.; Waroquier, M.; Van Speybroeck, V., et al. Synthesis modulation as a tool to increase the catalytic activity of metal–organic frameworks: the unique case of UiO-66 (Zr). *Journal of the American Chemical Society* **2013**, *135*, 11465–11468.

(S3) Lammert, M.; Wharmby, M. T.; Smolders, S.; Bueken, B.; Lieb, A.; Lo-

machenko, K. A.; De Vos, D.; Stock, N. Cerium-based metal organic frameworks with UiO-66 architecture: synthesis, properties and redox catalytic activity. *Chemical Communications* **2015**, *51*, 12578–12581.

(S4) Tranchemontagne, D. J.; Hunt, J. R.; Yaghi, O. M. Room temperature synthesis of metal-organic frameworks: MOF-5, MOF-74, MOF-177, MOF-199, and IRMOF-0. *Tetrahedron* **2008**, *64*, 8553–8557.

(S5) Dan-Hardi, M.; Serre, C.; Frot, T.; Rozes, L.; Maurin, G.; Sanchez, C.; Férey, G. A new photoactive crystalline highly porous titanium (IV) dicarboxylate. *Journal of the American Chemical Society* **2009**, *131*, 10857–10859.

(S6) Schertenleib, T.; Karve, V. V.; Stoian, D.; Asgari, M.; Trukhina, O.; Oveisi, E.; Mensi, M.; Queen, W. L. A post-synthetic modification strategy for enhancing Pt adsorption efficiency in MOF/polymer composites. *Chemical Science* **2024**, *15*, 8323–8333.

(S7) Moumen, E.; Bazzi, L.; El Hankari, S. Aluminum-fumarate based MOF: A promising environmentally friendly adsorbent for the removal of phosphate. *Process Safety and Environmental Protection* **2022**, *160*, 502–512.

(S8) Furukawa, H.; Gándara, F.; Zhang, Y.-B.; Jiang, J.; Queen, W. L.; Hudson, M. R.; Yaghi, O. M. Water adsorption in porous metal-organic frameworks and related materials. *Journal of the American Chemical Society* **2014**, *136*, 4369–4381.

(S9) Valenzano, L.; Civalleri, B.; Chavan, S.; Bordiga, S.; Nilsen, M. H.; Jakobsen, S.; Lillerud, K. P.; Lamberti, C. Disclosing the complex structure of UiO-66 metal organic framework: a synergic combination of experiment and theory. *Chemistry of Materials* **2011**, *23*, 1700–1718.

(S10) Romero-Muniz, I.; Romero-Muniz, C.; del Castillo-Velilla, I.; Marini, C.; Calero, S.; Zamora, F.; Platero-Prats, A. E. Revisiting vibrational spectroscopy to tackle the

chemistry of Zr6O8 metal-organic framework nodes. *ACS applied materials & interfaces* **2022**, *14*, 27040–27047.

(S11) Cavka, J. H.; Jakobsen, S.; Olsbye, U.; Guillou, N.; Lamberti, C.; Bordiga, S.; Lillerud, K. P. A new zirconium inorganic building brick forming metal organic frameworks with exceptional stability. *Journal of the American Chemical Society* **2008**, *130*, 13850–13851.

(S12) Han, Y.; Liu, M.; Li, K.; Zuo, Y.; Wei, Y.; Xu, S.; Zhang, G.; Song, C.; Zhang, Z.; Guo, X. Facile synthesis of morphology and size-controlled zirconium metal–organic framework UiO-66: the role of hydrofluoric acid in crystallization. *CrystEngComm* **2015**, *17*, 6434–6440.

(S13) Li, H.; Eddaoudi, M.; O'Keeffe, M.; Yaghi, O. M. Design and synthesis of an exceptionally stable and highly porous metal-organic framework. *nature* **1999**, *402*, 276–279.

(S14) Horcajada, P.; Surblé, S.; Serre, C.; Hong, D.-Y.; Seo, Y.-K.; Chang, J.-S.; Grenèche, J.-M.; Margiolaki, I.; Férey, G. Synthesis and catalytic properties of MIL-100 (Fe), an iron (III) carboxylate with large pores. *Chemical Communications* **2007**, 2820–2822.

(S15) Pallach, R.; Keupp, J.; Terlinden, K.; Frentzel-Beyme, L.; Kloß, M.; Machalica, A.; Kotschy, J.; Vasa, S. K.; Chater, P. A.; Sternemann, C., et al. Frustrated flexibility in metal-organic frameworks. *Nature communications* **2021**, *12*, 4097.

(S16) Civalleri, B.; Napoli, F.; Noël, Y.; Roetti, C.; Dovesi, R. Ab-initio prediction of materials properties with CRYSTAL: MOF-5 as a case study. *CrystEngComm* **2006**, *8*, 364–371.

(S17) Sun, Y.; Ji, H.; Sun, Y.; Zhang, G.; Zhou, H.; Cao, S.; Liu, S.; Zhang, L.; Li, W.; Zhu, X., et al. Synergistic effect of oxygen vacancy and high porosity of nano MIL-125

(Ti) for enhanced photocatalytic nitrogen fixation. *Angewandte Chemie International Edition* **2024**, *63*, e202316973.

(S18) Hadjiivanov, K. I.; Panayotov, D. A.; Mihaylov, M. Y.; Ivanova, E. Z.; Chakarova, K. K.; Andonova, S. M.; Drenchev, N. L. Power of infrared and Raman spectroscopies to characterize metal-organic frameworks and investigate their interaction with guest molecules. *Chemical Reviews* **2020**, *121*, 1286–1424.

(S19) Rostamnia, S.; Alamgholiloo, H. Synthesis and catalytic application of mixed valence iron (Fe II/Fe III)-based OMS-MIL-100 (Fe) as an efficient green catalyst for the aza-Michael reaction. *Catalysis Letters* **2018**, *148*, 2918–2928.

(S20) Newkirk, A.; Aliferis, I. Drying and decomposition of sodium carbonate. *Analytical chemistry* **1958**, *30*, 982–984.

(S21) McCullough, J. t.; Trueblood, K. The crystal structure of baddeleyite (monoclinic ZrO2). *Acta Crystallographica* **1959**, *12*, 507–511.

(S22) Howard, C. J.; Kisi, E. H.; Roberts, R. B.; Hill, R. J. Neutron diffraction studies of phase transformations between tetragonal and orthorhombic zirconia in magnesia-partially-stabilized zirconia. *Journal of the American Ceramic Society* **1990**, *73*, 2828–2833.

(S23) Guo, Z.; Du, F.; Li, G.; Cui, Z. Synthesis and characterization of single-crystal Ce (OH) CO3 and CeO2 triangular microplates. *Inorganic chemistry* **2006**, *45*, 4167–4169.

(S24) Abrahams, S.; Bernstein, J. Remeasurement of the structure of hexagonal ZnO. *Structural Science* **1969**, *25*, 1233–1236.

(S25) Callahan, B. P.; Wolfenden, R. Migration of methyl groups between aliphatic amines in water. *Journal of the American Chemical Society* **2003**, *125*, 310–311.

(S26) Liu, Y.; Schwartz, J. The species prepared from sodium borohydride and N, N-dimethylformamide reduces or dimethylaminates organic halides. *The Journal of Organic Chemistry* **1993**, *58*, 5005–5007.

(S27) Cromer, D. T.; Herrington, K. The structures of anatase and rutile. *Journal of the American Chemical Society* **1955**, *77*, 4708–4709.

(S28) Blake, R.; Hessevick, R.; Zoltai, T.; Finger, L. W. Refinement of the hematite structure. *American Mineralogist: Journal of Earth and Planetary Materials* **1966**, *51*, 123–129.

(S29) Hoffman, A. E.; Vanduyfhuys, L.; Nevjestic, I.; Wieme, J.; Rogge, S. M.; Depauw, H.; Van Der Voort, P.; Vrielinck, H.; Van Speybroeck, V. Elucidating the vibrational fingerprint of the flexible metal–organic framework MIL-53 (Al) using a combined experimental/computational approach. *The Journal of Physical Chemistry C* **2018**, *122*, 2734–2746.

(S30) Rudolph, M.; Motylenko, M.; Rafaja, D. Structure model of γ-Al2O3 based on planar defects. *IUCrJ* **2019**, *6*, 116–127.
